# Supplementary material for: Palmitoylation by ZDHHC4 inhibits TRPV1-mediated nociception
Source: EMBO Rep. 2024 Nov 11;26(1):101–21. doi: 10.1038/s44319-024-00317-0 (PMC11724110; doi:10.1038/s44319-024-00317-0)
Supplement: Supplementary file 1 — Appendix [file 44319_2024_317_MOESM1_ESM.pdf]

Appendix for

Palmitoylation by ZDHHC4 inhibits TRPV1-mediated nociception

Contents

Appendix Figure S1 2

Appendix Figure S2 3

Appendix Figure S3 5

Appendix Figure S4 7

Appendix Figure S5 9

Appendix Figure S1

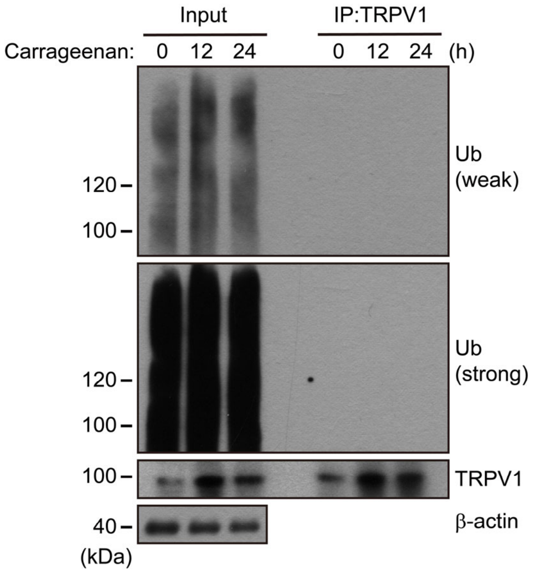

Appendix Figure S1. TRPV1 is not ubiquitinated during pain relief.

Immunoblotting assessing the ubiquitination of the total lysate (Input) and purified TRPV1 (IP: TRPV1) proteins obtained from DRGs of mice subjected to carrageenan (2%, w/v, in 20 μL saline) treatment. DRGs were dissociated 0 hrs, 12 hrs, or 24 hrs post carrageenan injection. Protein ubiquitination and TRPV1 were detected using the anti-Ub antibody and anti-TRPV1 antibody, respectively. β-actin served as the loading control. Weak or strong indicates blots with short or long exposure time, respectively.

Appendix Figure S2

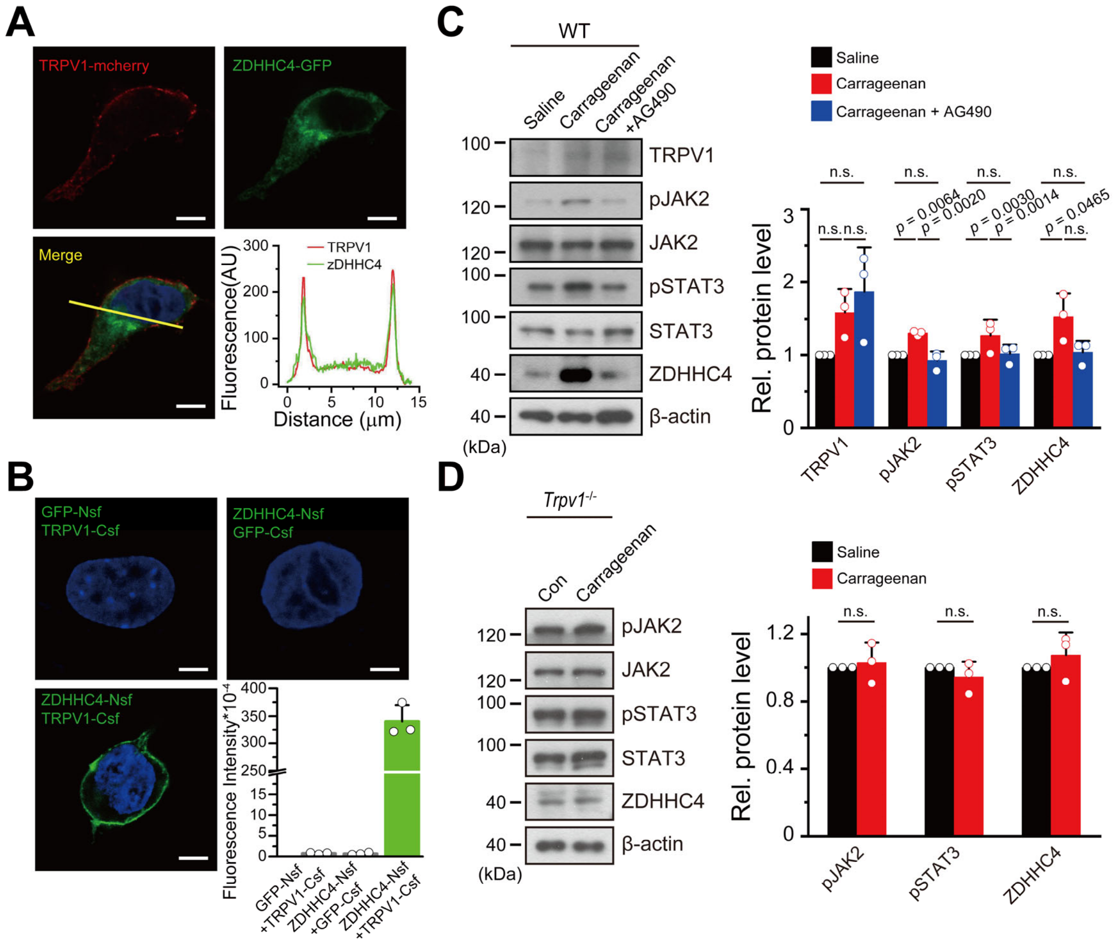

Appendix Figure S2. Interaction between ZDHHC4 and TRPV1 on the plasma membrane in *situ*.

(**A**) Confocal imaging revealed the co-localization of mCherry-tagged TRPV1 (mCherry-TRPV1, red) and GFP-tagged ZDHHC4 (GFP-ZDHHC4, green) in HEK293T cells, emphasizing their intense co-localization on the plasma membrane. In the merged images, the extensive co-localization of the two proteins was represented by yellow. Scale bar: 5 μm. (**B**) Bimolecular fluorescence complementation (BiFC) analysis demonstrated in-situ interactions between TRPV1 and ZDHHC4 in HEK293T cells. Confocal imaging revealed strong green fluorescence in ZDHHC4-NsfGFP and TRPV1-CsfGFP co-transfected cells on the

plasma membrane. Absence of fluorescence was observed in negative control groups (GFP-Nsf + TRPV1-Csf and ZDHHC4-Nsf + GFP-Csf). Blue fluorescence of DAPI indicates the nuclei. Scale bar: 5 μm. The bar chart provided a quantitative representation of the membrane fluorescence intensities (*n* = 3 cells for each group). **(C-D)** Effects of inhibition of JAK2 activity (C) or TRPV1-KO (D) on the phosphorylation of JAK2 and STAT3 and the protein level of ZDHHC4. (C) WT mice were administered AG490 or saline via intrathecal injection prior to the induction of a carrageenan-induced inflammation model. The protein levels of TRPV1 and the phosphorylation states of JAK2 and STAT3 in L4-L6 DRG neurons were assessed by immunoblotting (*n* = 3 mice for each group). The statistical significance was assessed using ONE-WAY ANOVA with post-hoc Turkey analysis. (D) Parallel experiments to detect phosphorylation states of JAK2 and STAT3 in L4-L6 DRG neurons of $Trpv1^{-/-}$ mice (*n* = 3 mice for each group). The statistical significance was assessed using unpaired student *t*-test.

Data information: In (B-D), data are presented as mean ± SD, each data point represents a separate biological replicate. The *p* values are illustrated in the figure. n.s., not significant.

Appendix Figure S3

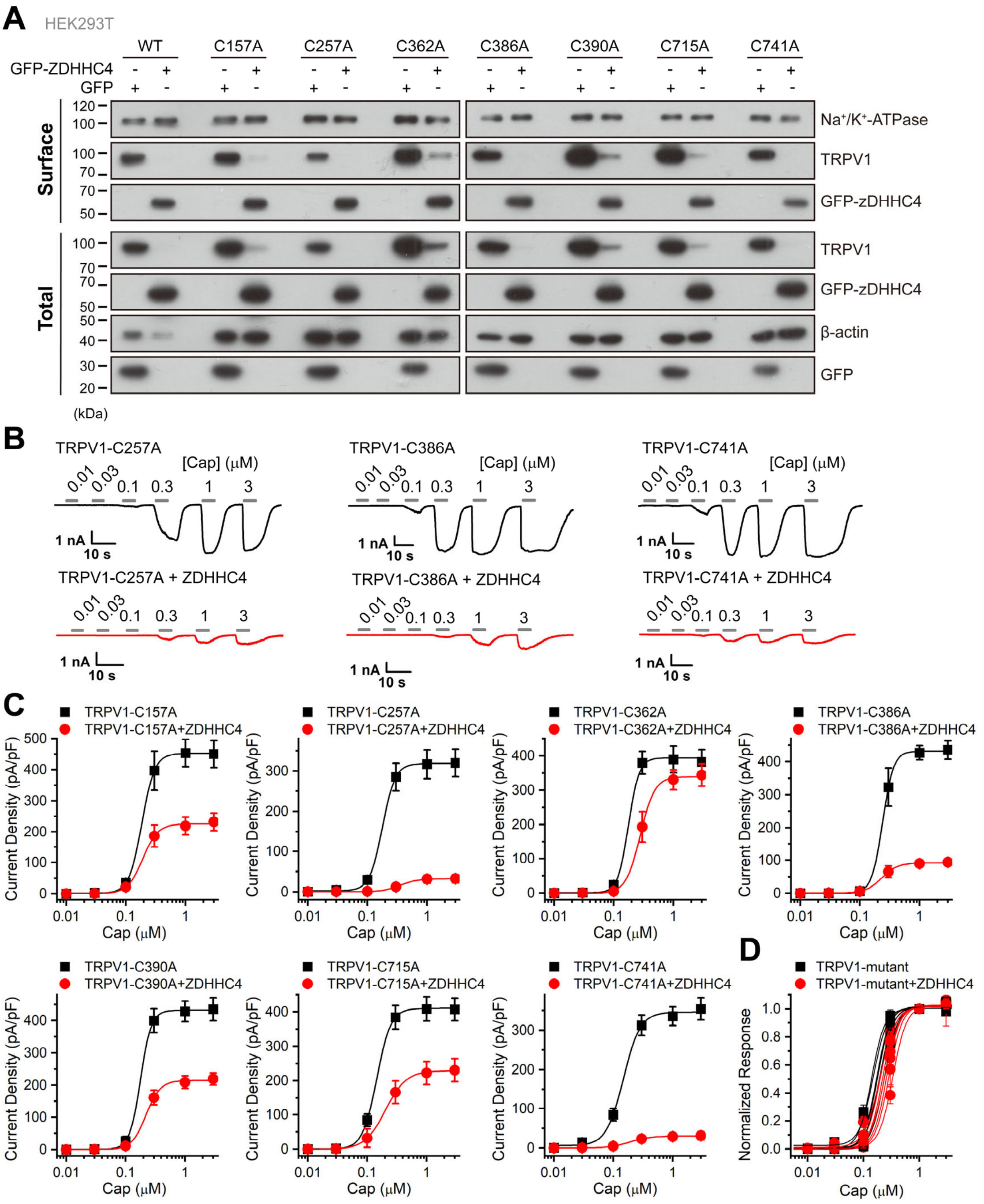

Appendix Figure S3. TRPV1 is palmitoylated at C157, C362, C390, and C715.

(**A**) Immunoblotting illustrating the C157A, C362A, C390A, and C715A mutations, but not the others, in TRPV1, partially rescued the downregulation of both the plasma

membrane and total TRPV1 protein levels by ZDHHC4 in HEK293T cells. GFP was utilized as the transfection control, while the Na^+/K^+ ATPase and β-actin served as the loading control for surface and total proteins, respectively. (**B**) Representative capsaicin (ranging from 0.01 to 3 μM)-evoked currents of HEK293T cells transfected with the C257A, C386A, and C741A TRPV1 mutants in the absence (*black*) or presence (*red*) of ZDHHC4. **(C-D)** Current-density-concentration relationships (C) and the normalized dose-response relationships (D) of capsaicin activation of the indicated TRPV1 mutants, under conditions with or without ZDHHC4 coexpression. The resulting values of EC_{50} and n_H are as follows: TRPV1/C157A (EC_{50} = 0.19 ± 0.004 μM, n_H = 3.58 ± 0.04; n = 5 cells); TRPV1/C157A+ZDHHC4 (EC_{50} = 0.20 ± 0.008 μM, n_H = 3.58 ± 0.30; n = 5 cells); TRPV1/C362A (EC_{50} = 0.27 ± 0.004 μM, n_H = 3.73 ± 0.14; n = 5 cells) ; TRPV1/C362A+ZDHHC4 (EC_{50} = 0.28 ± 0.007 μM, n_H = 3.57 ± 0.7; n = 5 cells) ; TRPV1/C390A (EC_{50} = 0.17 ± 0.004 μM, n_H = 3.73 ± 0.14; n = 8 cells); TRPV1/C390A+ZDHHC4 (EC_{50} = 0.22 ± 0.004 μM, n_H = 3.69 ± 0.18; n = 7 cells); TRPV1-C715A (EC_{50} = 0.14 ± 0.001 μM, n_H = 3.72 ± 0.02; n = 9 cells); TRPV1/C715A+ZDHHC4 (EC_{50} = 0.23 ± 0.008 μM, n_H = 3.33 ± 0.35; n = 9 cells); TRPV1/C257A (EC_{50} = 0.18 ± 0.006 μM, n_H = 3.73 ± 0.23; n = 6 cells) ; TRPV1/C257A+ZDHHC4 (EC_{50} = 0.33 ± 0.001 μM, n_H = 3.54 ± 0.006; n = 6 cells); TRPV1/C386A (EC_{50} = 0.24 ± 0.006 μM, n_H = 3.65 ± 0.34; n = 5 cells) ; TRPV1/C386A+ZDHHC4 (EC_{50} = 0.26 ± 0.007 μM, n_H = 3.76 ± 0.55; n = 6 cells); TRPV1/C741A (EC_{50} = 0.15 ± 0.007 μM, n_H = 3.07 ± 0.27; n = 7 cells); TRPV1/C741A+ZDHHC4 (EC_{50} = 0.18 ± 0.007 μM, n_H = 2.92 ± 0.20; n = 6 cells). Data information: In (C) and (D), data are presented as mean ± SD, n represents cell numbers for each group.

Appendix Figure S4

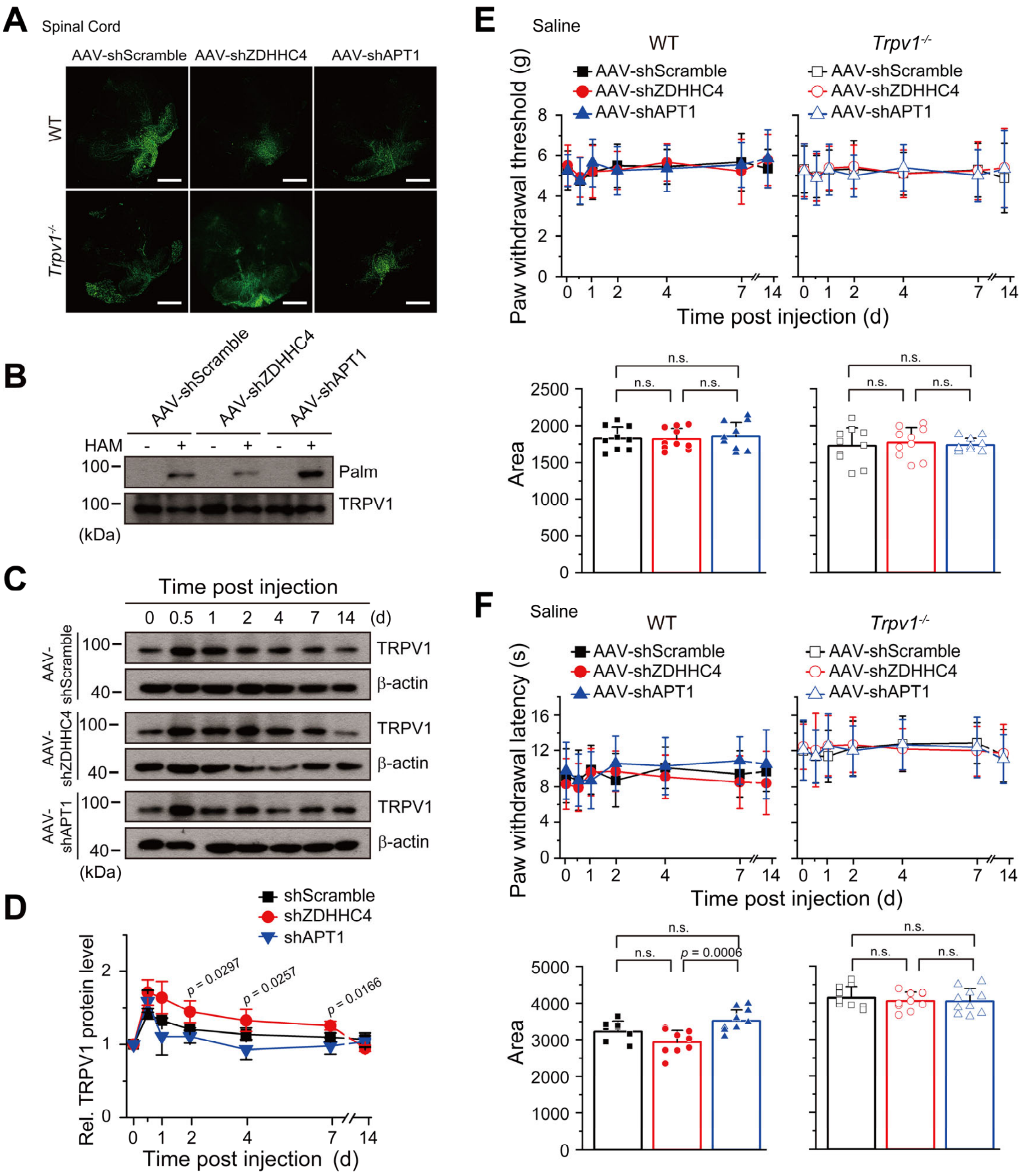

Appendix Figure S4. ZDHHC4 and APT1 regulate TRPV1 palmitoylation and protein levels in vivo.

(**A**) GFP fluorescence indicates successful transfection of indicated AAVs in spinal cord of wild-type (WT) or *Trpv1*$^{-/-}$ mice. Scale bar: 200 μm. (**B**) ABE assay, followed by immunoblotting, was employed to analyze the palmitoylation of TRPV1 in WT

mice injected with AAV-shScramble, AAV-shZDHHC4, and AAV-shAPT1, TRPV1 itself used as the loading control. (**C-D**) Immunoblotting (C) assessing the protein levels of TRPV1 in DRGs in WT mice infected with indicated AAVs, this evaluation was conducted at different time points following intraplantar injection of carrageenan to elicit inflammatory pain. β-actin serves as the loading control (n = 3, n represents biological replicates). The curves in (D) illustrate the dynamic protein levels of TRPV1 under the conditions shown in (C). The statistical significance was assessed using unpaired student *t*-test, p values represents significance between shZDHHC4 group and shAPT1 group. In these experiments, AAVs were administered intrathecally. **(E-F)** Paw withdrawal threshold (E) and latency (F) of WT and *Trpv1*$^{-/-}$ mice infected with the indicated AAVs. Two weeks post AAV infection, saline was injected and *Trpv1*$^{-/-}$ served as the control. Pain behavior was monitored for 14 days (n = 10 mice for each group). Upper panels show the time-dependent change in paw withdrawal threshold and latency; Lower panels illustrate the corresponding AUC (area under the curve) calculated from the curve shown in the upper panels. The statistical significance was assessed using ONE-WAY ANOVA with post-hoc Turkey analysis.

Data information: In (D-F), data are presented as mean ± SD. In (E) and (F), each data point represents a separate biological replicate. The p values are illustrated in the figure. n.s., not significant.

Appendix Figure S5

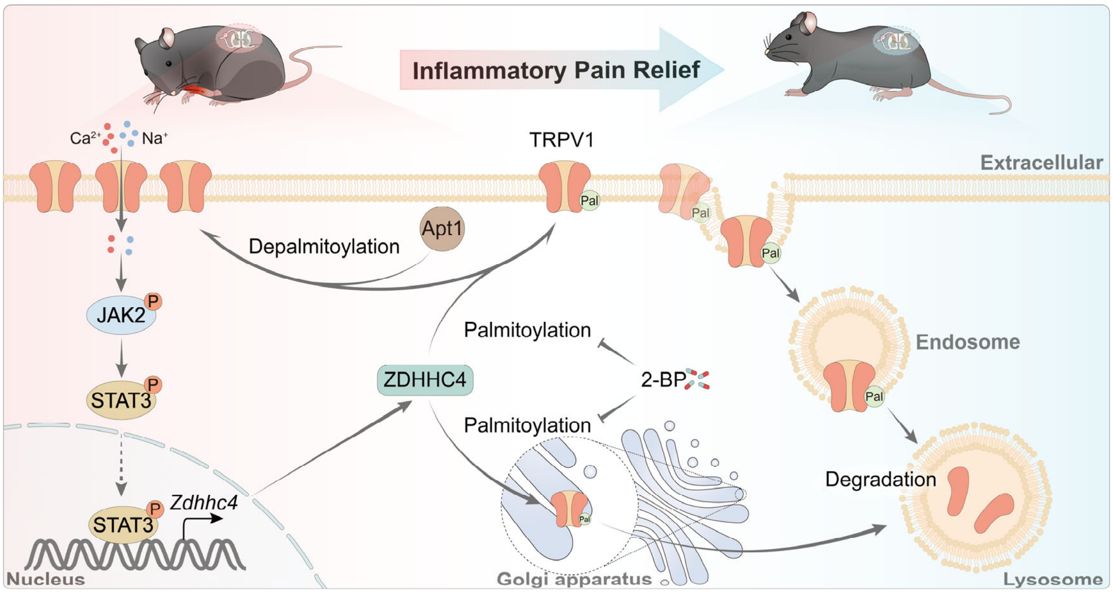

Appendix Figure S5. A schematic illustration of the proposed mechanism.

Enhanced TRPV1 activity in hyperalgesia activates JAK2-STAT3 signaling, potentially via Ca^{2+} influx, leading to nuclear translocation of phosphorylated STAT3 (p-STAT3) and expression of palmitoyl transferase ZDHHC4, ZDHHC4 interacts with and palmitoylates TRPV1, promoting its degradation for pain relief. APT1 counteracts TRPV1 palmitoylation, thereby stabilizing it on the plasma membrane.
