# Supplementary figures and images for: Palmitoylation by ZDHHC4 inhibits TRPV1-mediated nociception
Source: EMBO Rep. 2024 Nov 11;26(1):101–21. doi: 10.1038/s44319-024-00317-0 (PMC11724110; doi:10.1038/s44319-024-00317-0)

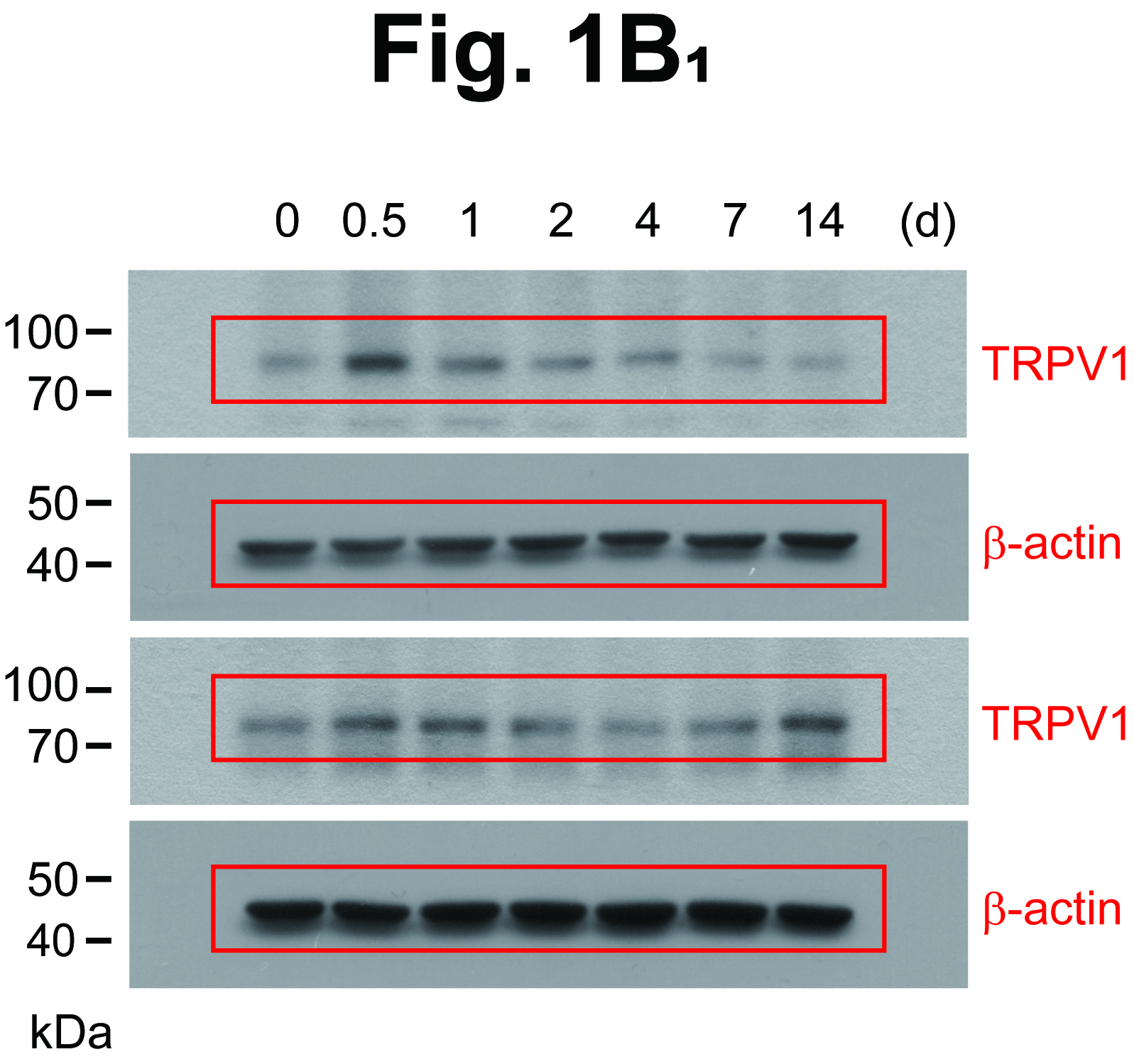

Supplement: Supplementary file 3 — Source data Fig. 1 [file 44319_2024_317_MOESM3_ESM.zip › 1C-data source.tif]

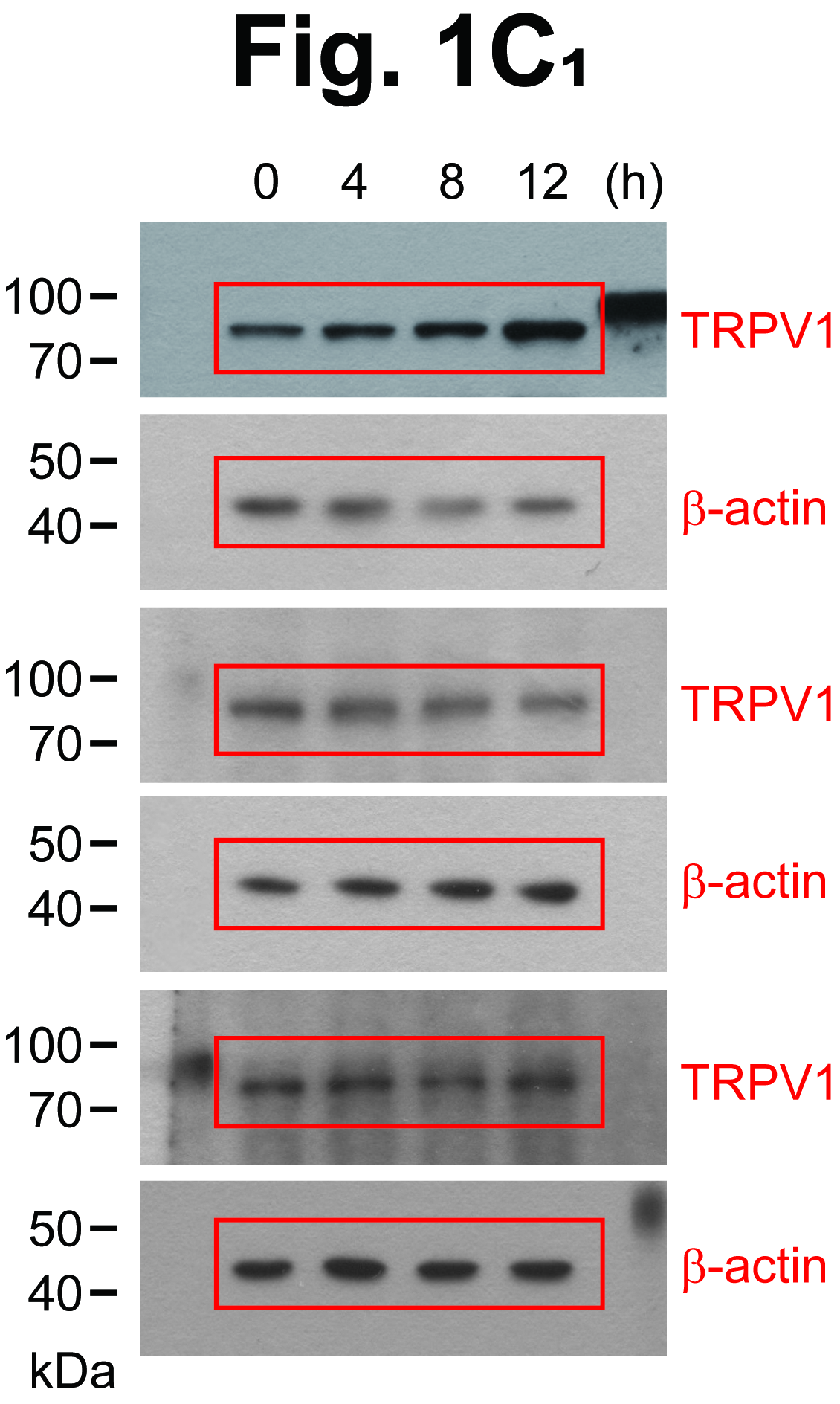

Supplement: Supplementary file 3 — Source data Fig. 1 [file 44319_2024_317_MOESM3_ESM.zip › 1E-data source.tif]

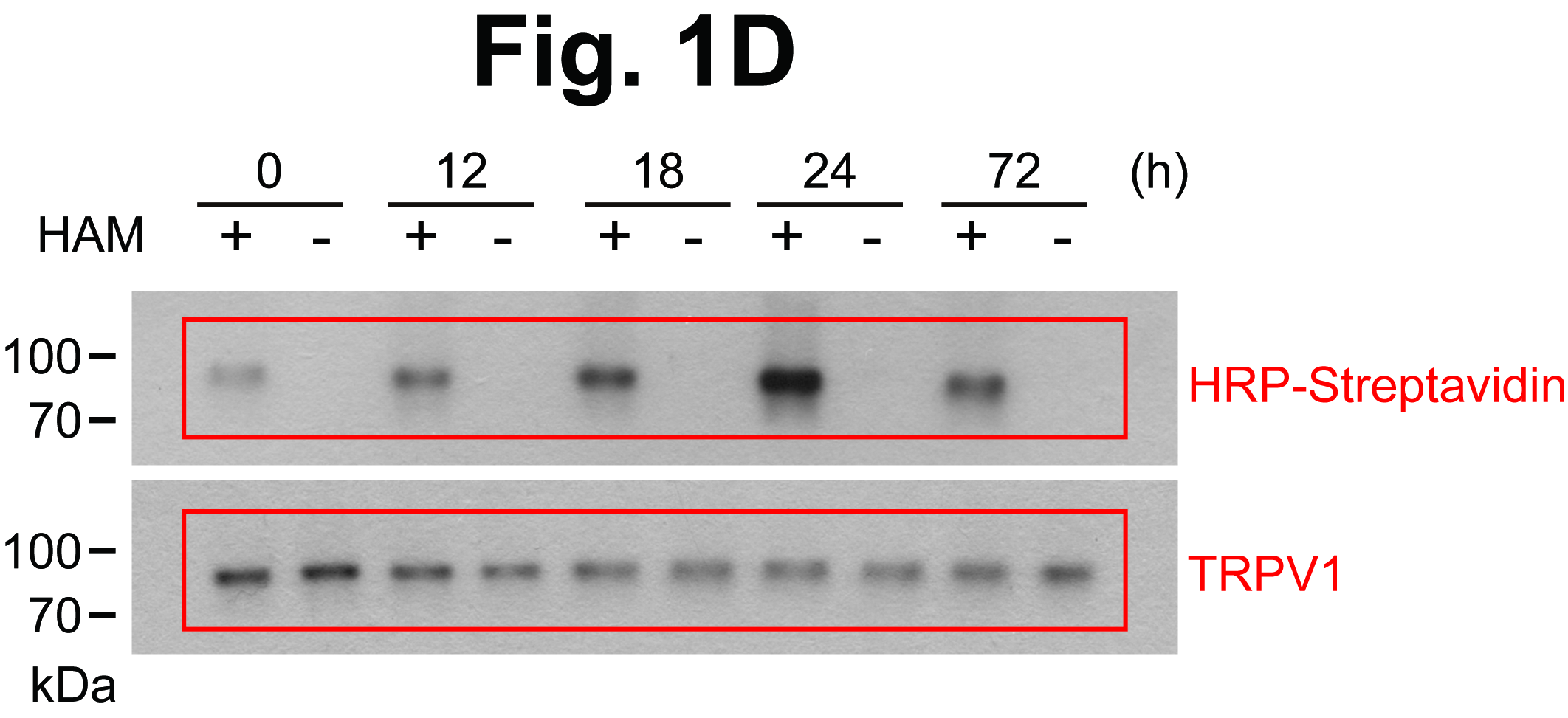

Supplement: Supplementary file 3 — Source data Fig. 1 [file 44319_2024_317_MOESM3_ESM.zip › 1G-data source.tif]

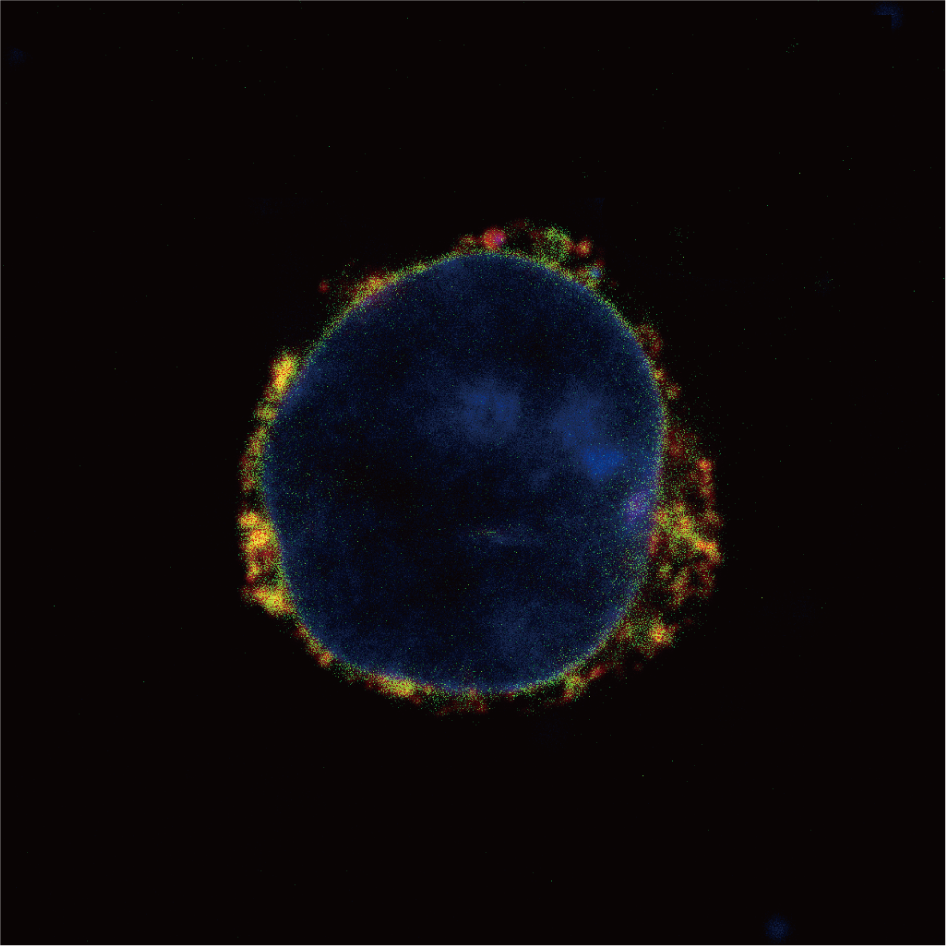

Supplement: Supplementary file 4 — Source data Fig. 2 [file 44319_2024_317_MOESM4_ESM.zip › 2G/2G_Carra-Merge.tif]

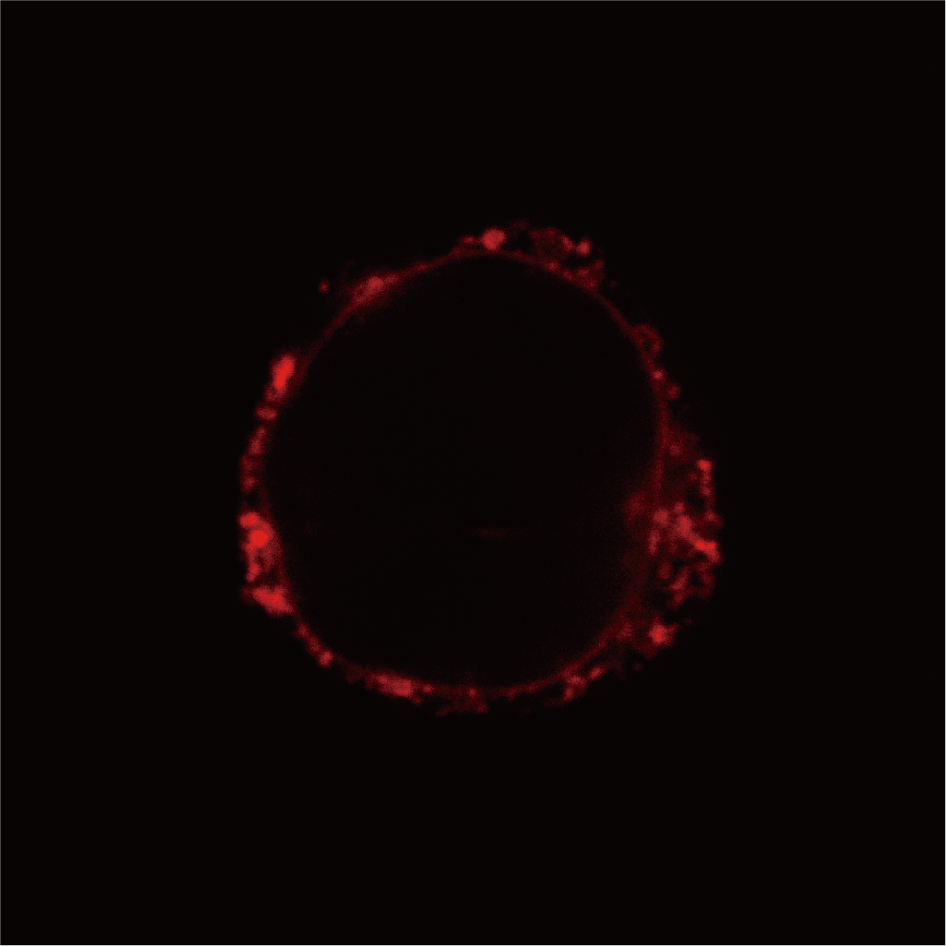

Supplement: Supplementary file 4 — Source data Fig. 2 [file 44319_2024_317_MOESM4_ESM.zip › 2G/2G_Carra-V1.tif]

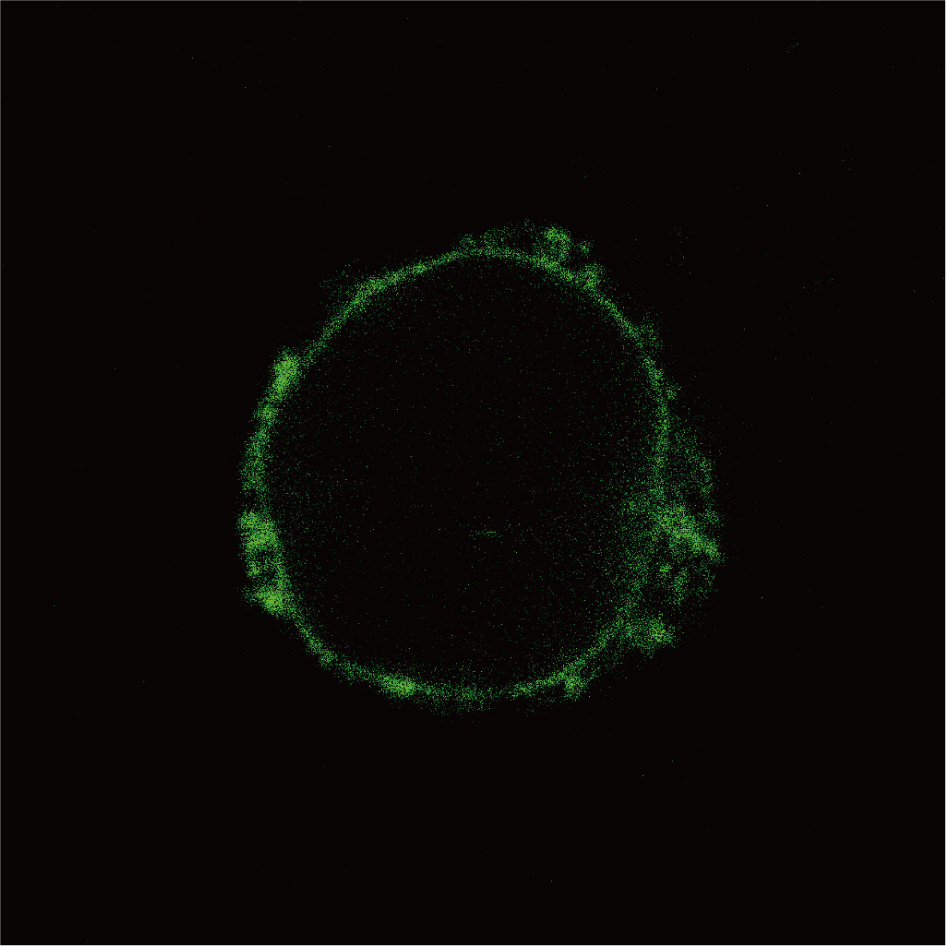

Supplement: Supplementary file 4 — Source data Fig. 2 [file 44319_2024_317_MOESM4_ESM.zip › 2G/2G_Carra-ZDHHC4.tif]

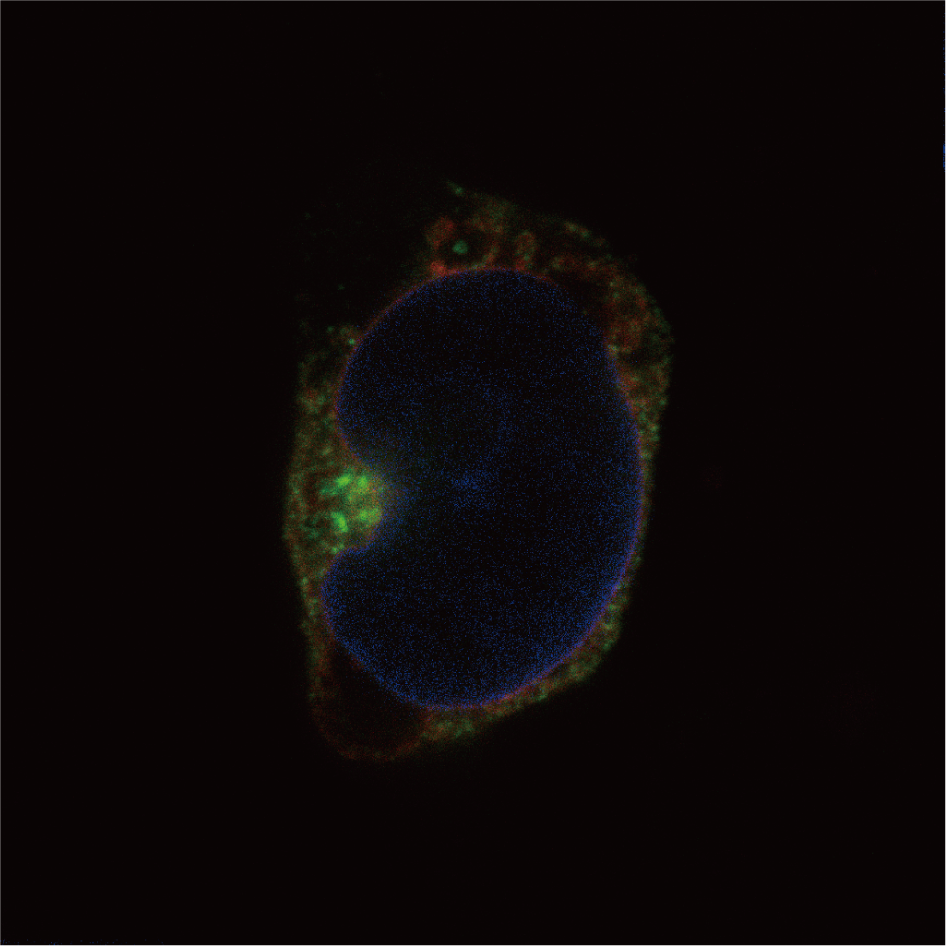

Supplement: Supplementary file 4 — Source data Fig. 2 [file 44319_2024_317_MOESM4_ESM.zip › 2G/2G_Saline-Merge.tif]

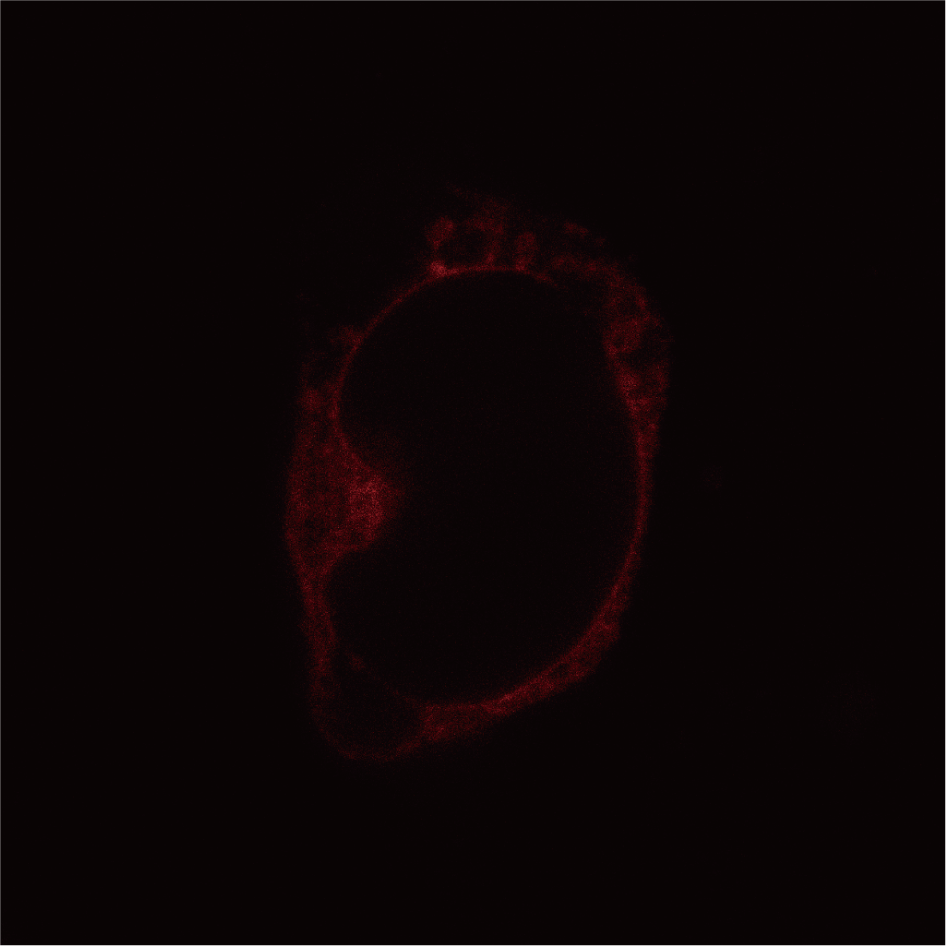

Supplement: Supplementary file 4 — Source data Fig. 2 [file 44319_2024_317_MOESM4_ESM.zip › 2G/2G_Saline-V1.tif]

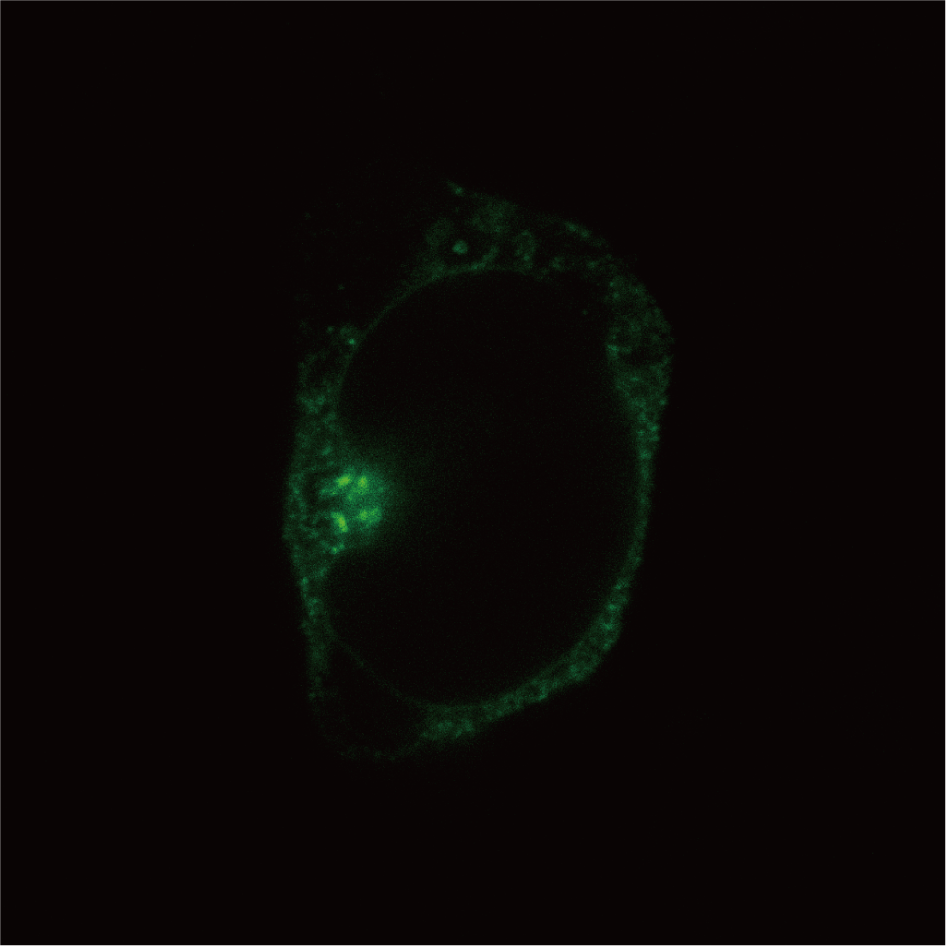

Supplement: Supplementary file 4 — Source data Fig. 2 [file 44319_2024_317_MOESM4_ESM.zip › 2G/2G_Saline-ZDHHC4.tif]

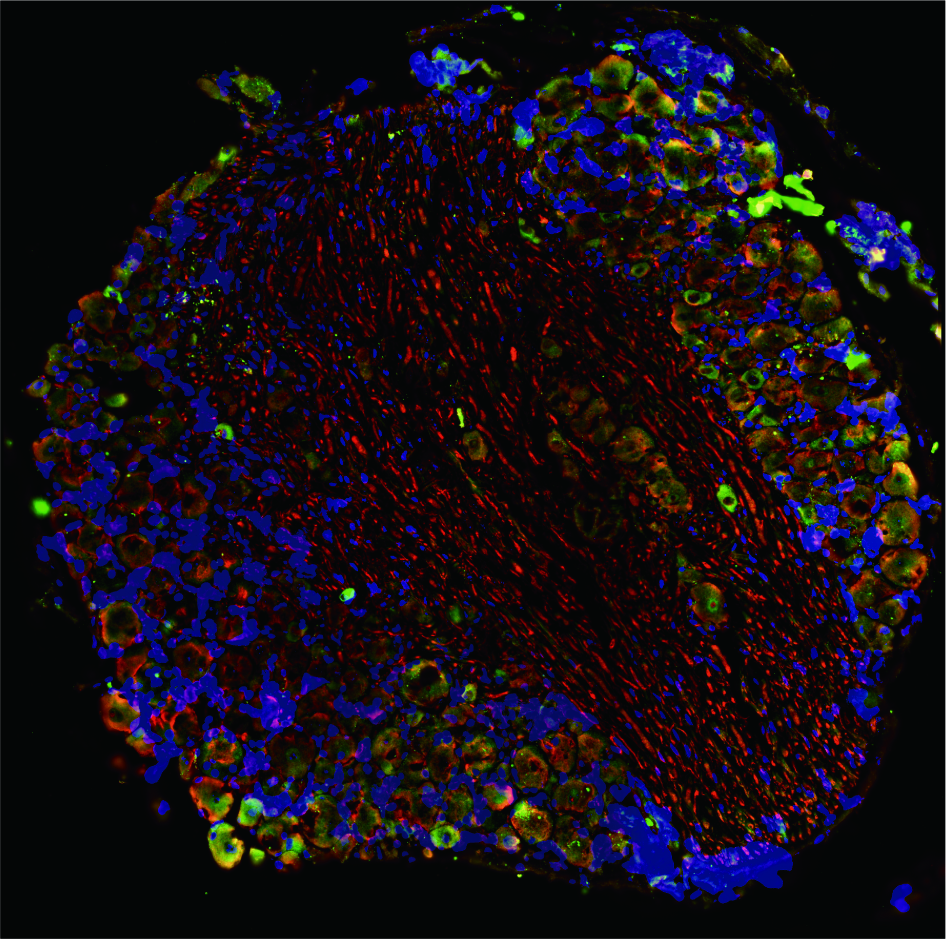

Supplement: Supplementary file 4 — Source data Fig. 2 [file 44319_2024_317_MOESM4_ESM.zip › 2H/2H_Carra_Merge.tif]

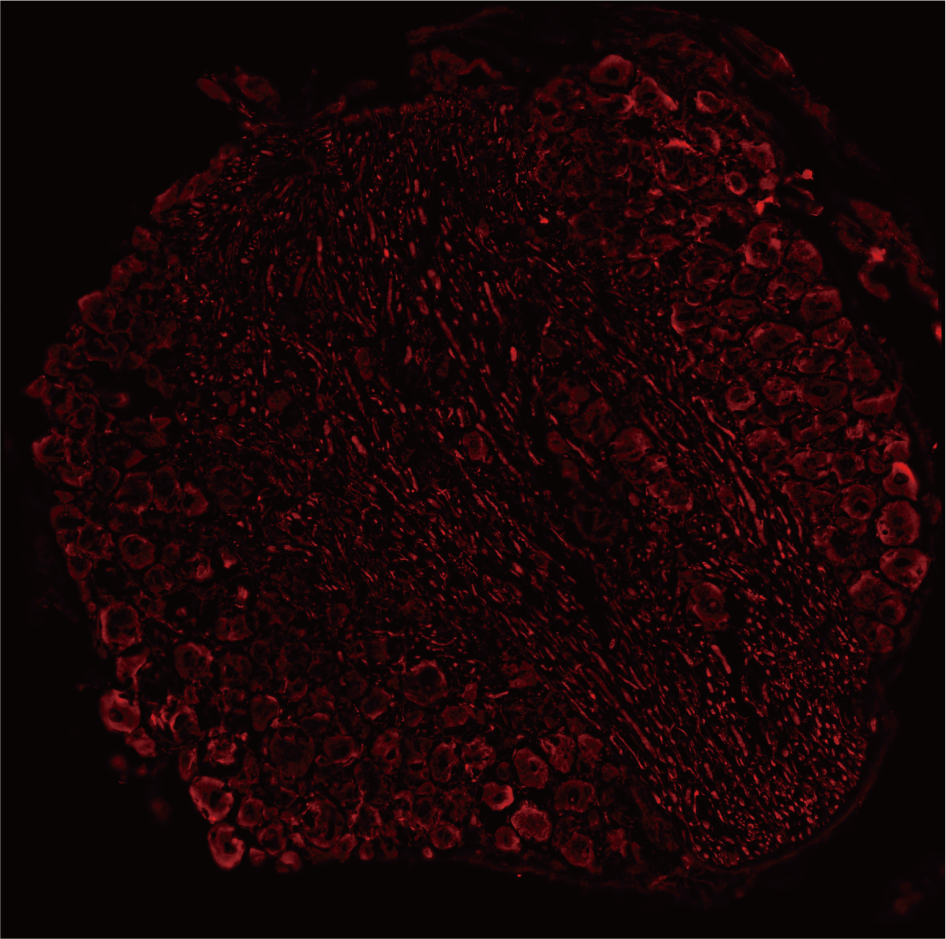

Supplement: Supplementary file 4 — Source data Fig. 2 [file 44319_2024_317_MOESM4_ESM.zip › 2H/2H_Carra_V1.tif]

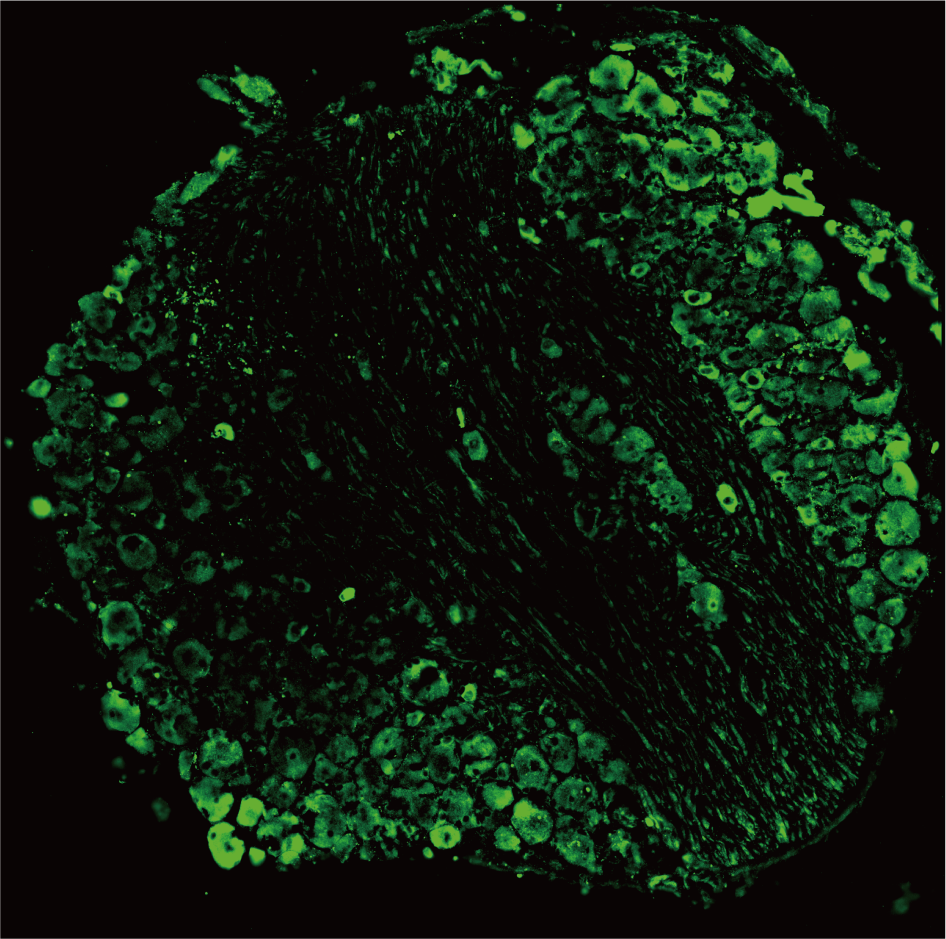

Supplement: Supplementary file 4 — Source data Fig. 2 [file 44319_2024_317_MOESM4_ESM.zip › 2H/2H_Carra_ZDHHC4.tif]

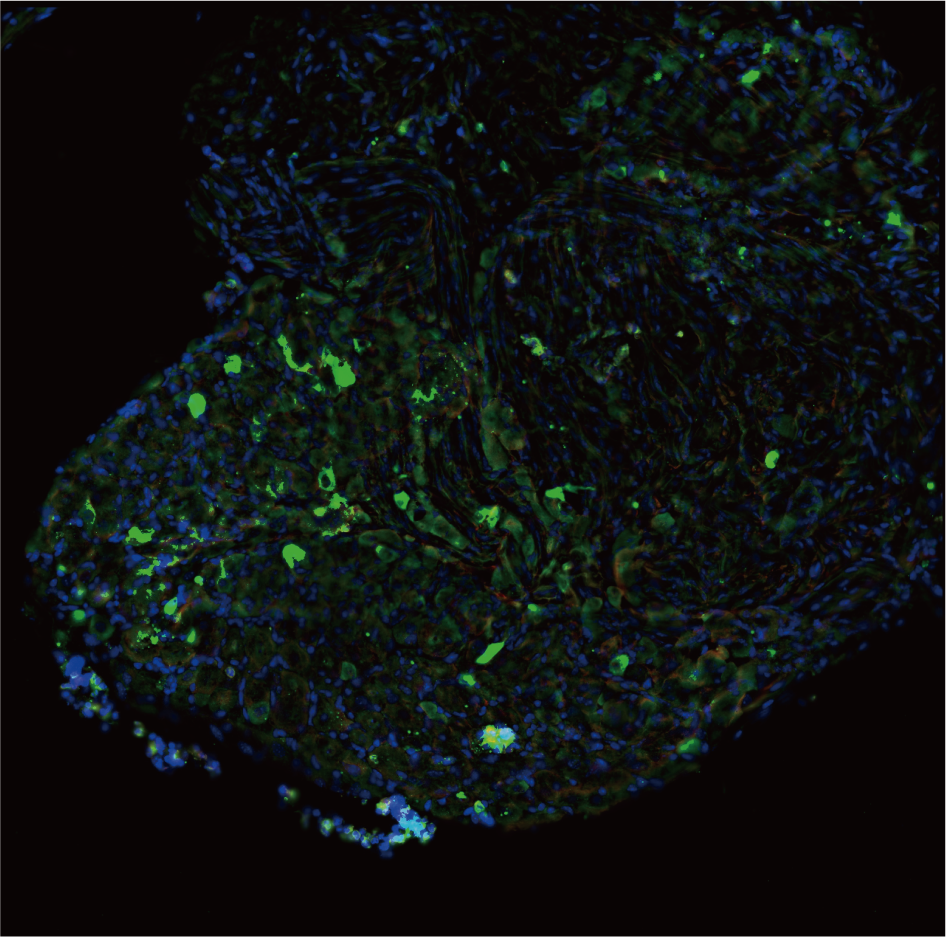

Supplement: Supplementary file 4 — Source data Fig. 2 [file 44319_2024_317_MOESM4_ESM.zip › 2H/2H_Saline_Merge.tif]

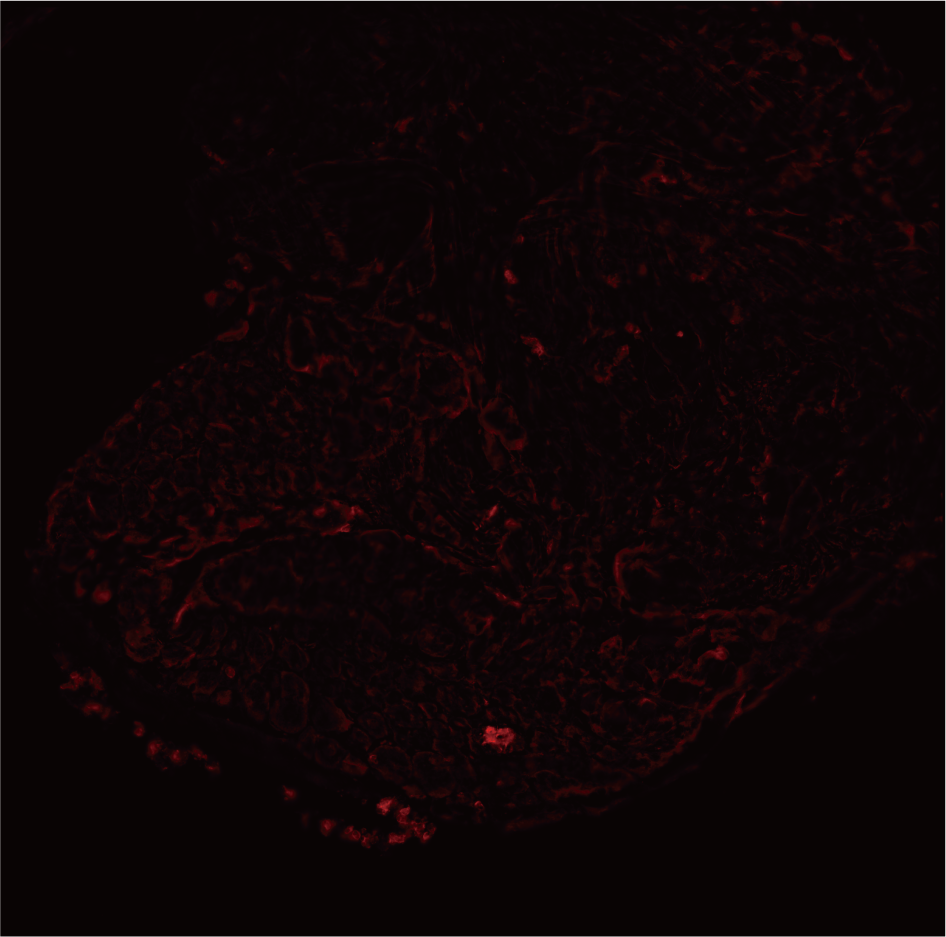

Supplement: Supplementary file 4 — Source data Fig. 2 [file 44319_2024_317_MOESM4_ESM.zip › 2H/2H_Saline_V1.tif]

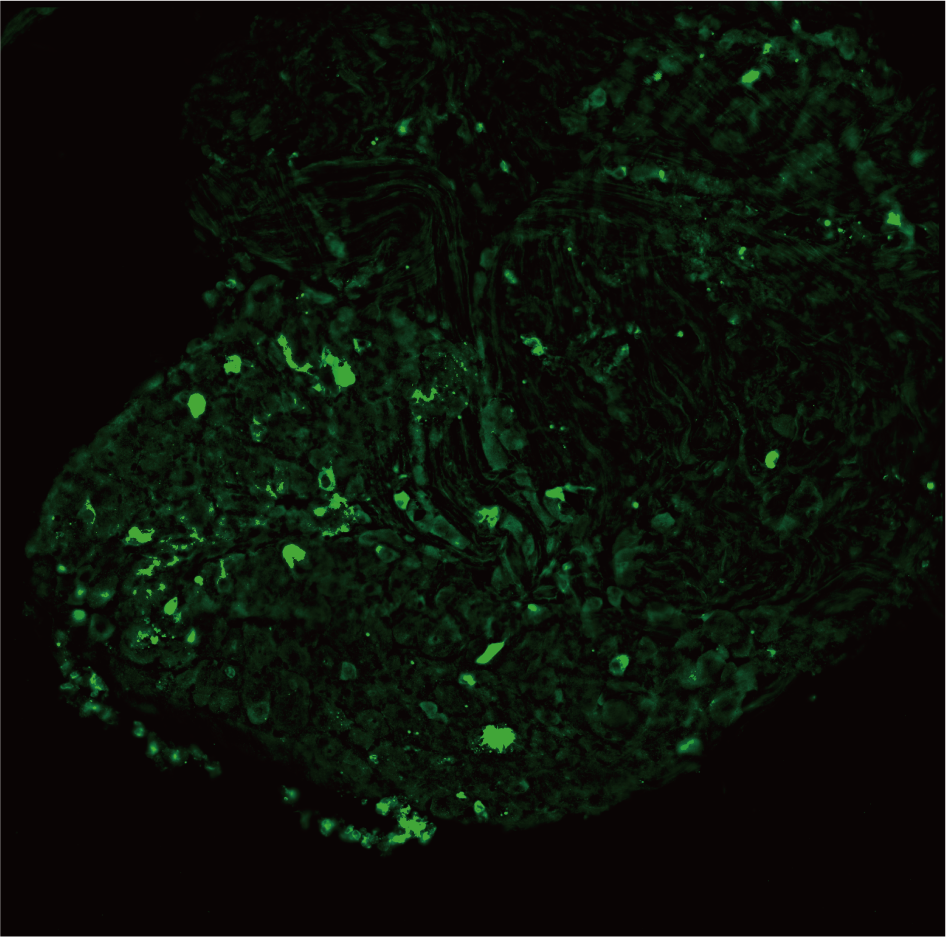

Supplement: Supplementary file 4 — Source data Fig. 2 [file 44319_2024_317_MOESM4_ESM.zip › 2H/2H_Saline_ZDHHC4.tif]

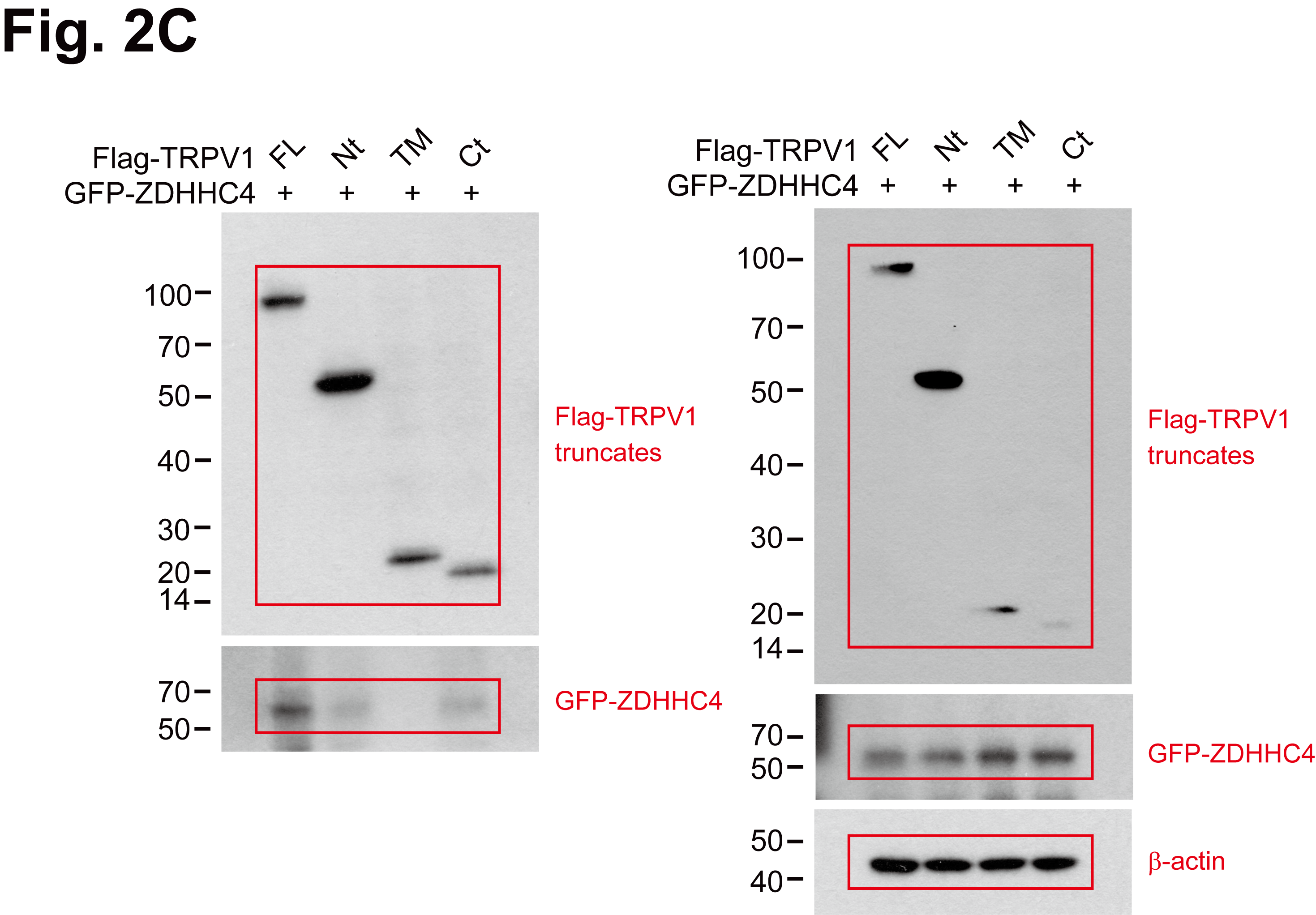

Supplement: Supplementary file 4 — Source data Fig. 2 [file 44319_2024_317_MOESM4_ESM.zip › 2C_data source.tif]

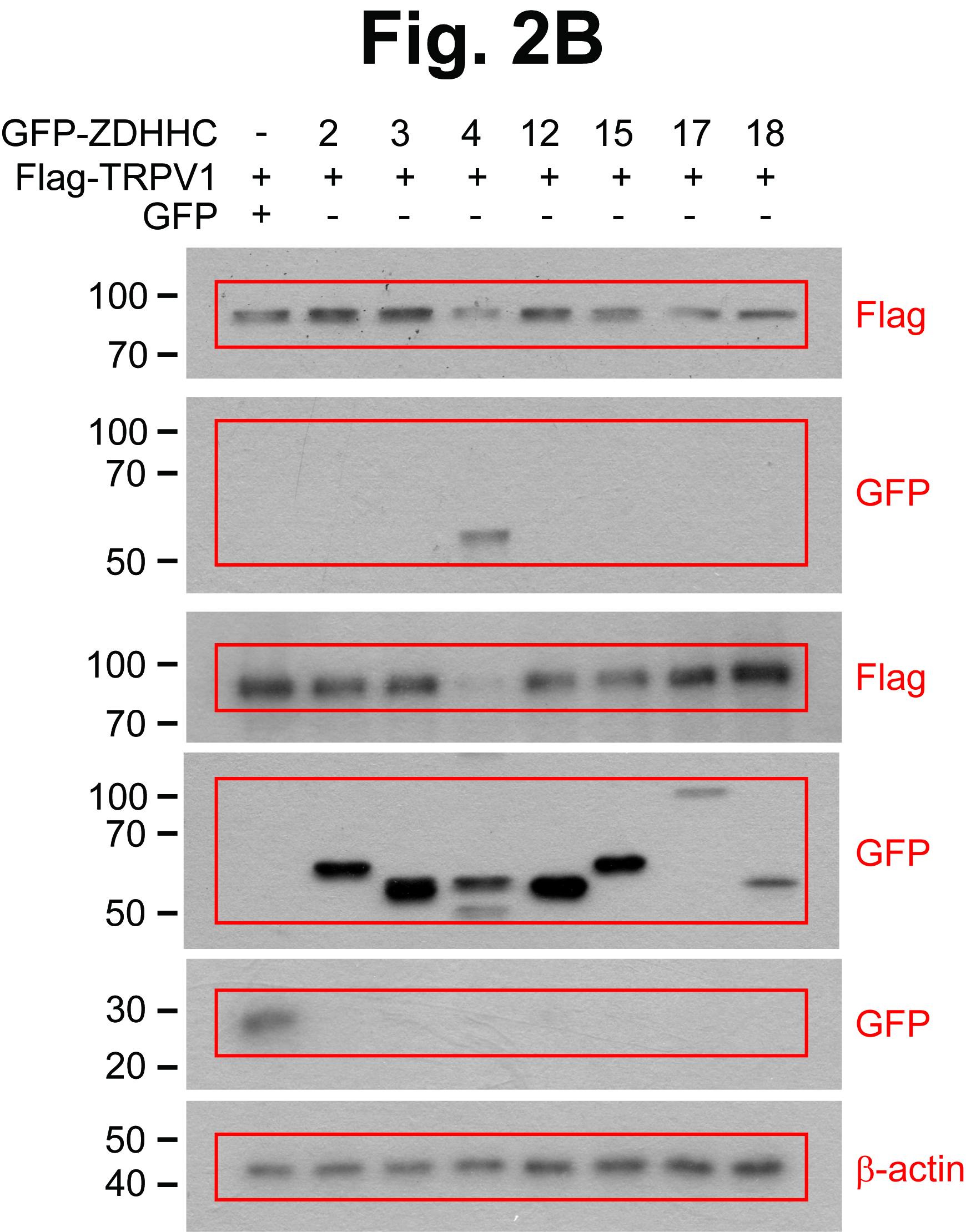

Supplement: Supplementary file 4 — Source data Fig. 2 [file 44319_2024_317_MOESM4_ESM.zip › 2B_data source.tif]

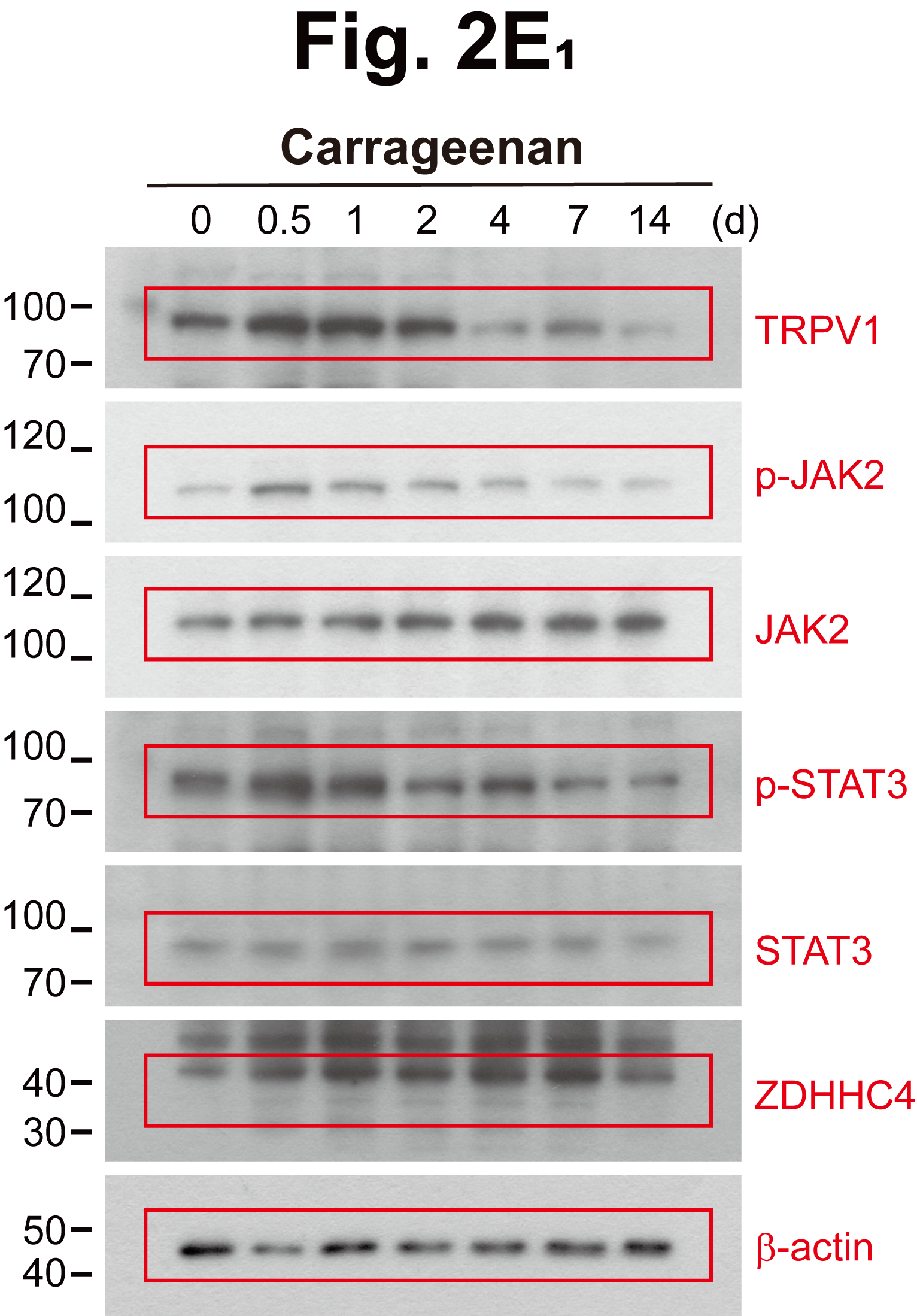

Supplement: Supplementary file 4 — Source data Fig. 2 [file 44319_2024_317_MOESM4_ESM.zip › 2E_data source.tif]

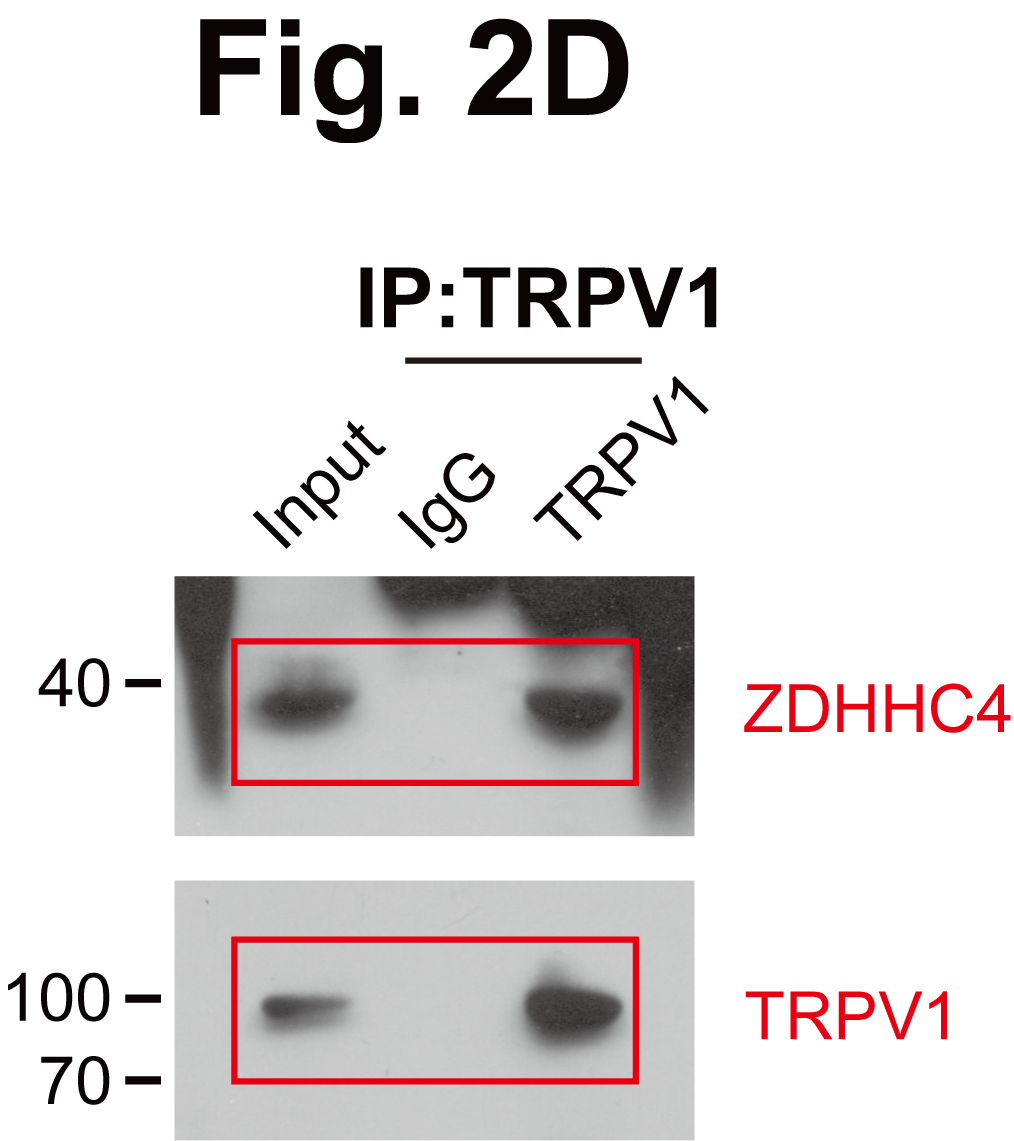

Supplement: Supplementary file 4 — Source data Fig. 2 [file 44319_2024_317_MOESM4_ESM.zip › 2D_ data source.tif]

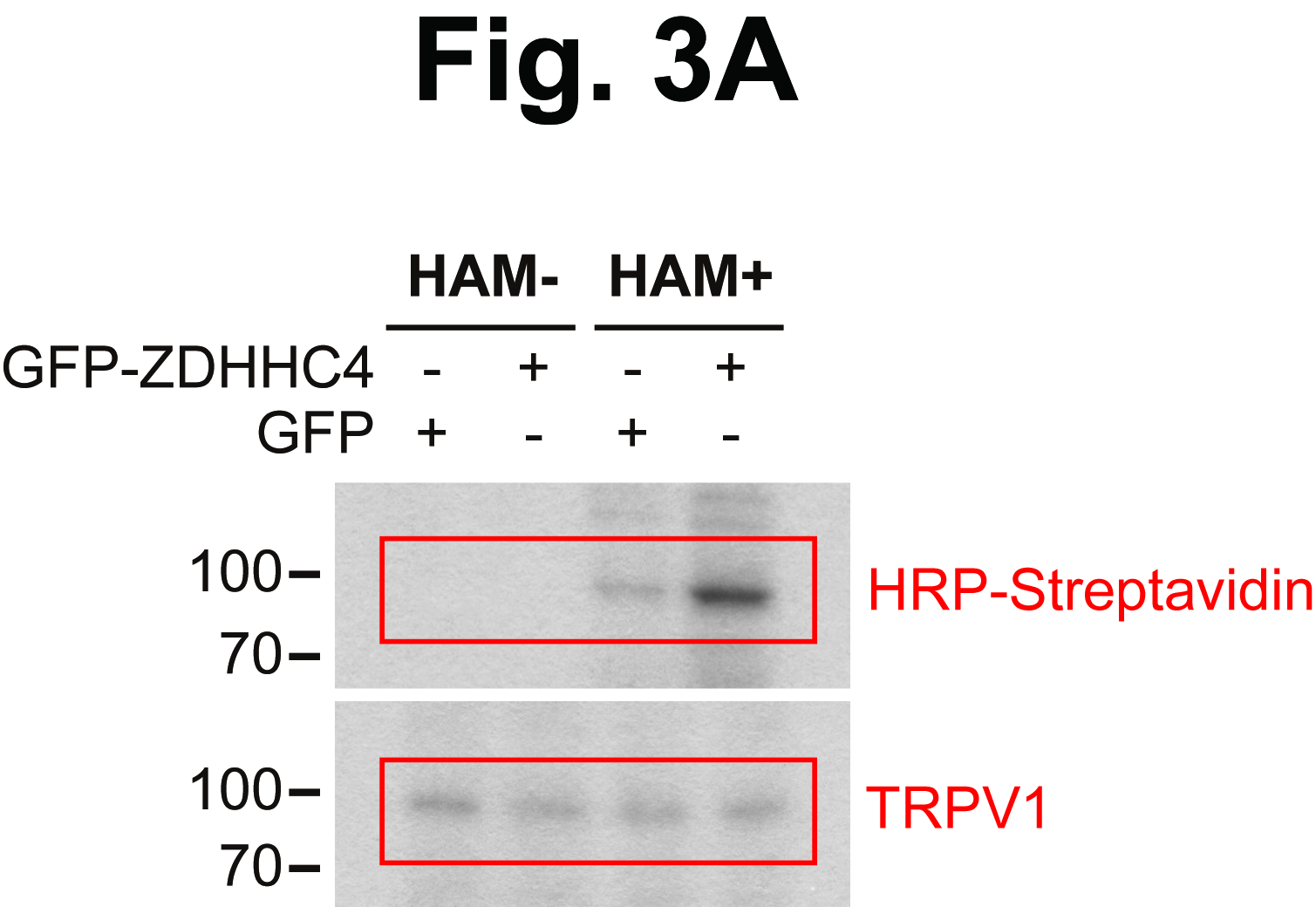

Supplement: Supplementary file 5 — Source data Fig. 3 [file 44319_2024_317_MOESM5_ESM.zip › 3A/Fig3A-data source.tif]

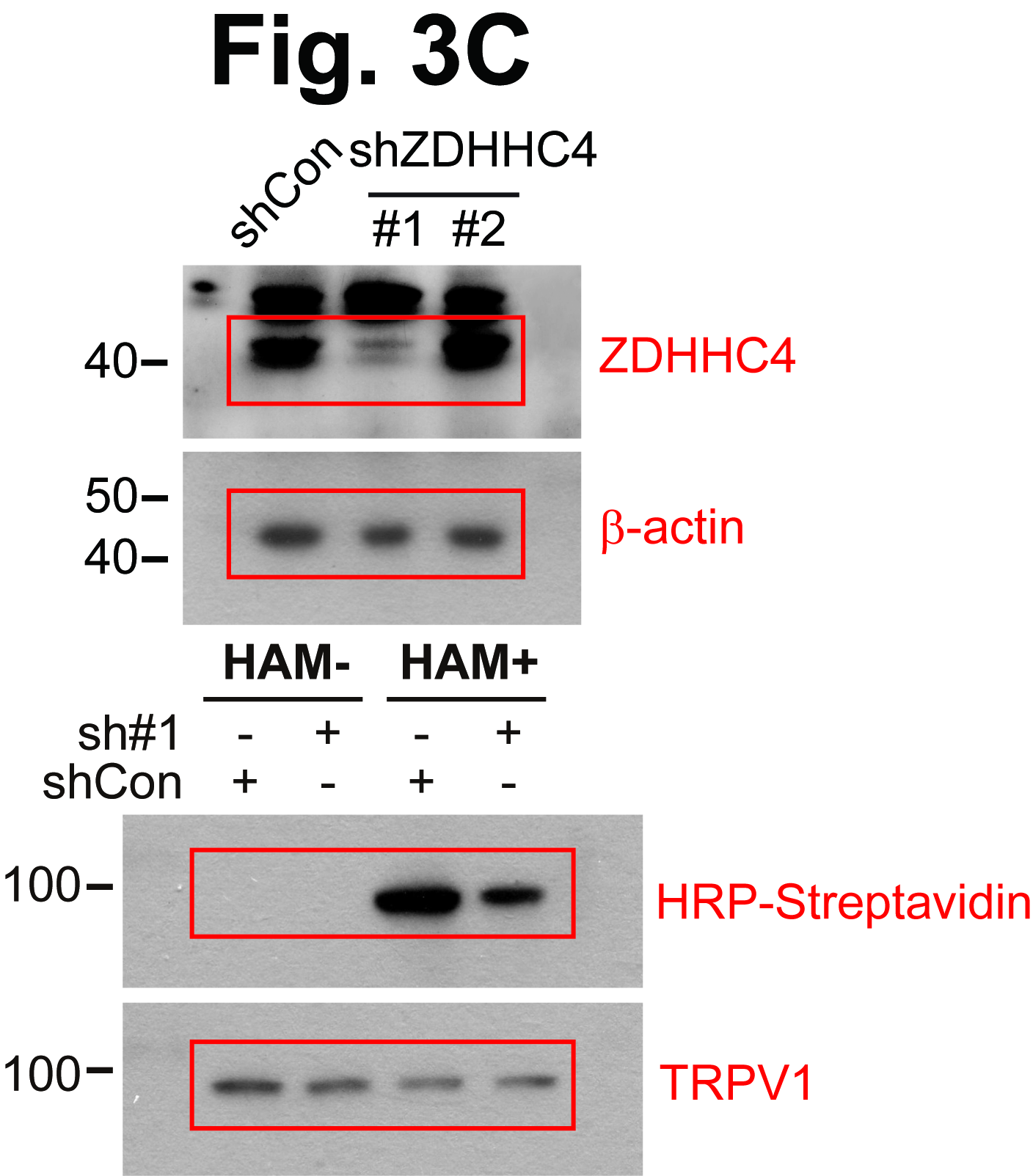

Supplement: Supplementary file 5 — Source data Fig. 3 [file 44319_2024_317_MOESM5_ESM.zip › 3C/Figure 3C-data source.tif]

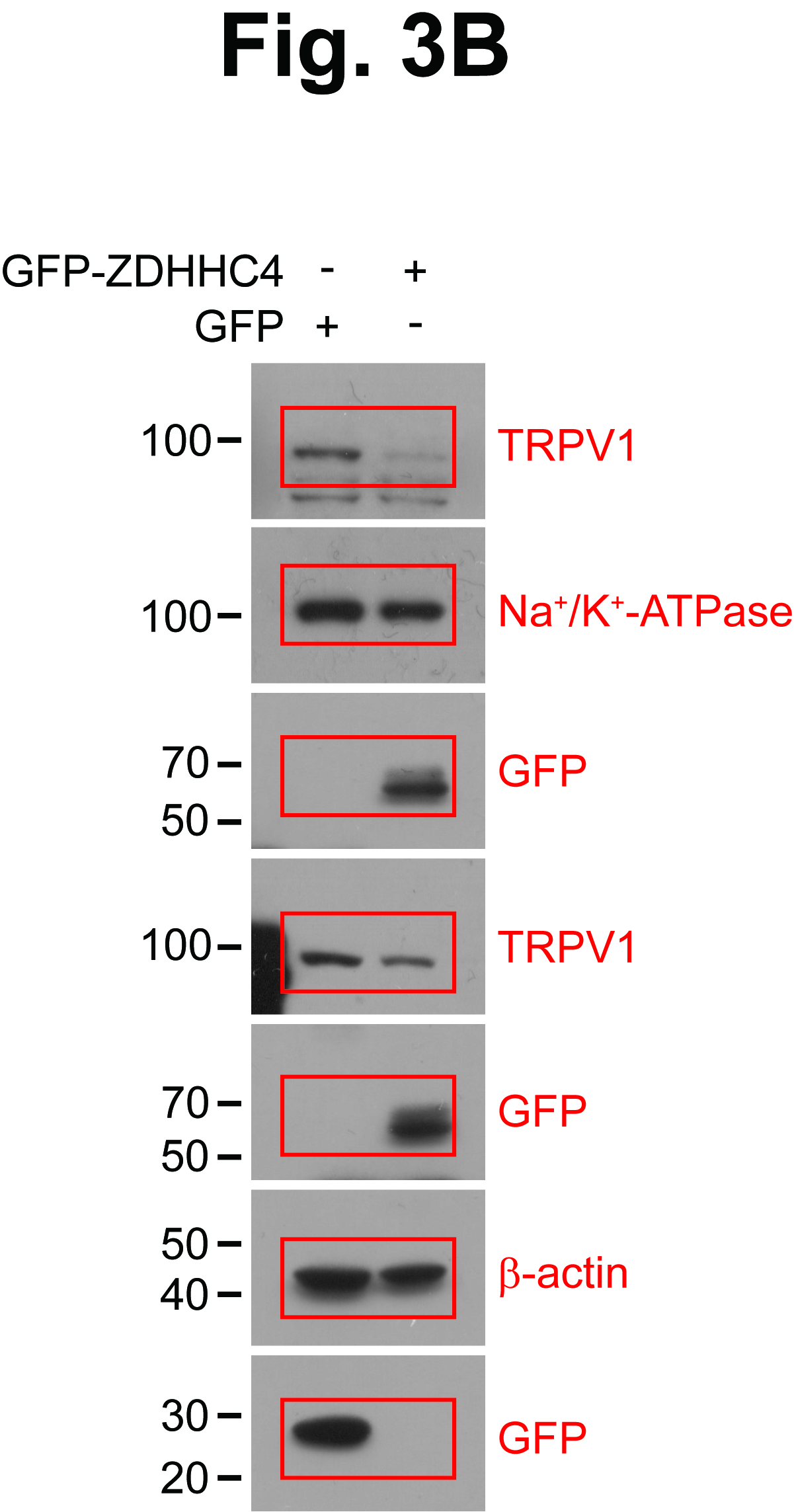

Supplement: Supplementary file 5 — Source data Fig. 3 [file 44319_2024_317_MOESM5_ESM.zip › 3B/Figure 3B-data source.tif]

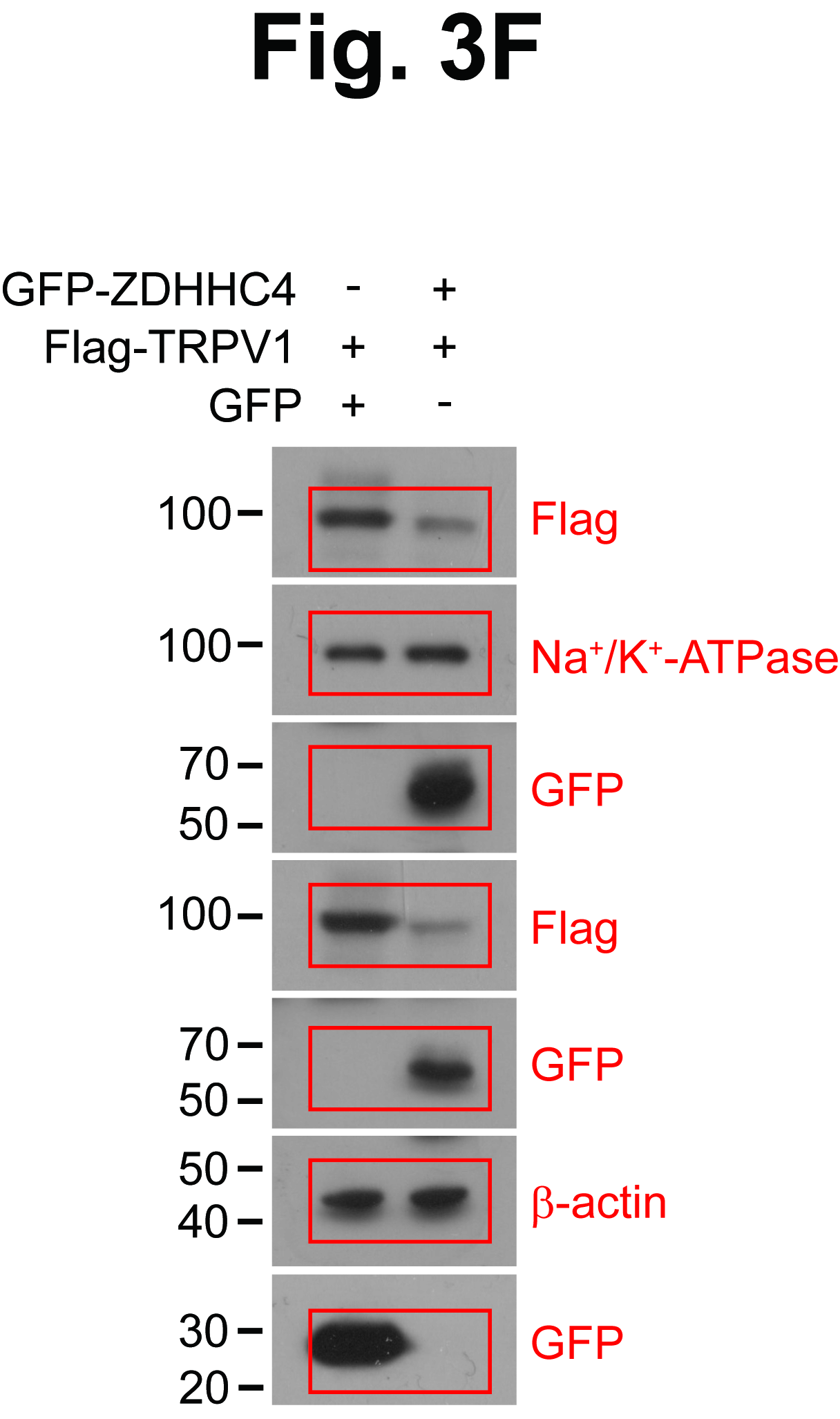

Supplement: Supplementary file 5 — Source data Fig. 3 [file 44319_2024_317_MOESM5_ESM.zip › 3F/Figure 3F-data source.tif]

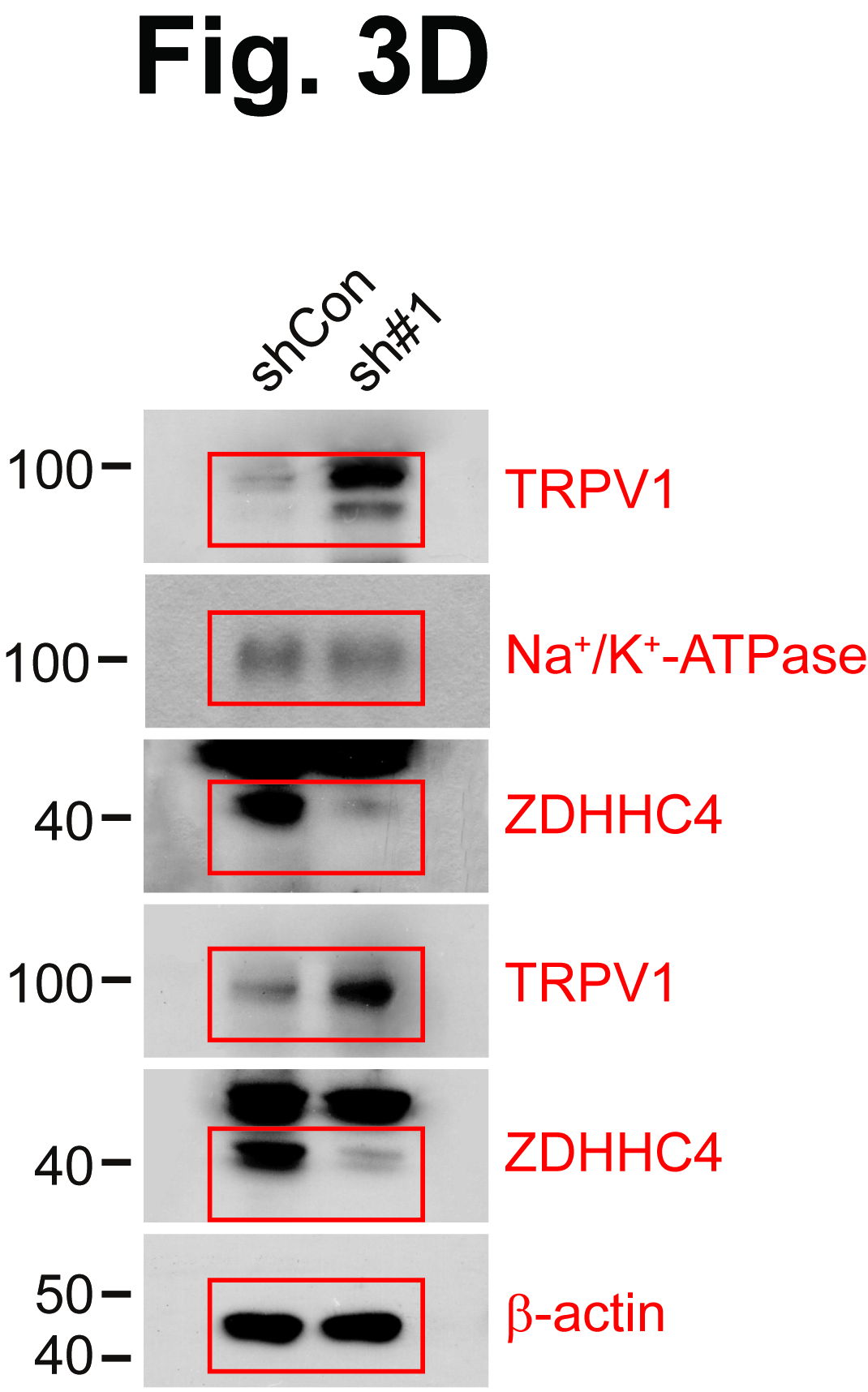

Supplement: Supplementary file 5 — Source data Fig. 3 [file 44319_2024_317_MOESM5_ESM.zip › 3D/Figure 3D-data source.tif]

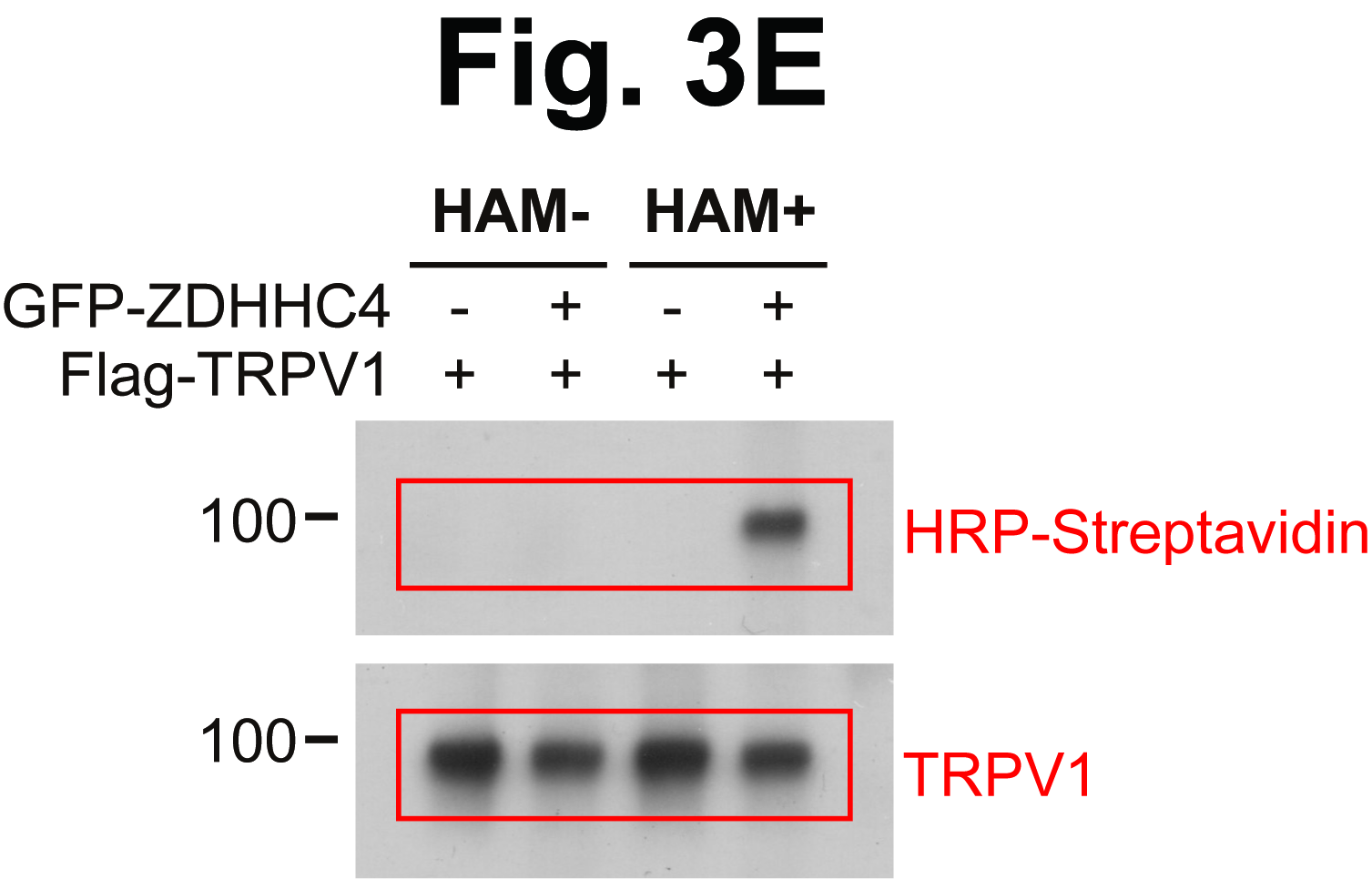

Supplement: Supplementary file 5 — Source data Fig. 3 [file 44319_2024_317_MOESM5_ESM.zip › 3E-data source.tif]

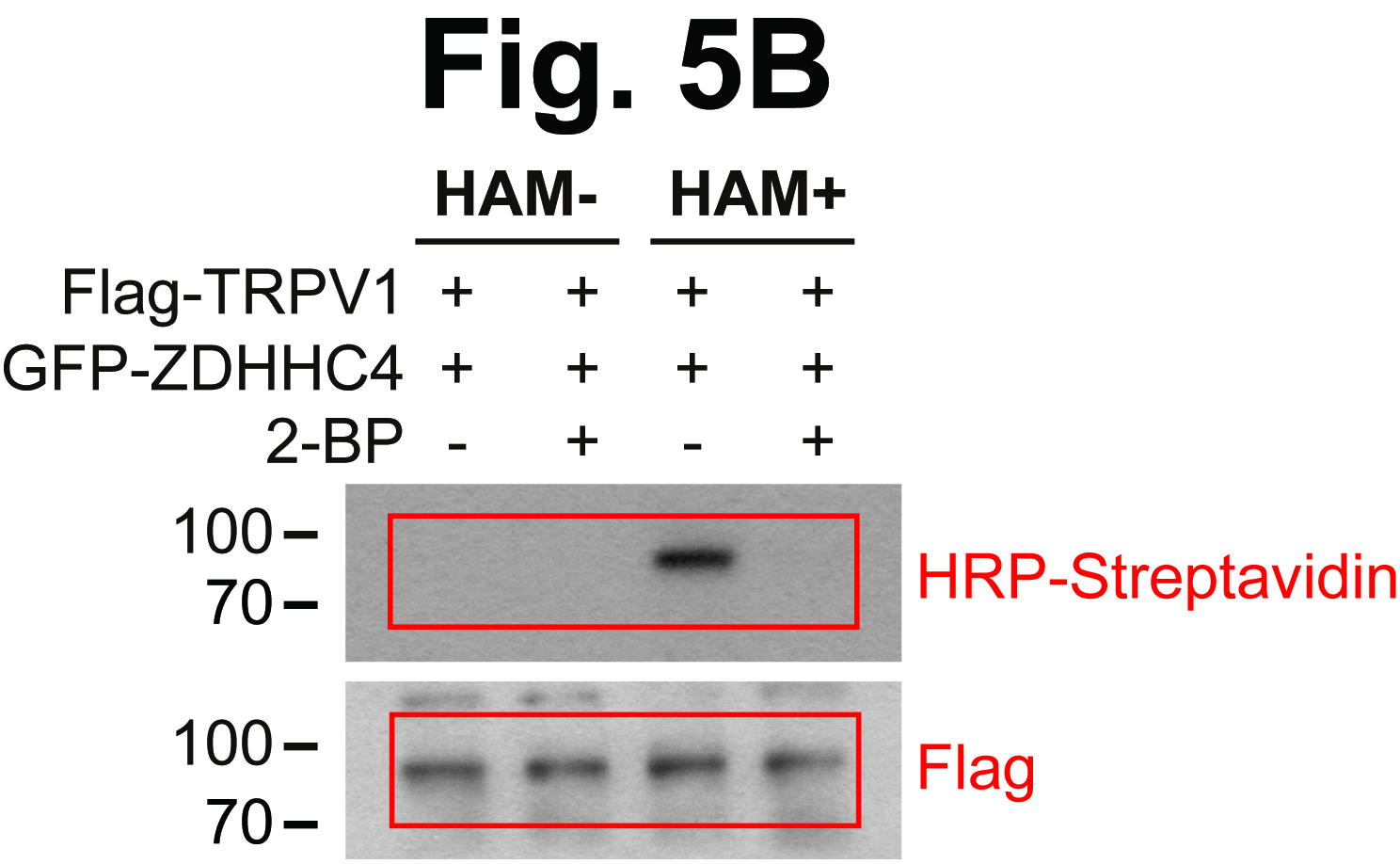

Supplement: Supplementary file 7 — Source data Fig. 5 [file 44319_2024_317_MOESM7_ESM.zip › 5B-data source.tif]

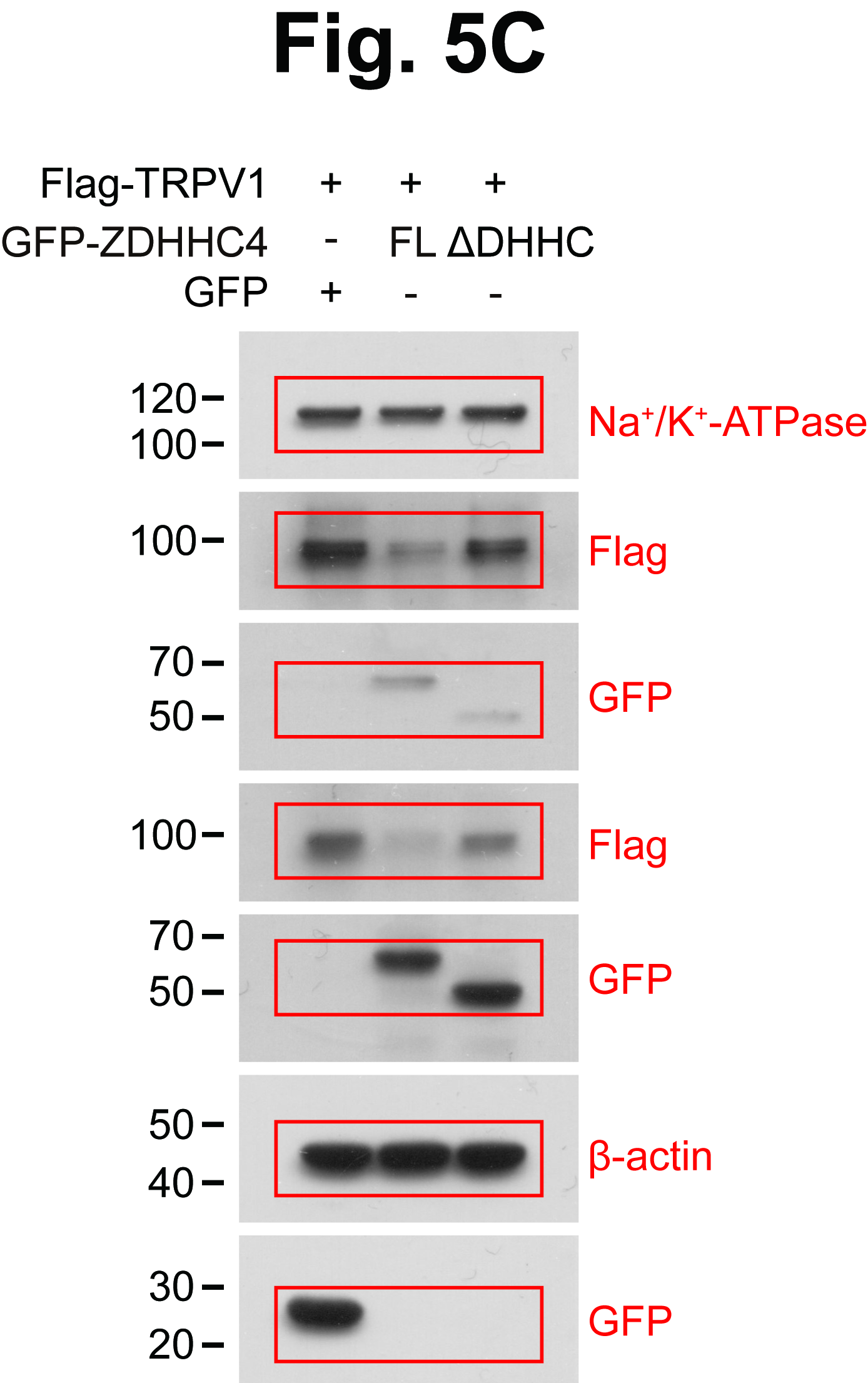

Supplement: Supplementary file 7 — Source data Fig. 5 [file 44319_2024_317_MOESM7_ESM.zip › 5C/Gray Analysis of 5C.tif]

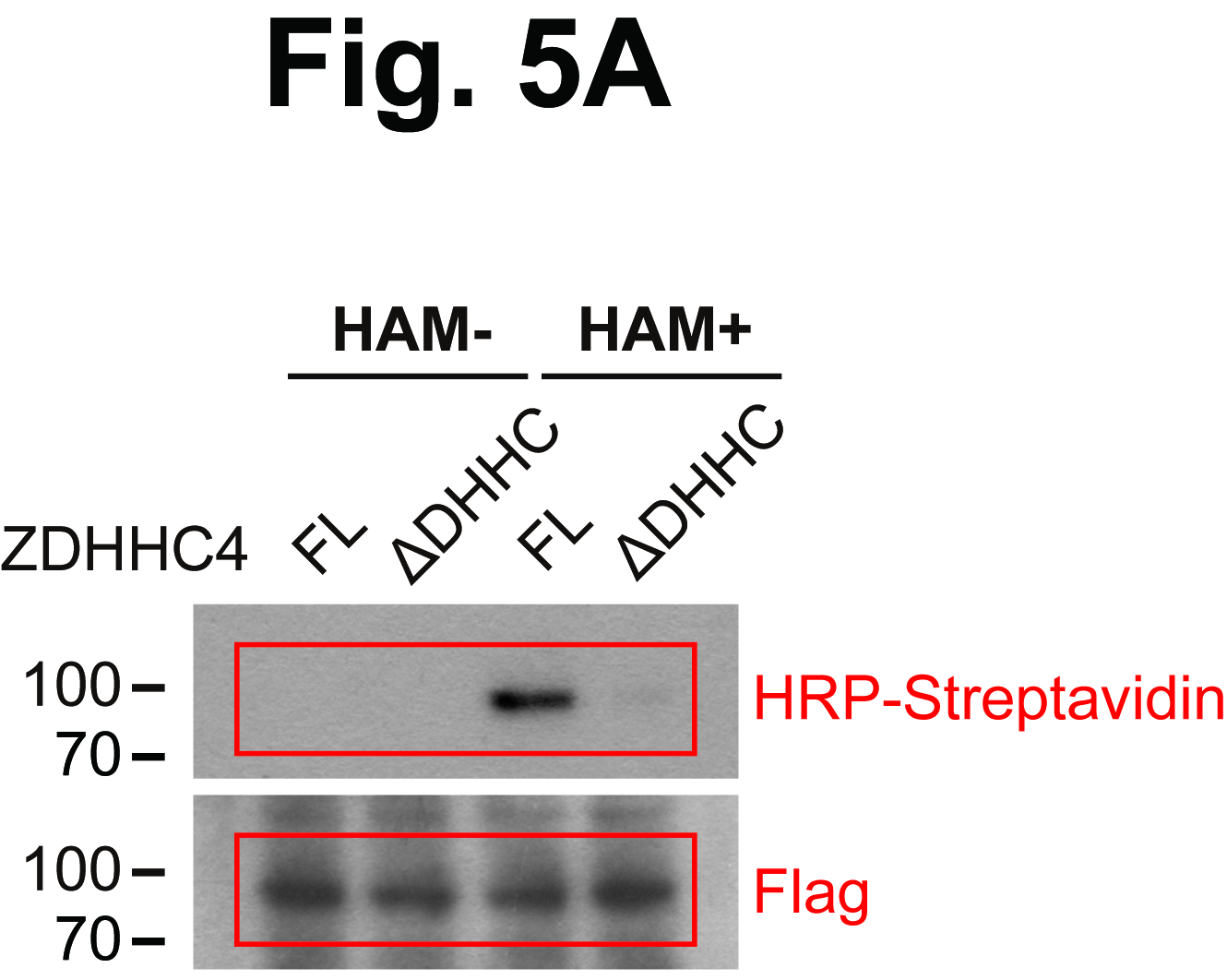

Supplement: Supplementary file 7 — Source data Fig. 5 [file 44319_2024_317_MOESM7_ESM.zip › 5A-data source.tif]

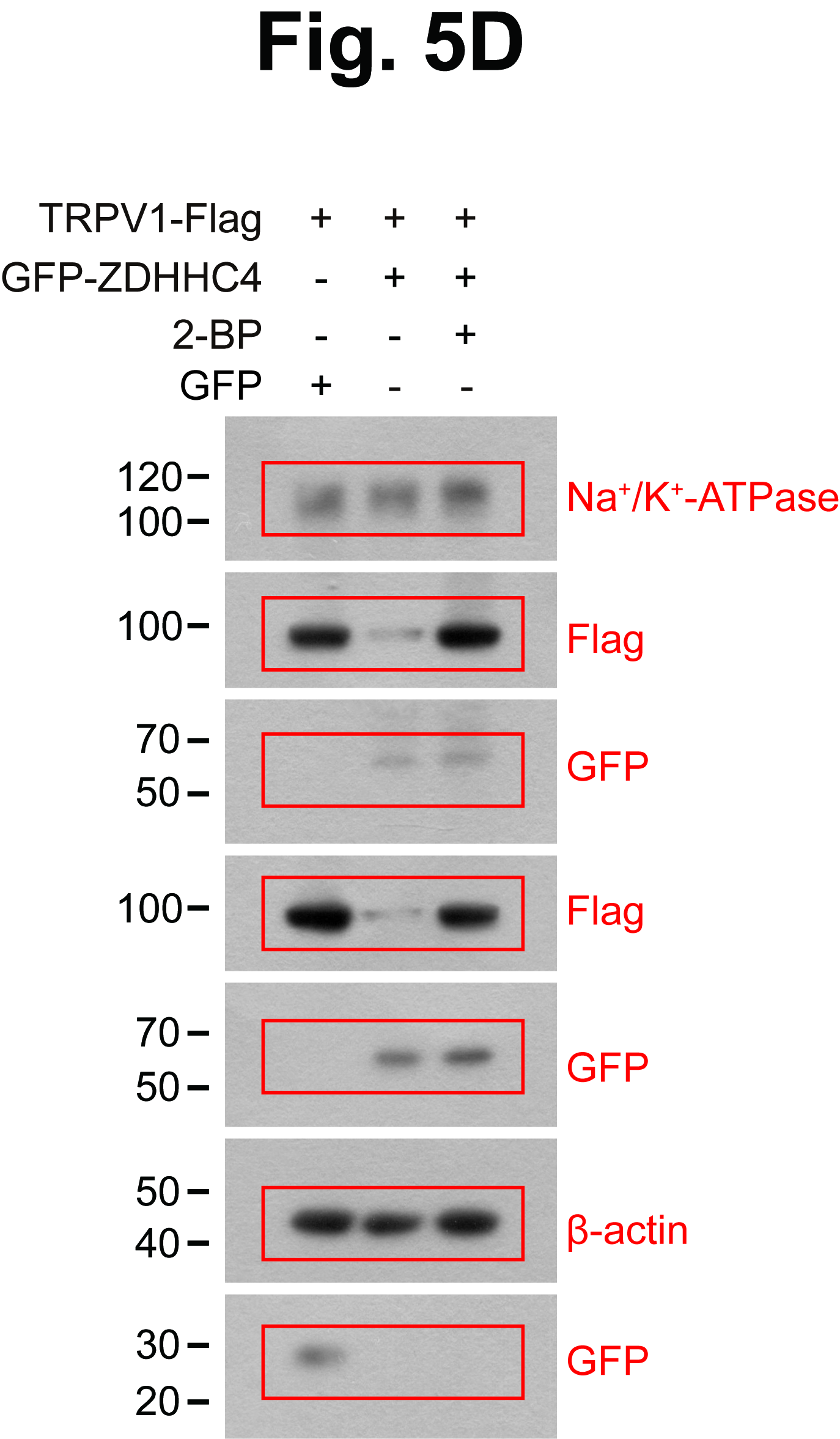

Supplement: Supplementary file 7 — Source data Fig. 5 [file 44319_2024_317_MOESM7_ESM.zip › 5D/Figure 5D.tif]

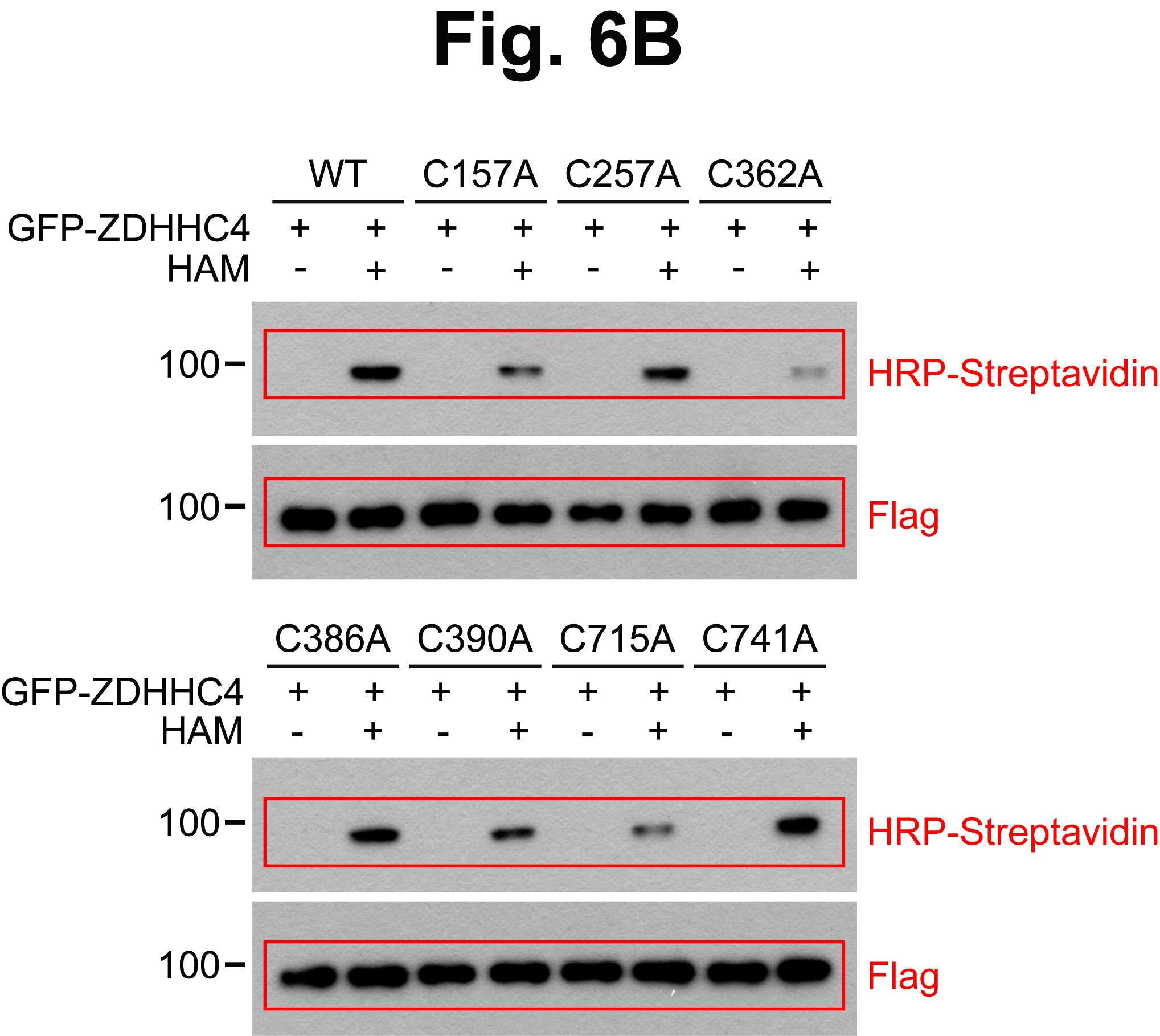

Supplement: Supplementary file 8 — Source data Fig. 6 [file 44319_2024_317_MOESM8_ESM.zip › 6B_data source.tif]

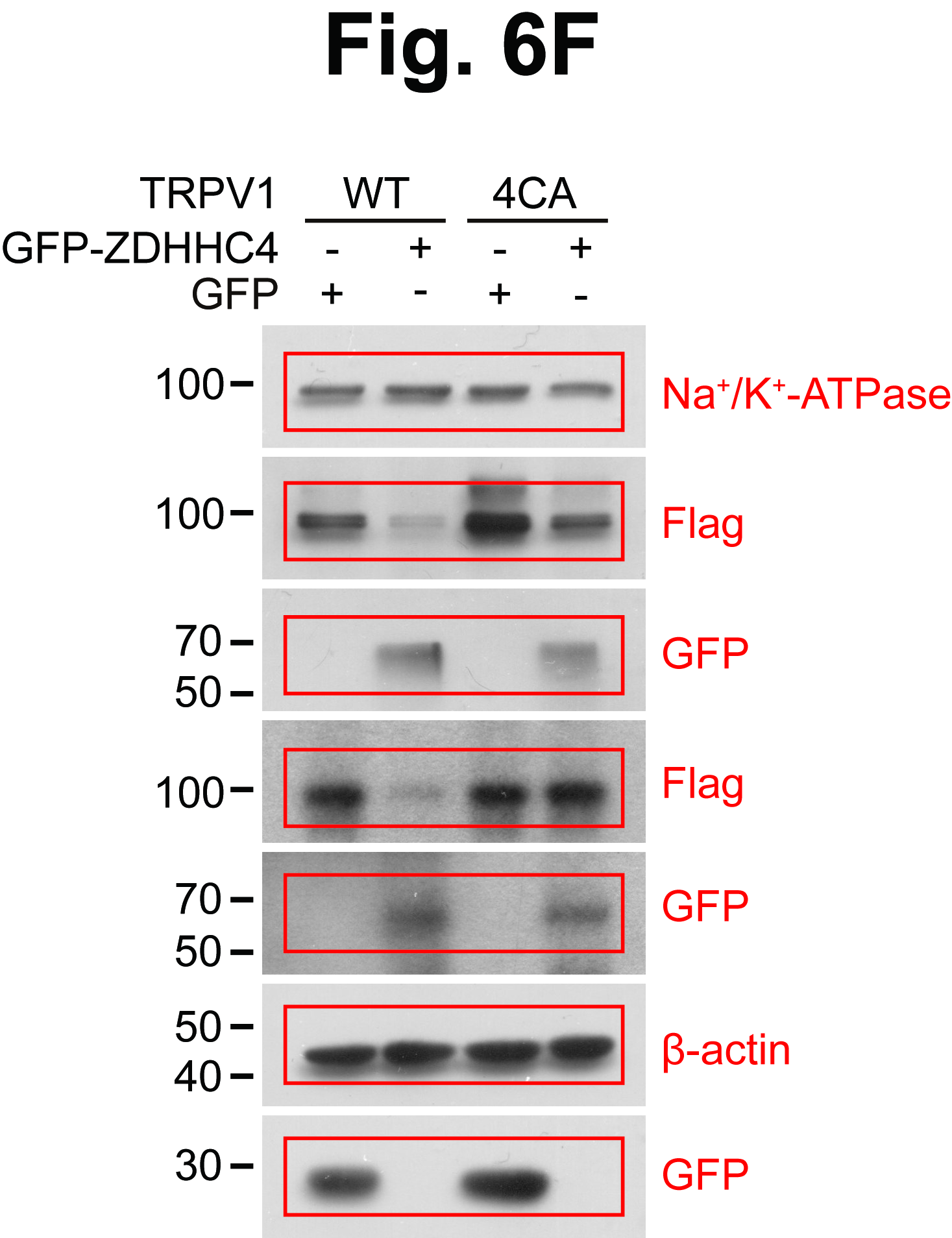

Supplement: Supplementary file 8 — Source data Fig. 6 [file 44319_2024_317_MOESM8_ESM.zip › 6F_data source.tif]

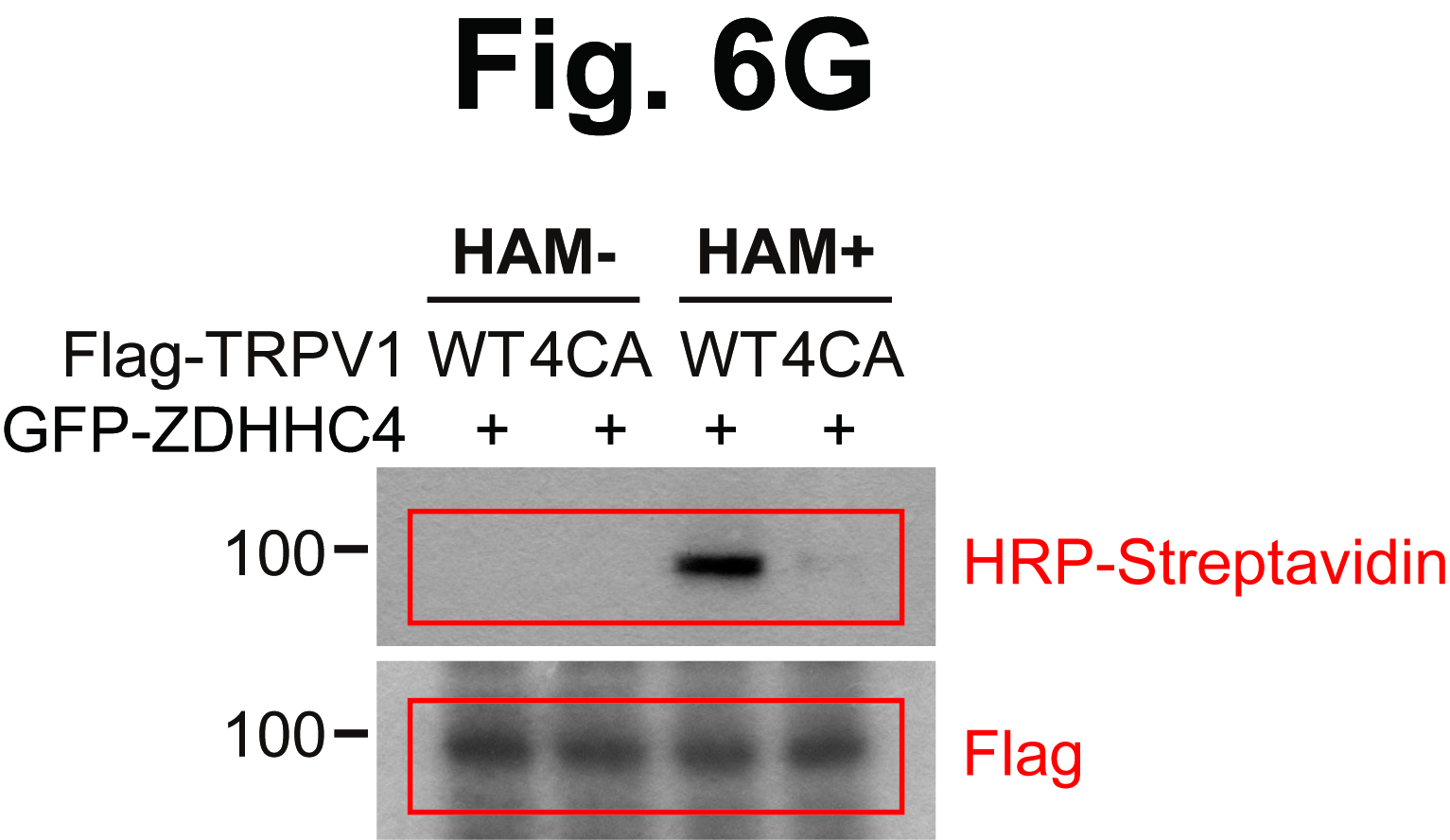

Supplement: Supplementary file 8 — Source data Fig. 6 [file 44319_2024_317_MOESM8_ESM.zip › 6H-data source.tif]

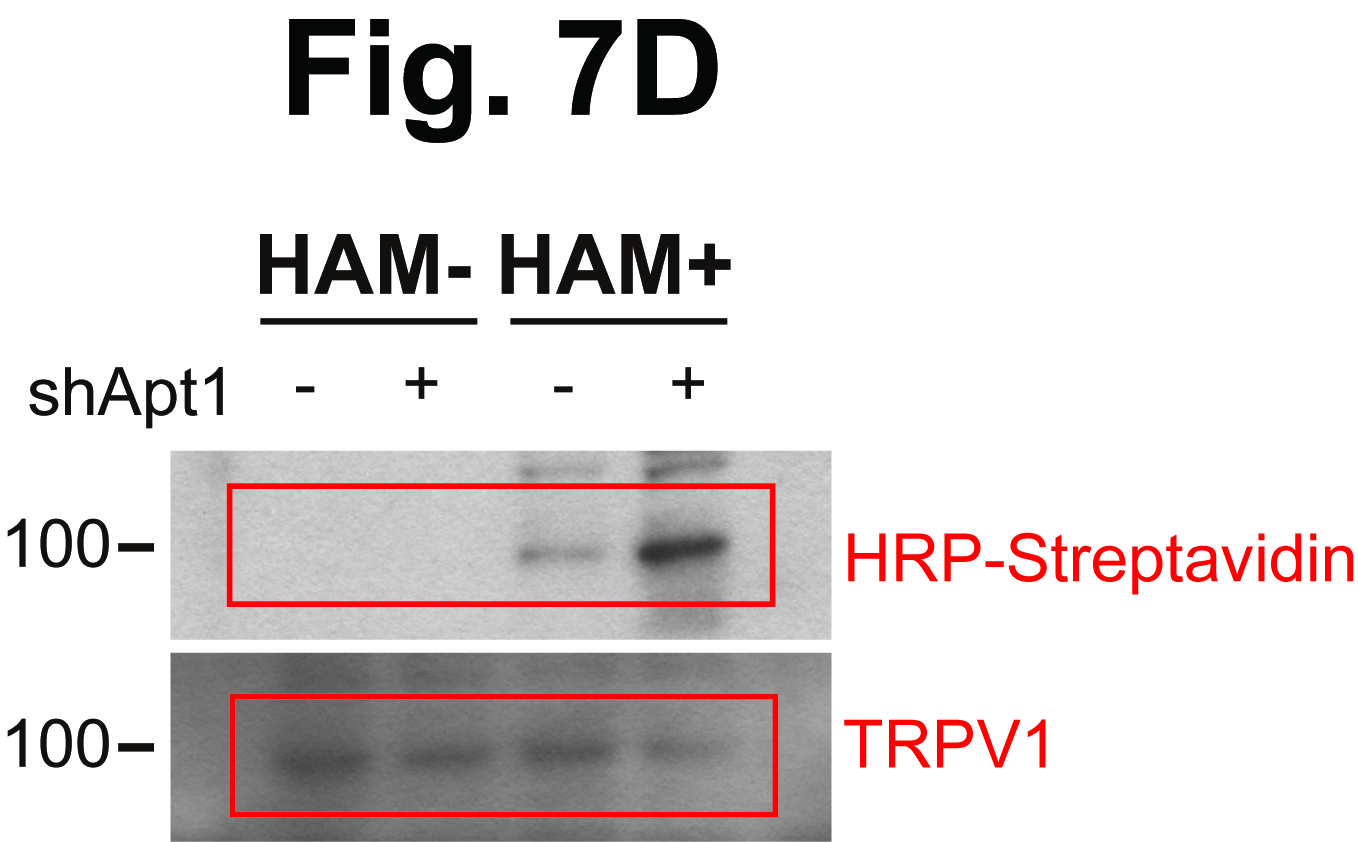

Supplement: Supplementary file 9 — Source data Fig. 7 [file 44319_2024_317_MOESM9_ESM.zip › 7D_data source.tif]

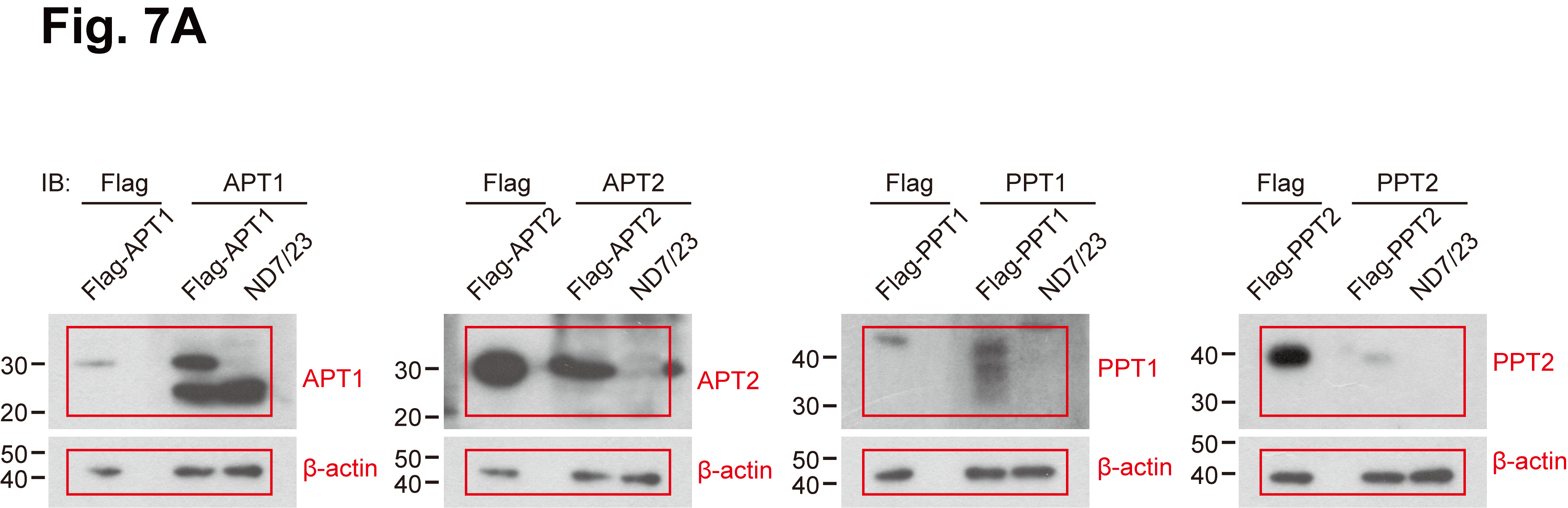

Supplement: Supplementary file 9 — Source data Fig. 7 [file 44319_2024_317_MOESM9_ESM.zip › 7A_data source.tif]

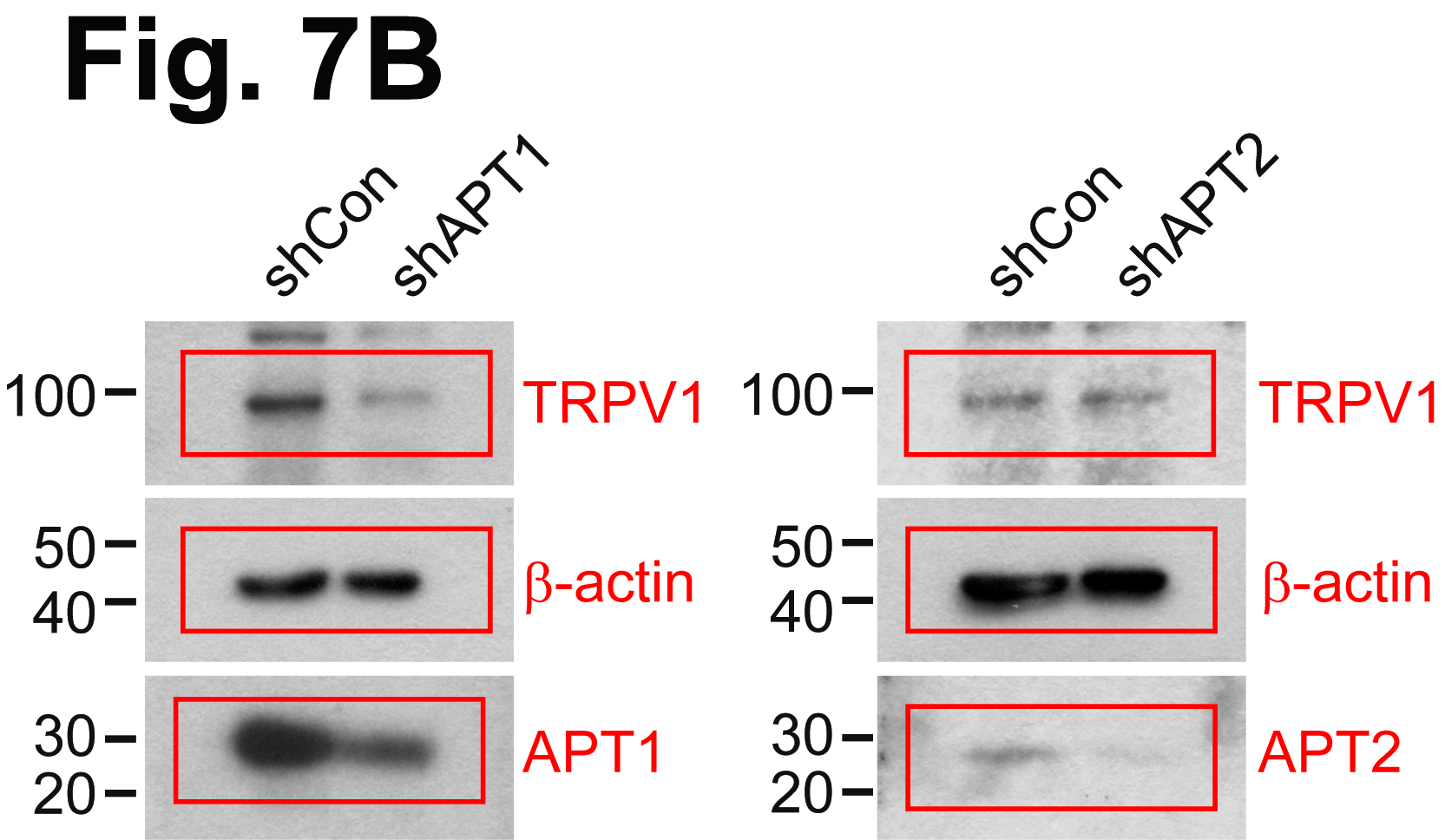

Supplement: Supplementary file 9 — Source data Fig. 7 [file 44319_2024_317_MOESM9_ESM.zip › 7B_data source.tif]

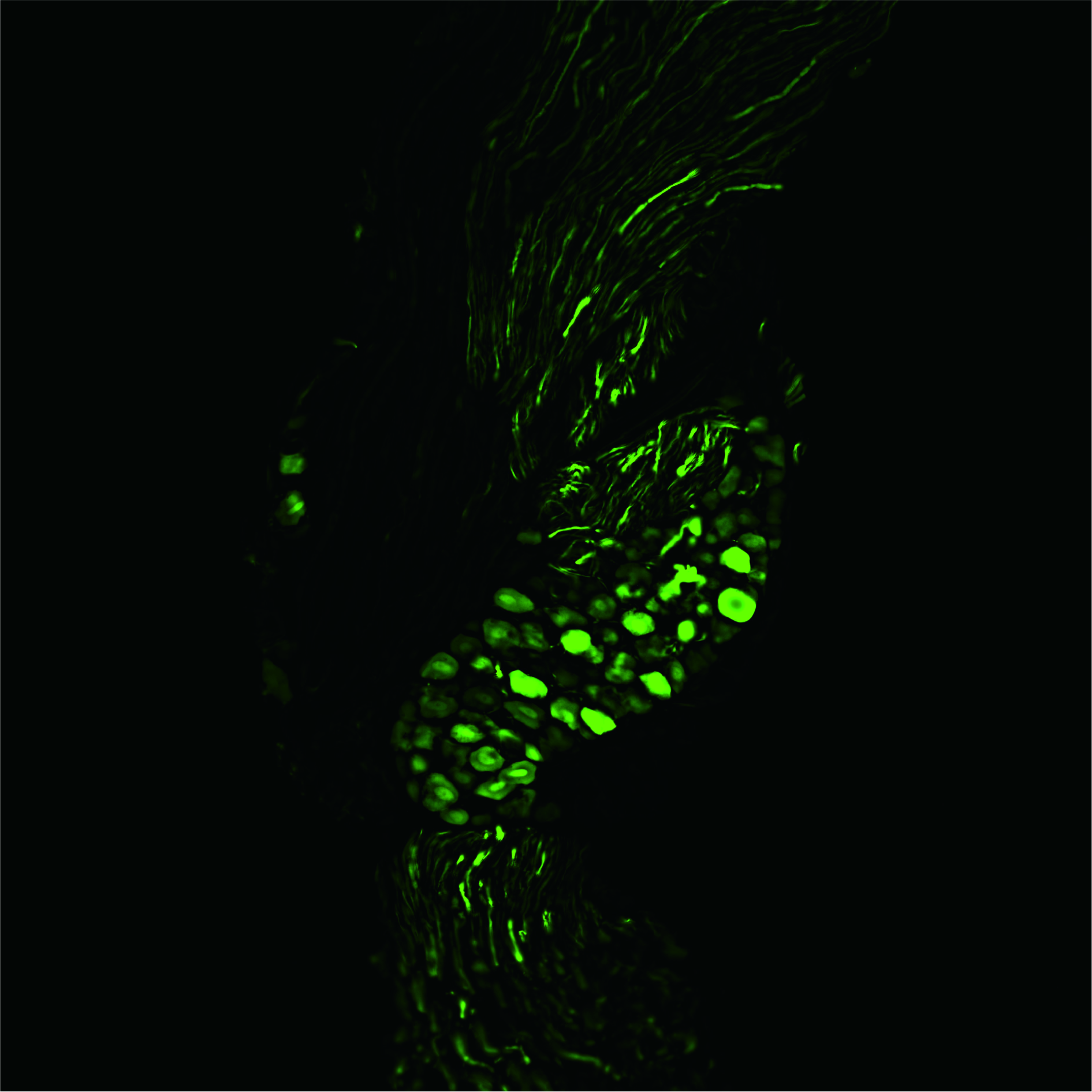

Supplement: Supplementary file 10 — Source data Fig. 8 [file 44319_2024_317_MOESM10_ESM.zip › 8A/8A_V1KO-shAPT1.tif]

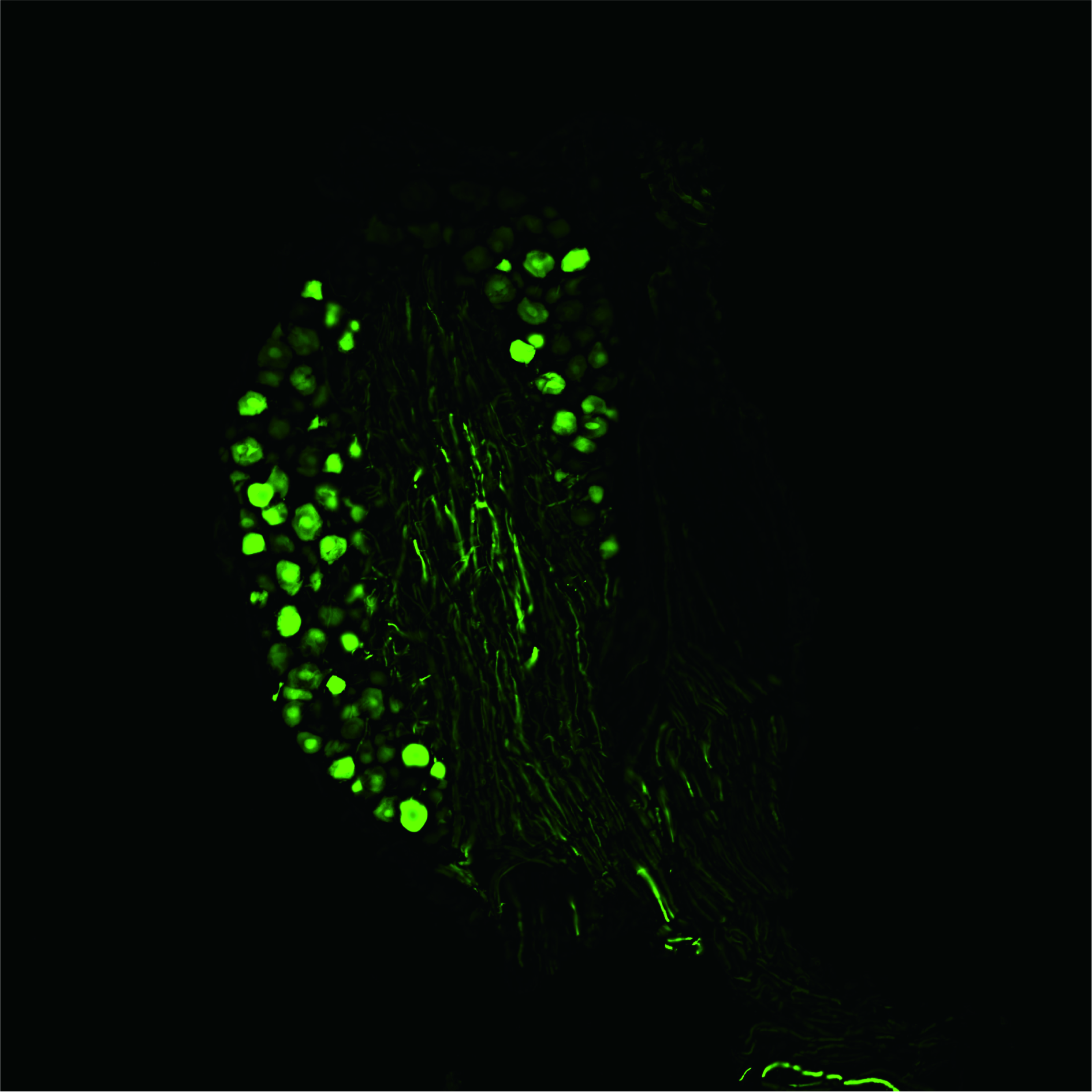

Supplement: Supplementary file 10 — Source data Fig. 8 [file 44319_2024_317_MOESM10_ESM.zip › 8A/8A_V1KO-shScramble.tif]

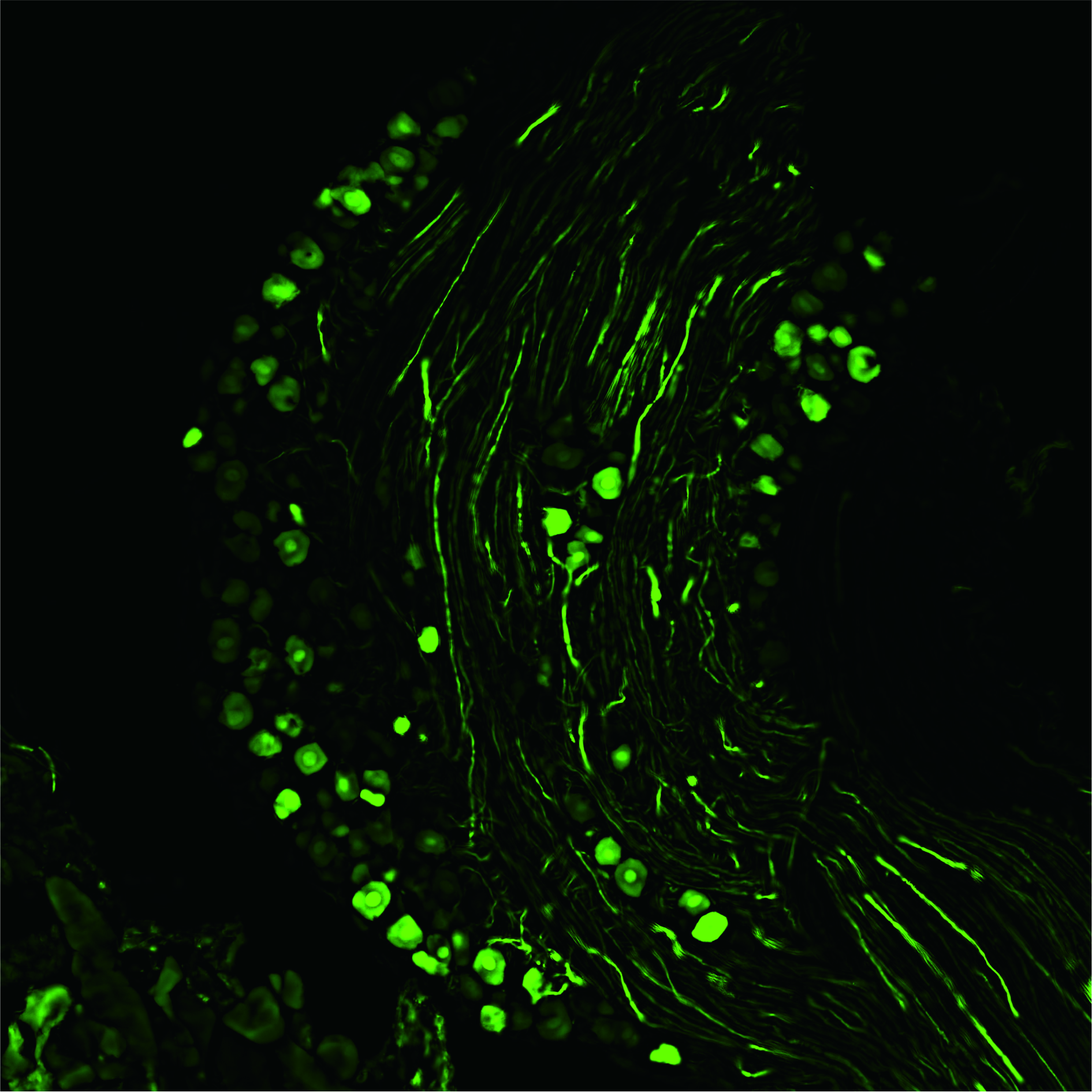

Supplement: Supplementary file 10 — Source data Fig. 8 [file 44319_2024_317_MOESM10_ESM.zip › 8A/8A_V1KO-shZDHHC4.tif]

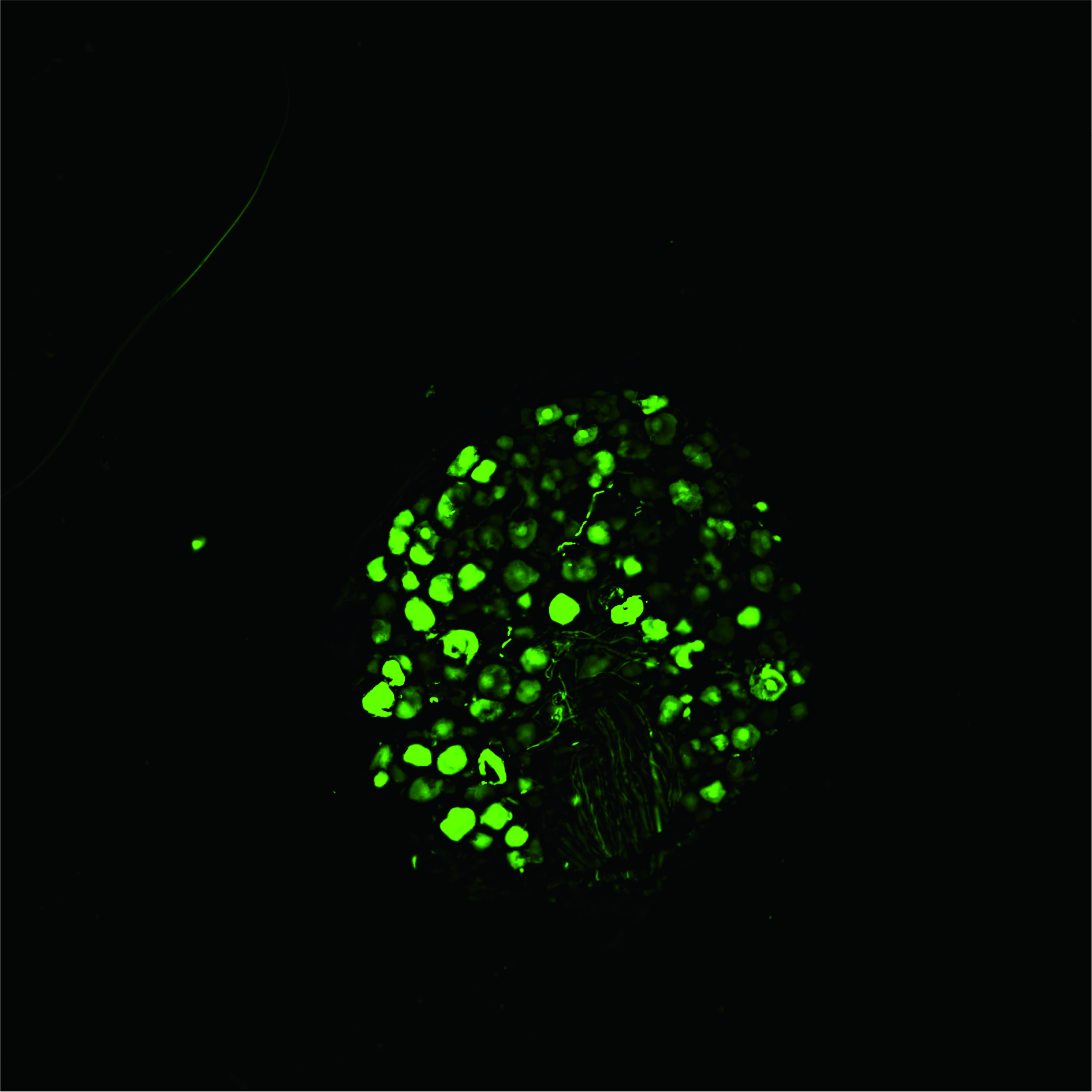

Supplement: Supplementary file 10 — Source data Fig. 8 [file 44319_2024_317_MOESM10_ESM.zip › 8A/8A_WT-shAPT1.tif]

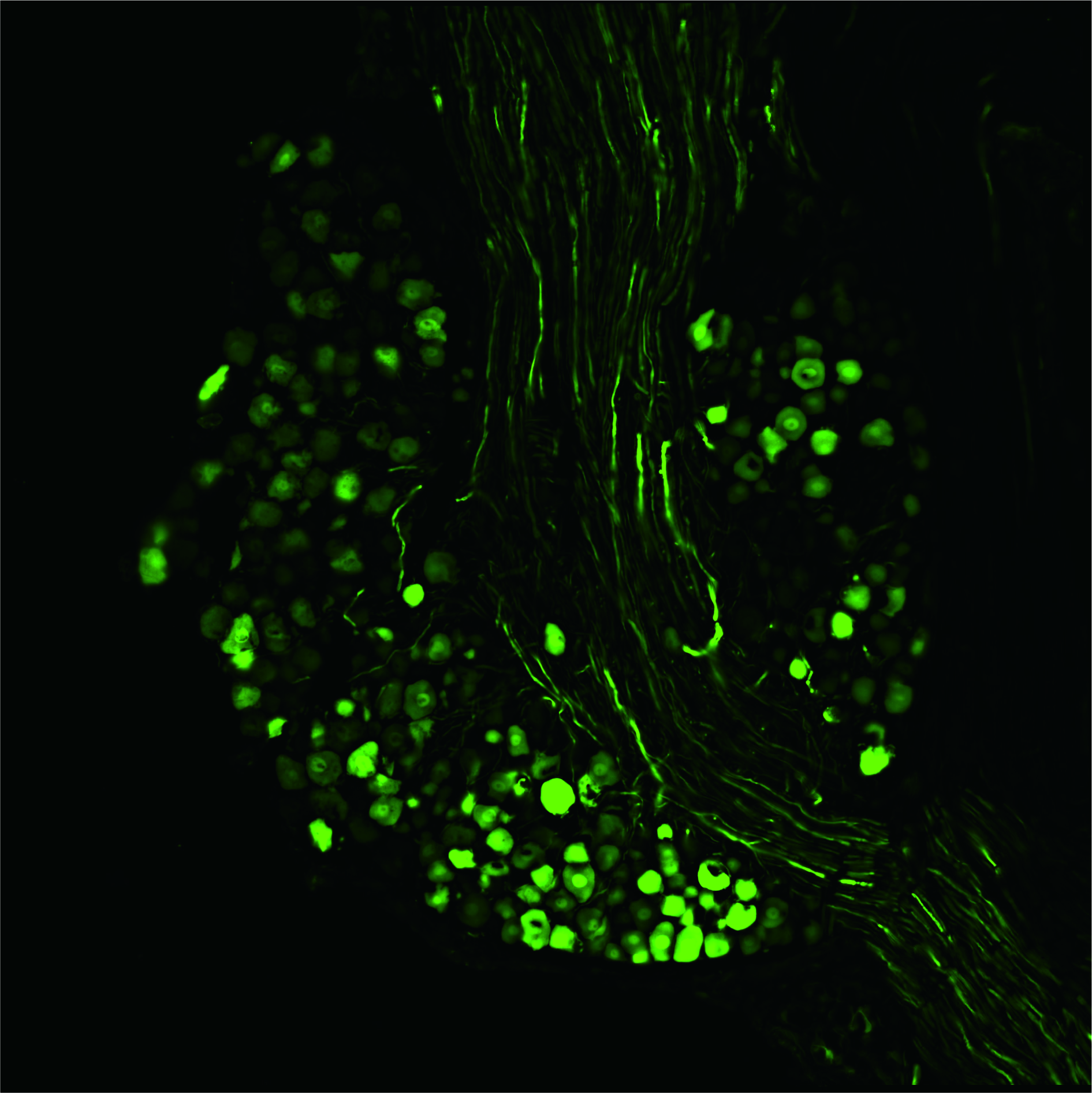

Supplement: Supplementary file 10 — Source data Fig. 8 [file 44319_2024_317_MOESM10_ESM.zip › 8A/8A_WT-shScramble.tif]

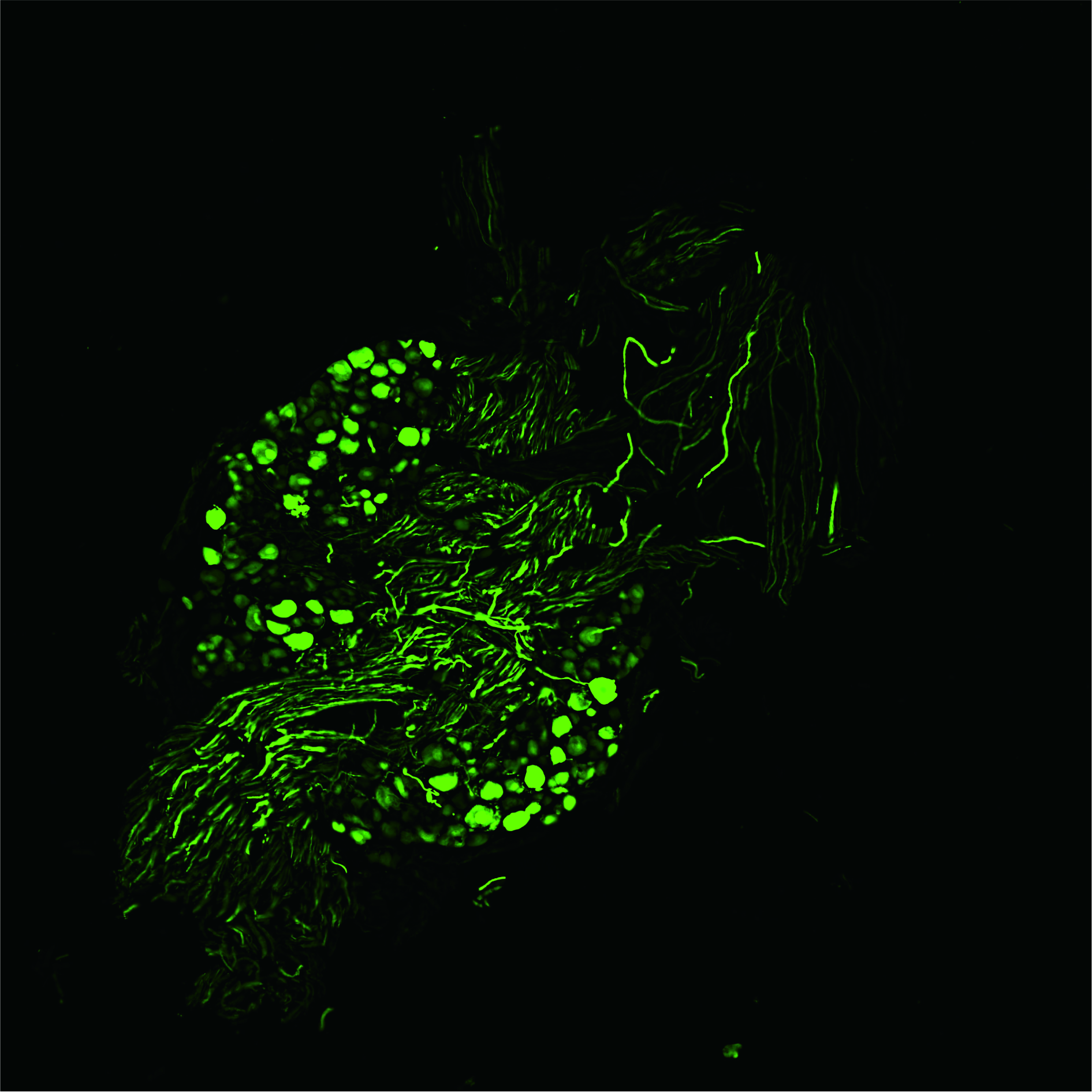

Supplement: Supplementary file 10 — Source data Fig. 8 [file 44319_2024_317_MOESM10_ESM.zip › 8A/8A_WT-shZDHHC4.tif]

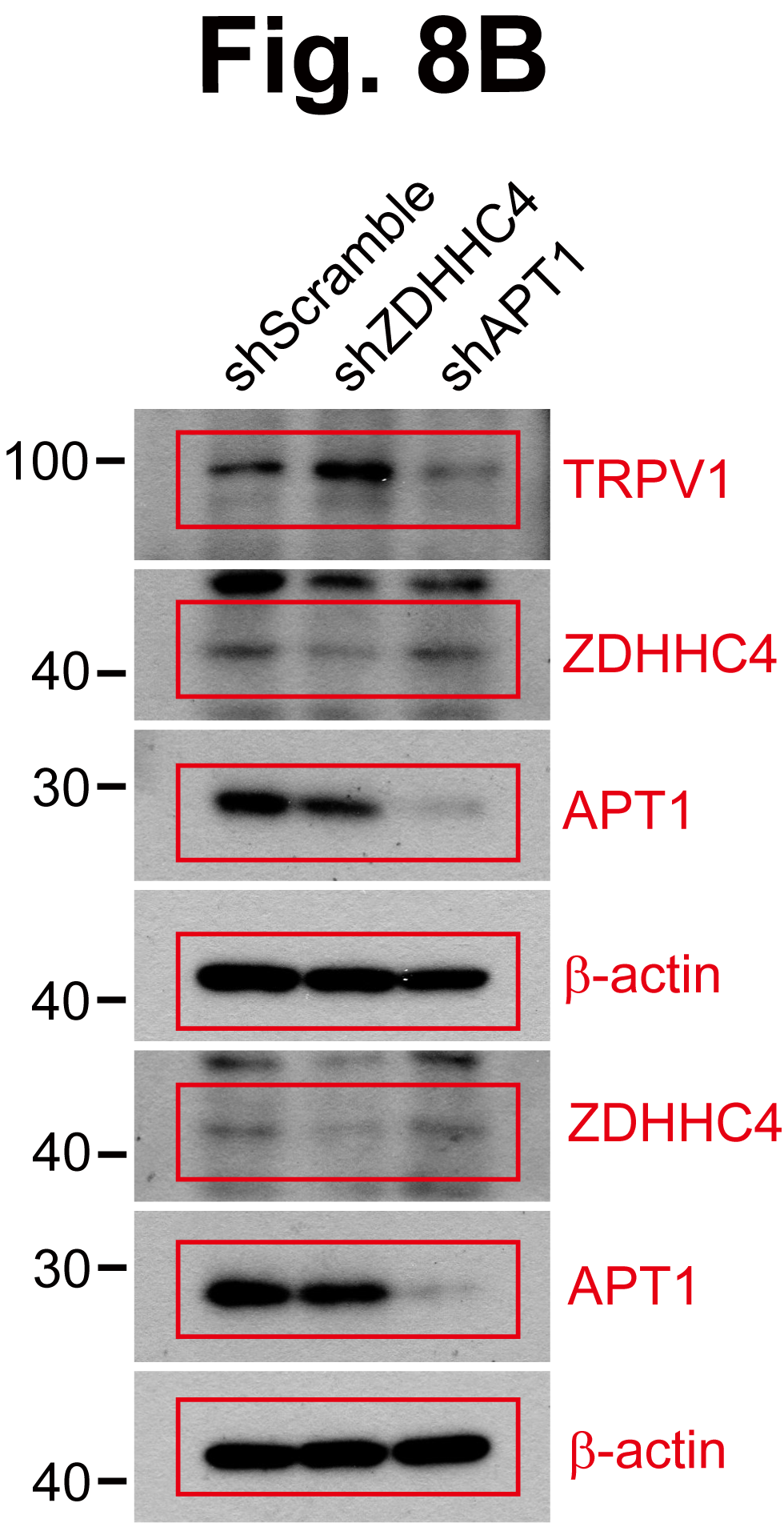

Supplement: Supplementary file 10 — Source data Fig. 8 [file 44319_2024_317_MOESM10_ESM.zip › 8B-data source.tif]

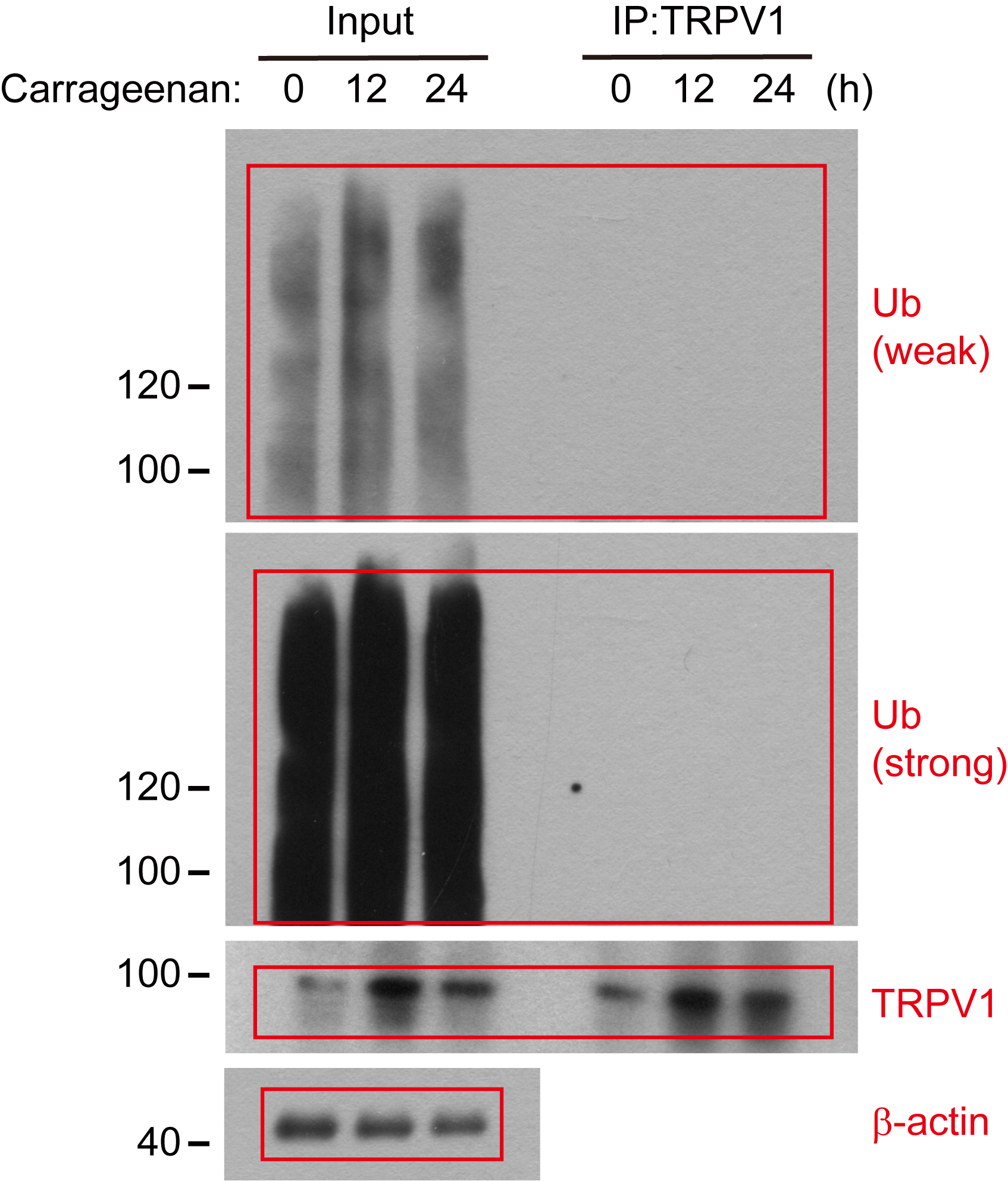

Supplement: Supplementary file 11 — Appendix Figures Source Data [file 44319_2024_317_MOESM11_ESM.zip › Appendix Source Data/Appendix Figure S1/Appendix Figure S1_Data Source.tif]

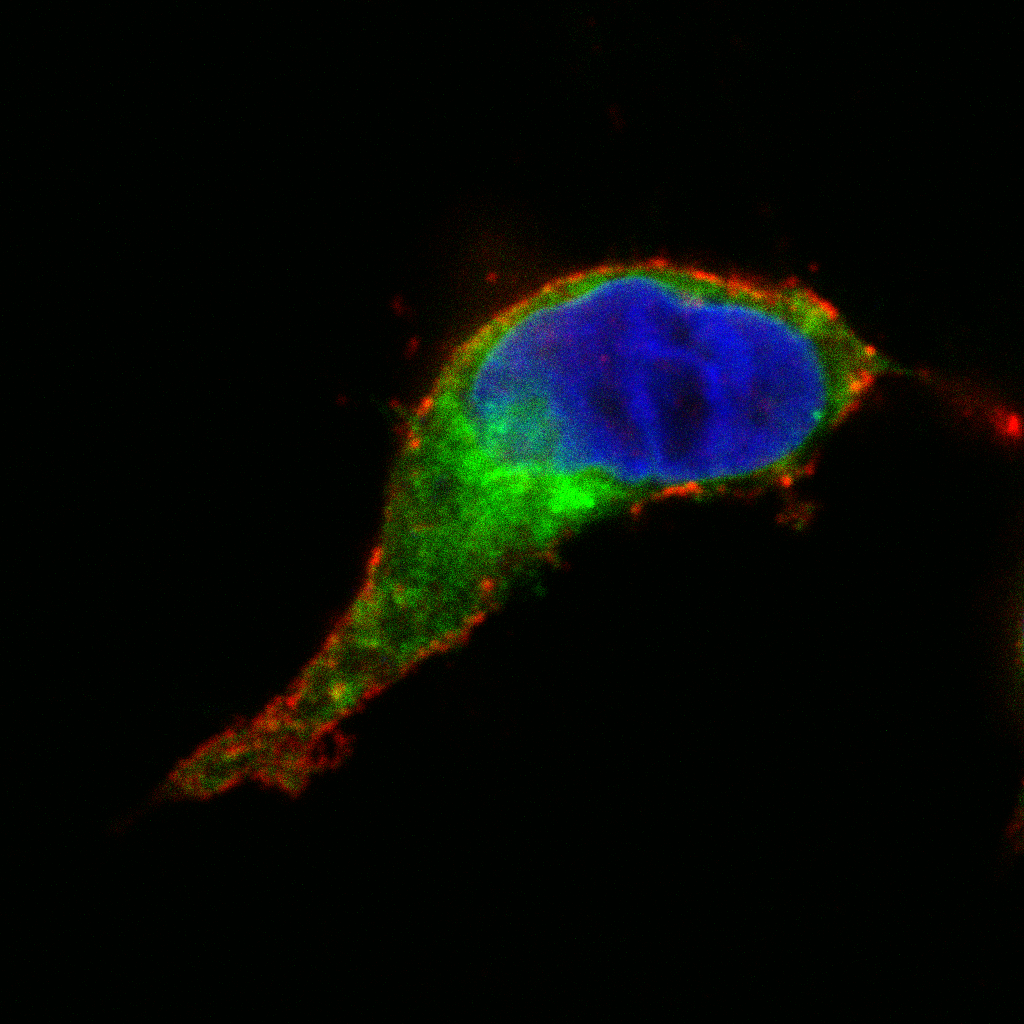

Supplement: Supplementary file 11 — Appendix Figures Source Data [file 44319_2024_317_MOESM11_ESM.zip › Appendix Source Data/Appendix Figure S2/Appendix Figure S2A/Merge.tif]

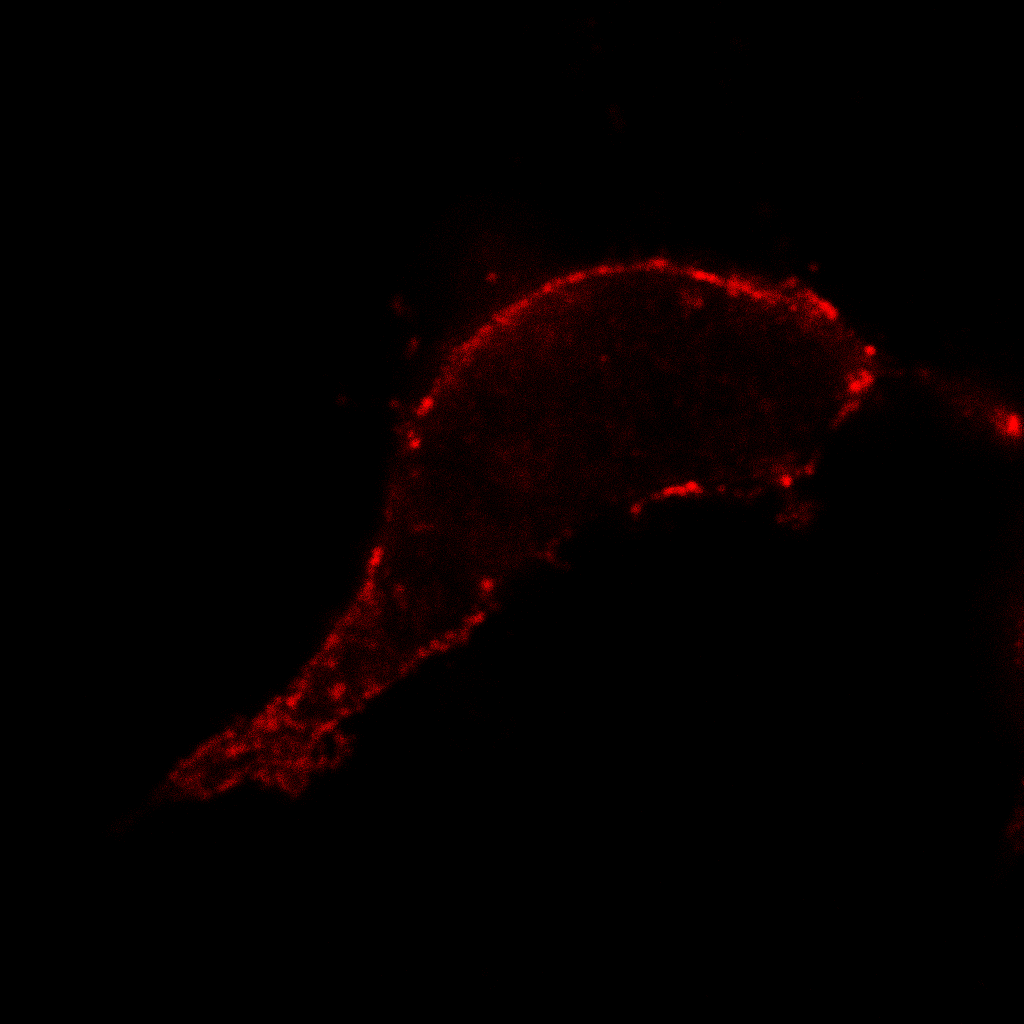

Supplement: Supplementary file 11 — Appendix Figures Source Data [file 44319_2024_317_MOESM11_ESM.zip › Appendix Source Data/Appendix Figure S2/Appendix Figure S2A/TRPV1-mcherry.tif]

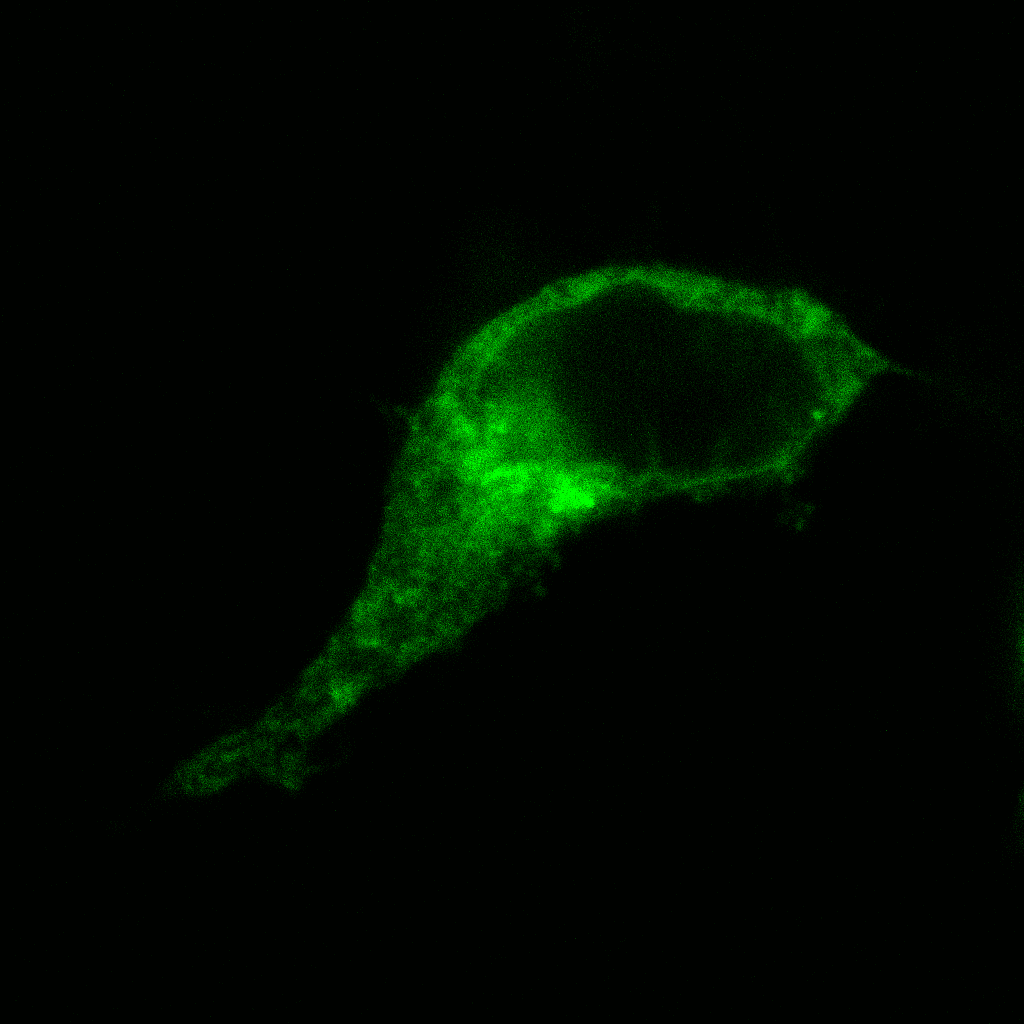

Supplement: Supplementary file 11 — Appendix Figures Source Data [file 44319_2024_317_MOESM11_ESM.zip › Appendix Source Data/Appendix Figure S2/Appendix Figure S2A/ZDHHC4-GFP.tif]

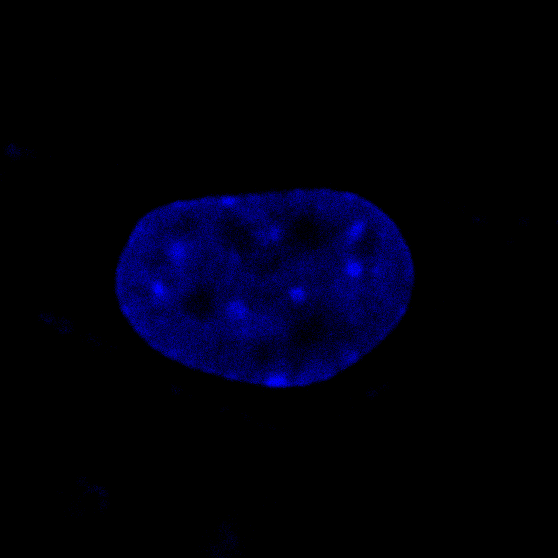

Supplement: Supplementary file 11 — Appendix Figures Source Data [file 44319_2024_317_MOESM11_ESM.zip › Appendix Source Data/Appendix Figure S2/Appendix Figure S2B/GFP-Nsf+TRPV1-Csf.tif]

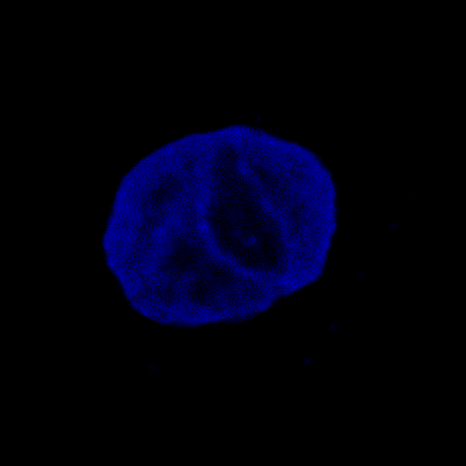

Supplement: Supplementary file 11 — Appendix Figures Source Data [file 44319_2024_317_MOESM11_ESM.zip › Appendix Source Data/Appendix Figure S2/Appendix Figure S2B/ZDHHC4-Nsf+GFP-Csf.tif]

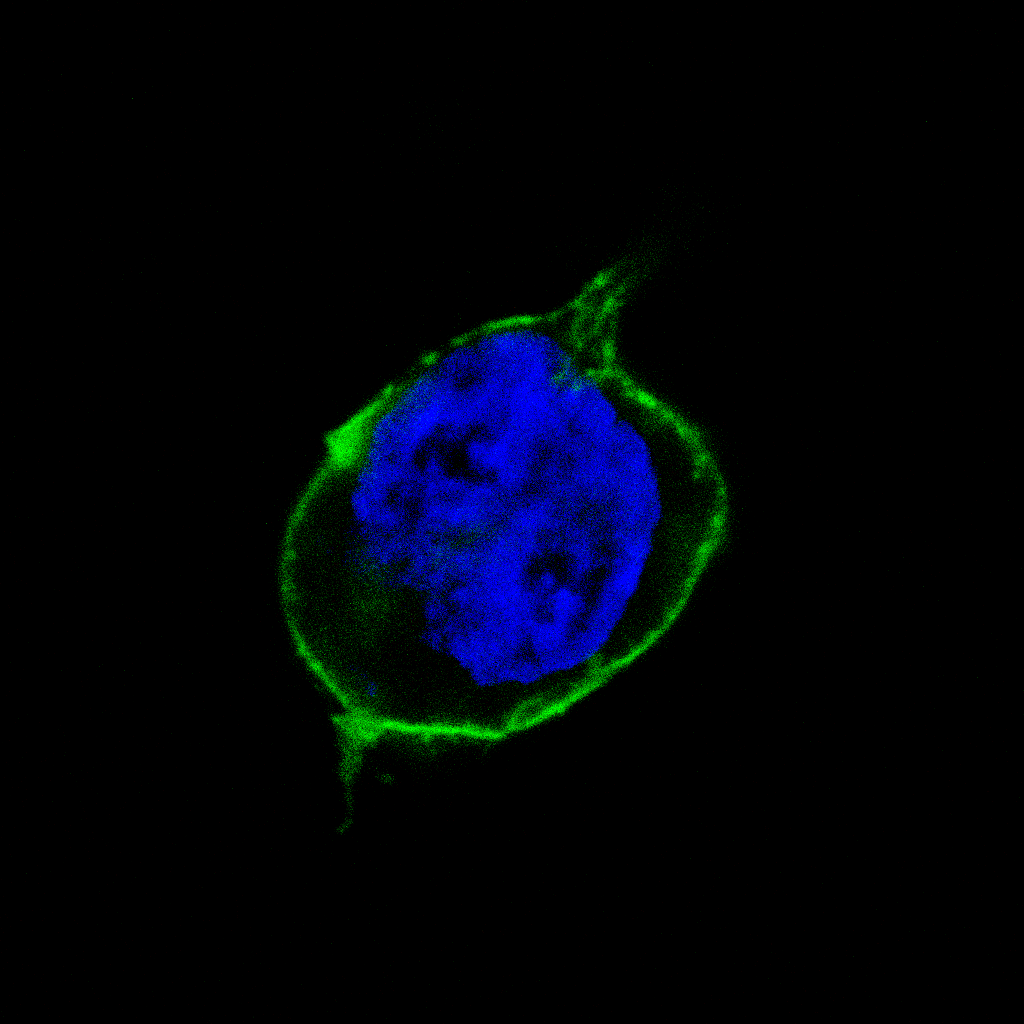

Supplement: Supplementary file 11 — Appendix Figures Source Data [file 44319_2024_317_MOESM11_ESM.zip › Appendix Source Data/Appendix Figure S2/Appendix Figure S2B/ZDHHC4-Nsf+TRPV1-Csf.tif]

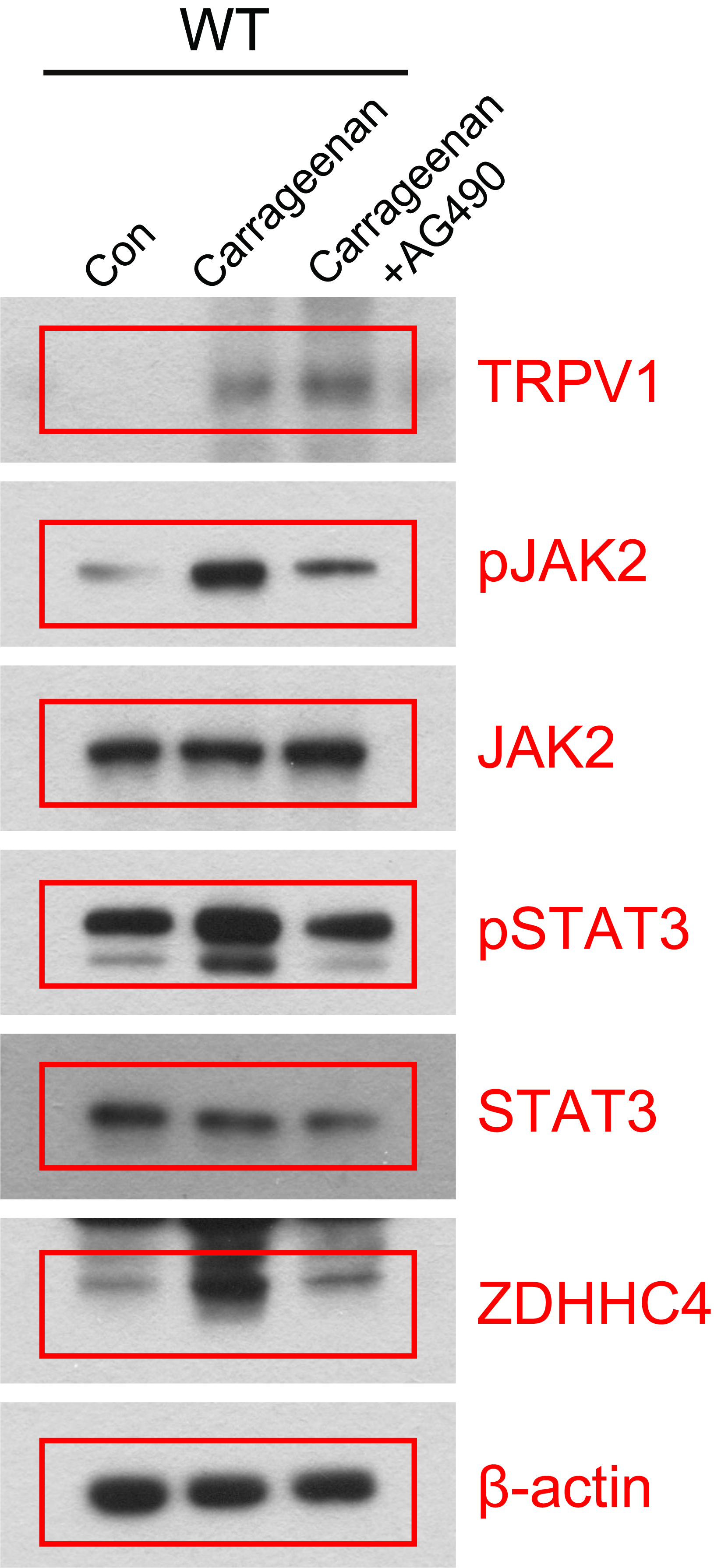

Supplement: Supplementary file 11 — Appendix Figures Source Data [file 44319_2024_317_MOESM11_ESM.zip › Appendix Source Data/Appendix Figure S2/Appendix Figure S2C-D/Figure S2C_data source.tif]

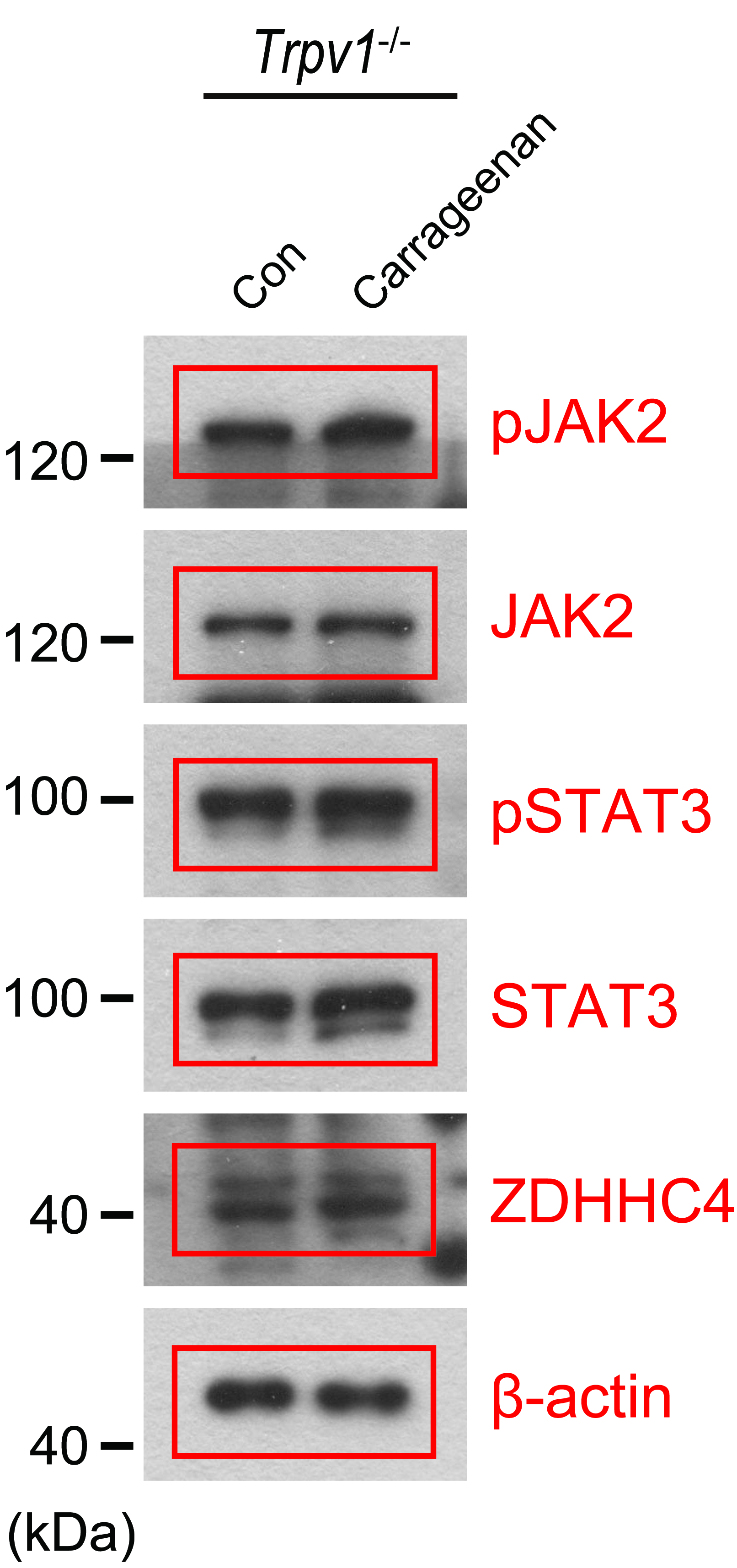

Supplement: Supplementary file 11 — Appendix Figures Source Data [file 44319_2024_317_MOESM11_ESM.zip › Appendix Source Data/Appendix Figure S2/Appendix Figure S2C-D/Figure S2D_data source.tif]

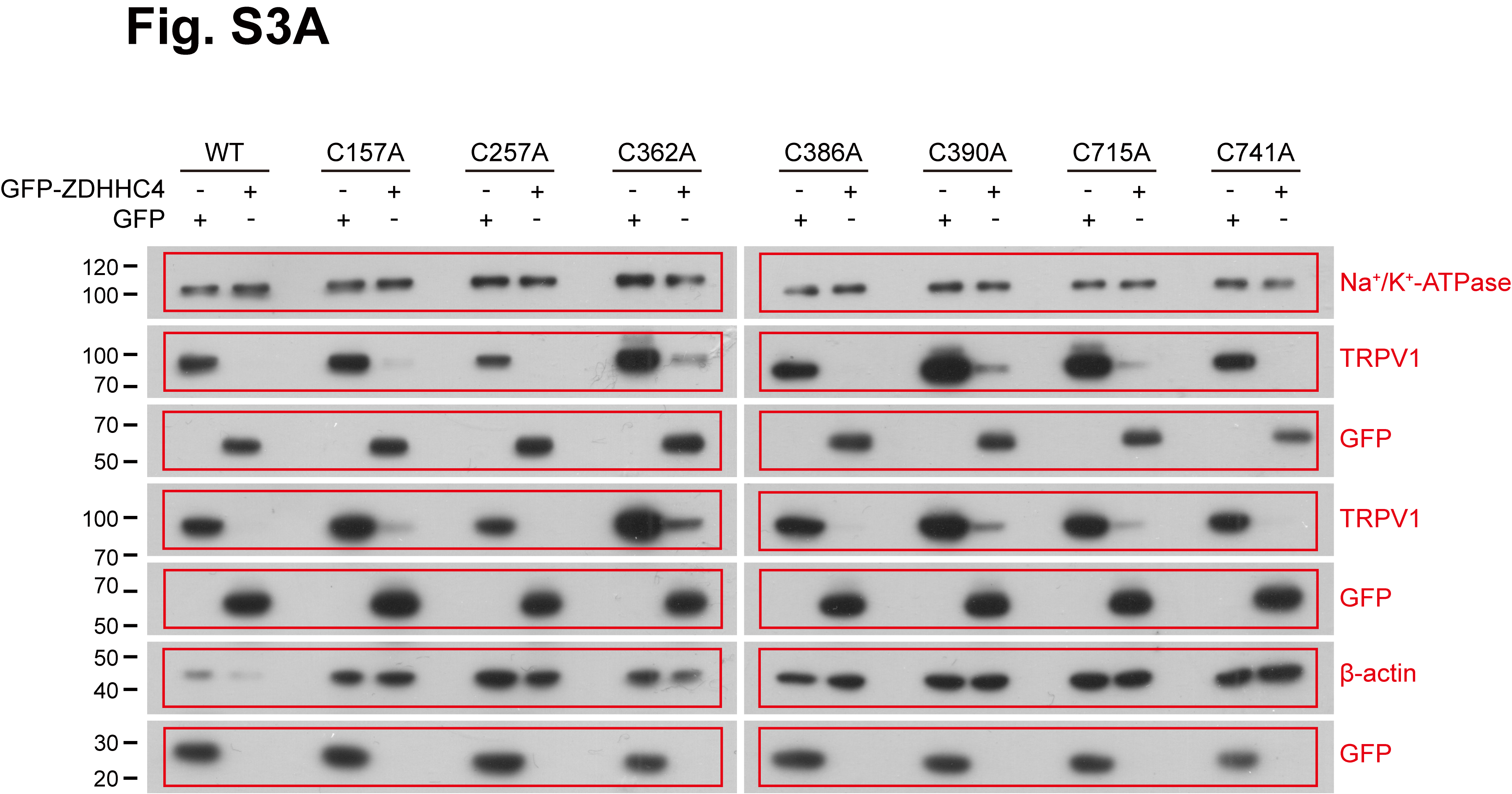

Supplement: Supplementary file 11 — Appendix Figures Source Data [file 44319_2024_317_MOESM11_ESM.zip › Appendix Source Data/Appendix Figure S3/Appendix Figure S3A.tif]

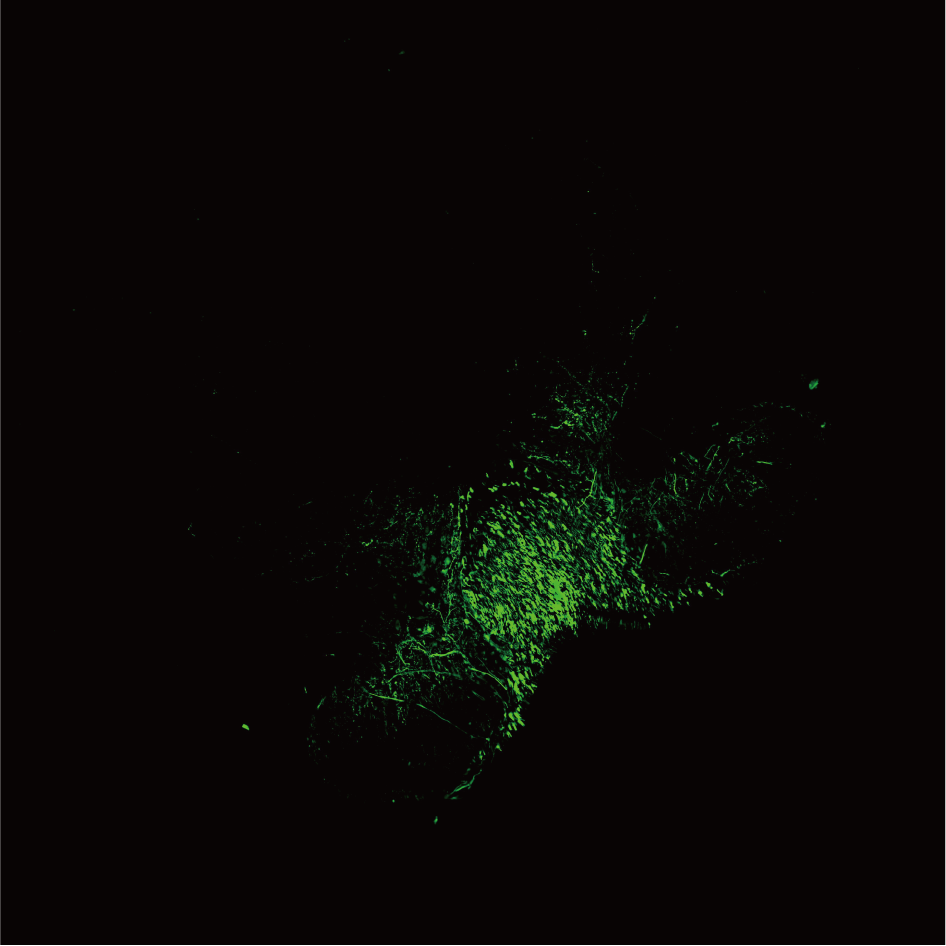

Supplement: Supplementary file 11 — Appendix Figures Source Data [file 44319_2024_317_MOESM11_ESM.zip › Appendix Source Data/Appendix Figure S4/Appendix Figure S4A/KO_shAPT1.tif]

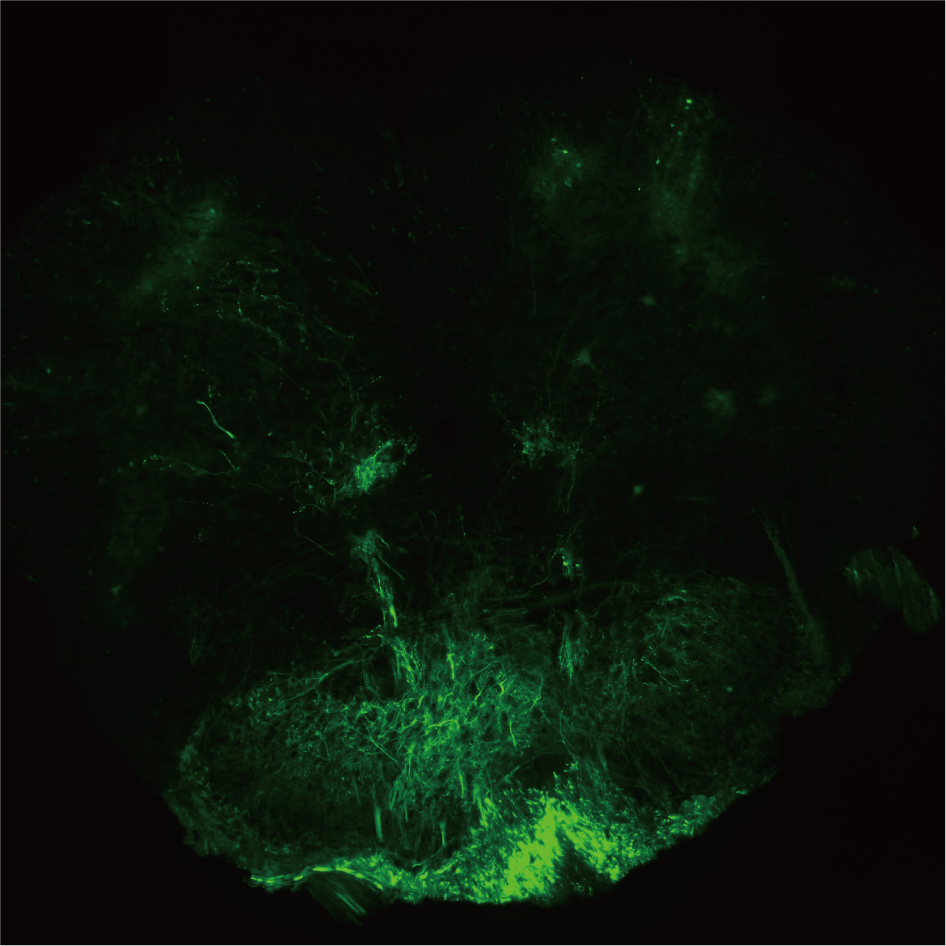

Supplement: Supplementary file 11 — Appendix Figures Source Data [file 44319_2024_317_MOESM11_ESM.zip › Appendix Source Data/Appendix Figure S4/Appendix Figure S4A/KO_shZDHHC4.tif]

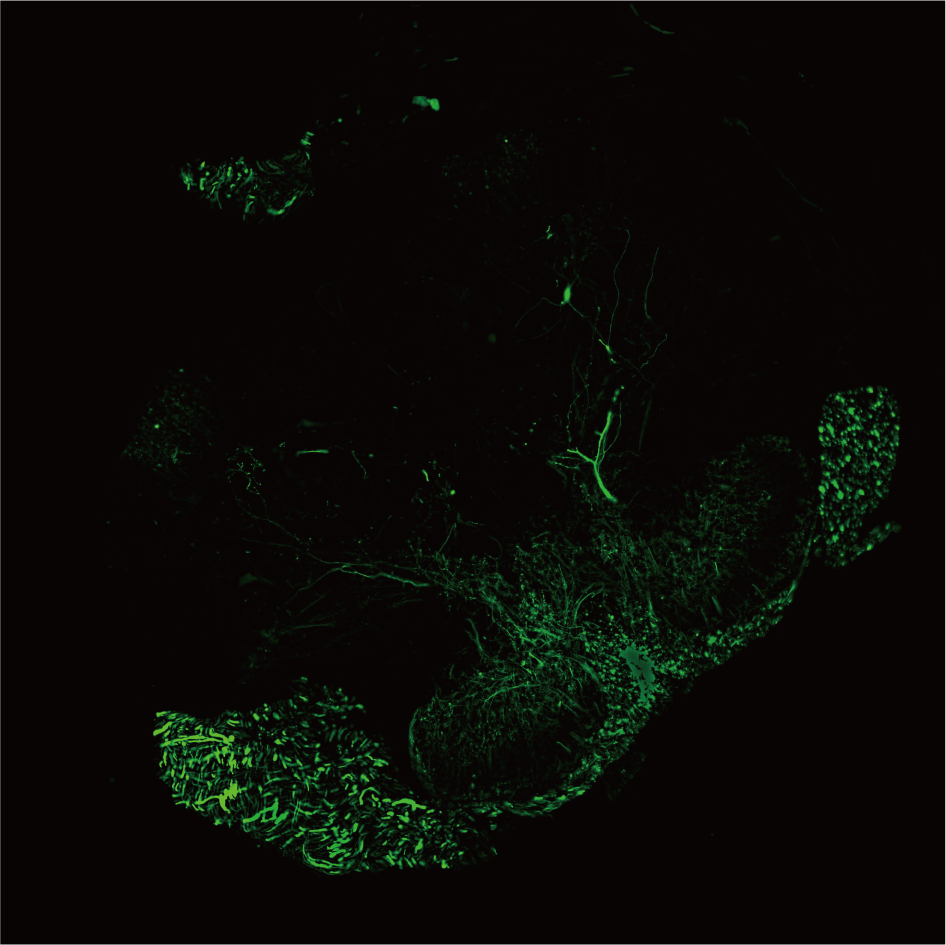

Supplement: Supplementary file 11 — Appendix Figures Source Data [file 44319_2024_317_MOESM11_ESM.zip › Appendix Source Data/Appendix Figure S4/Appendix Figure S4A/V1KO_shScramble.tif]

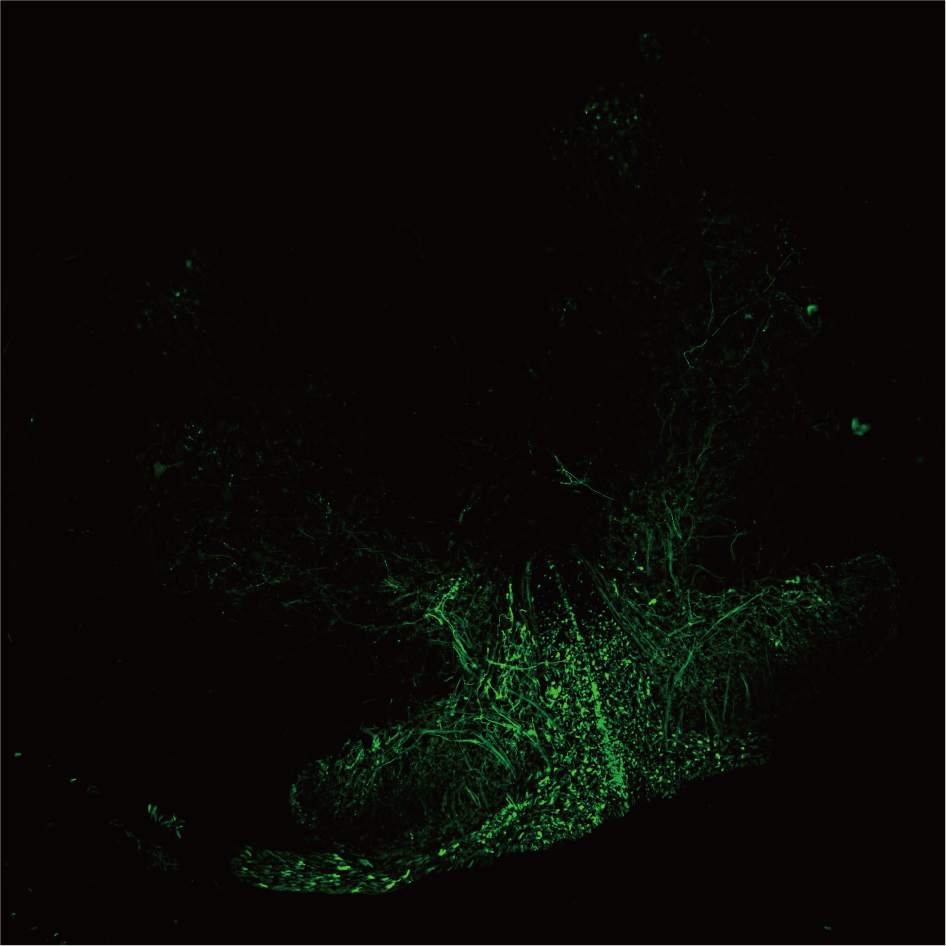

Supplement: Supplementary file 11 — Appendix Figures Source Data [file 44319_2024_317_MOESM11_ESM.zip › Appendix Source Data/Appendix Figure S4/Appendix Figure S4A/WT_shAPT1.tif]

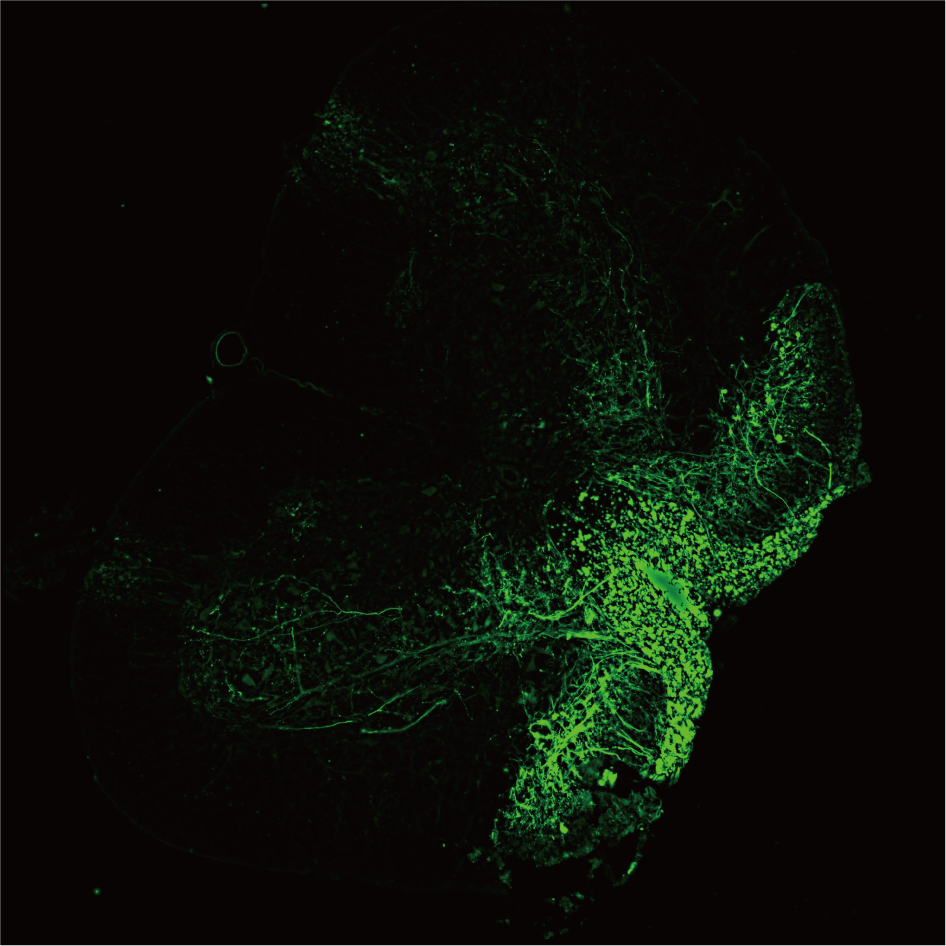

Supplement: Supplementary file 11 — Appendix Figures Source Data [file 44319_2024_317_MOESM11_ESM.zip › Appendix Source Data/Appendix Figure S4/Appendix Figure S4A/WT_shScramble.tif]

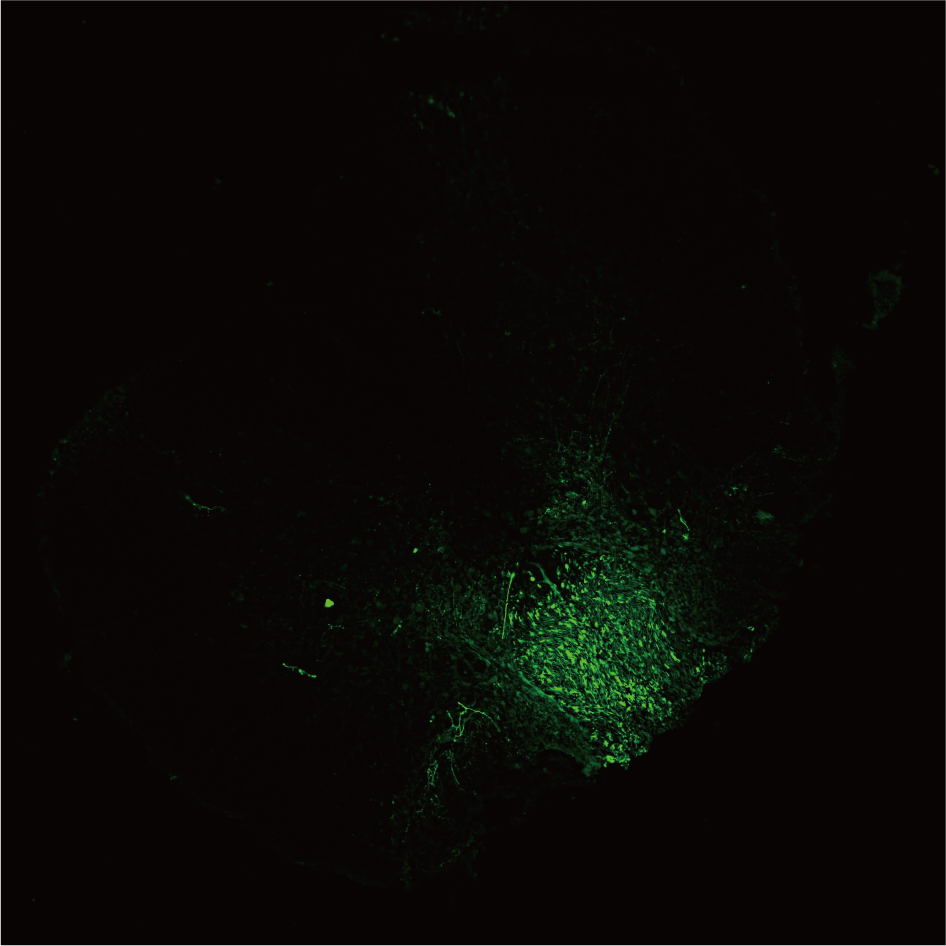

Supplement: Supplementary file 11 — Appendix Figures Source Data [file 44319_2024_317_MOESM11_ESM.zip › Appendix Source Data/Appendix Figure S4/Appendix Figure S4A/WT_shZDHHC4.tif]

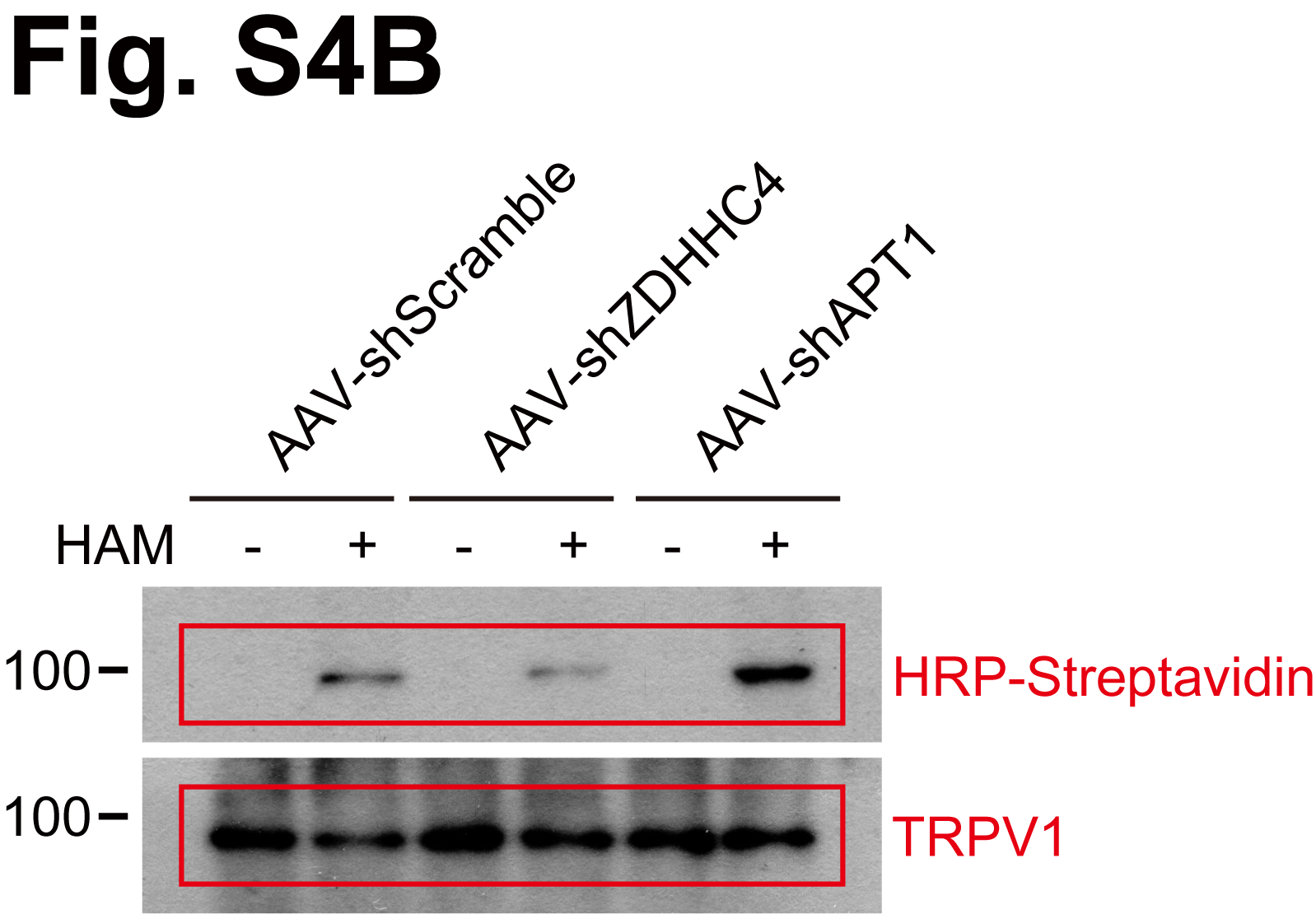

Supplement: Supplementary file 11 — Appendix Figures Source Data [file 44319_2024_317_MOESM11_ESM.zip › Appendix Source Data/Appendix Figure S4/Appendix Figure S4B.tif]

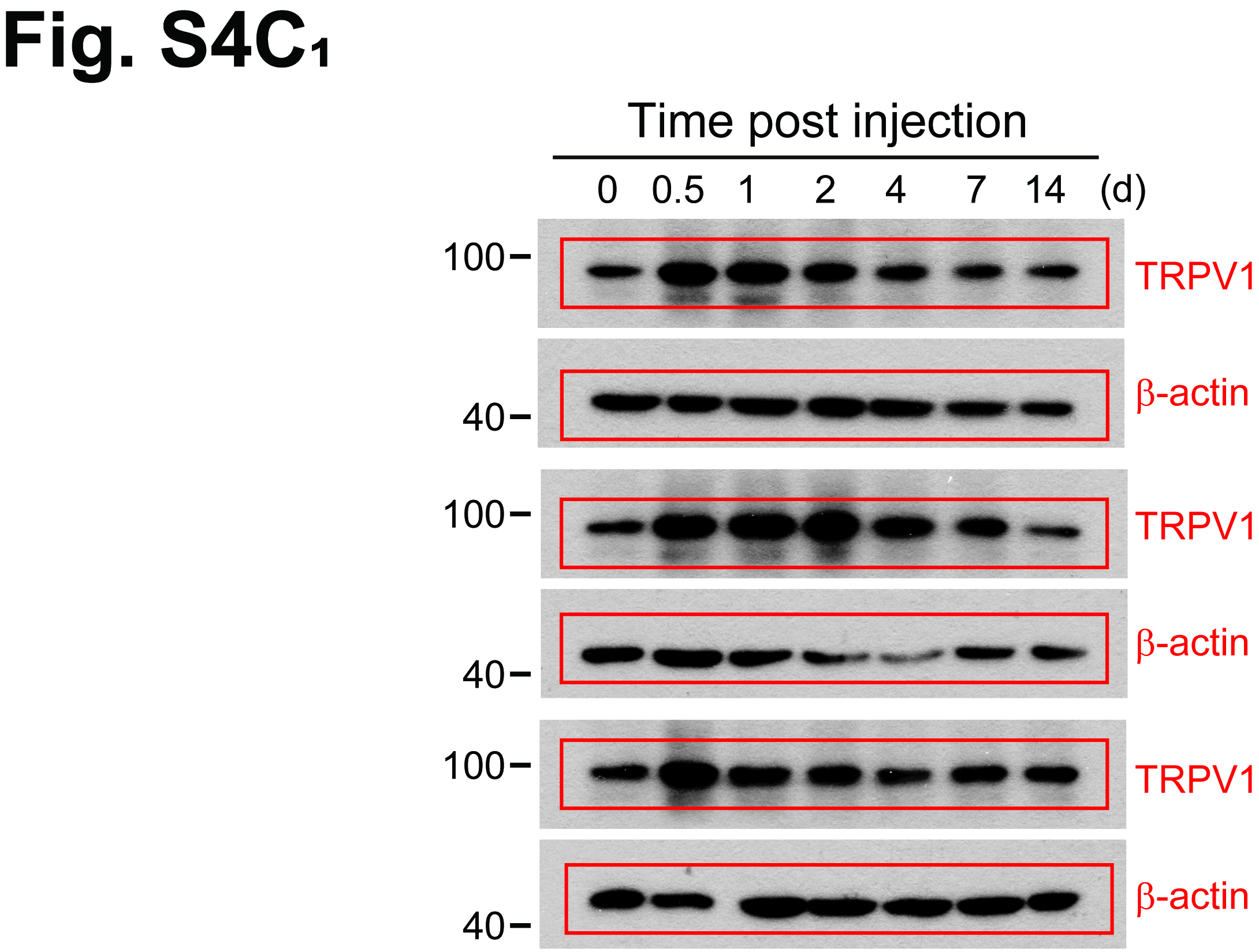

Supplement: Supplementary file 11 — Appendix Figures Source Data [file 44319_2024_317_MOESM11_ESM.zip › Appendix Source Data/Appendix Figure S4/Appendix Figure S4C.tif]

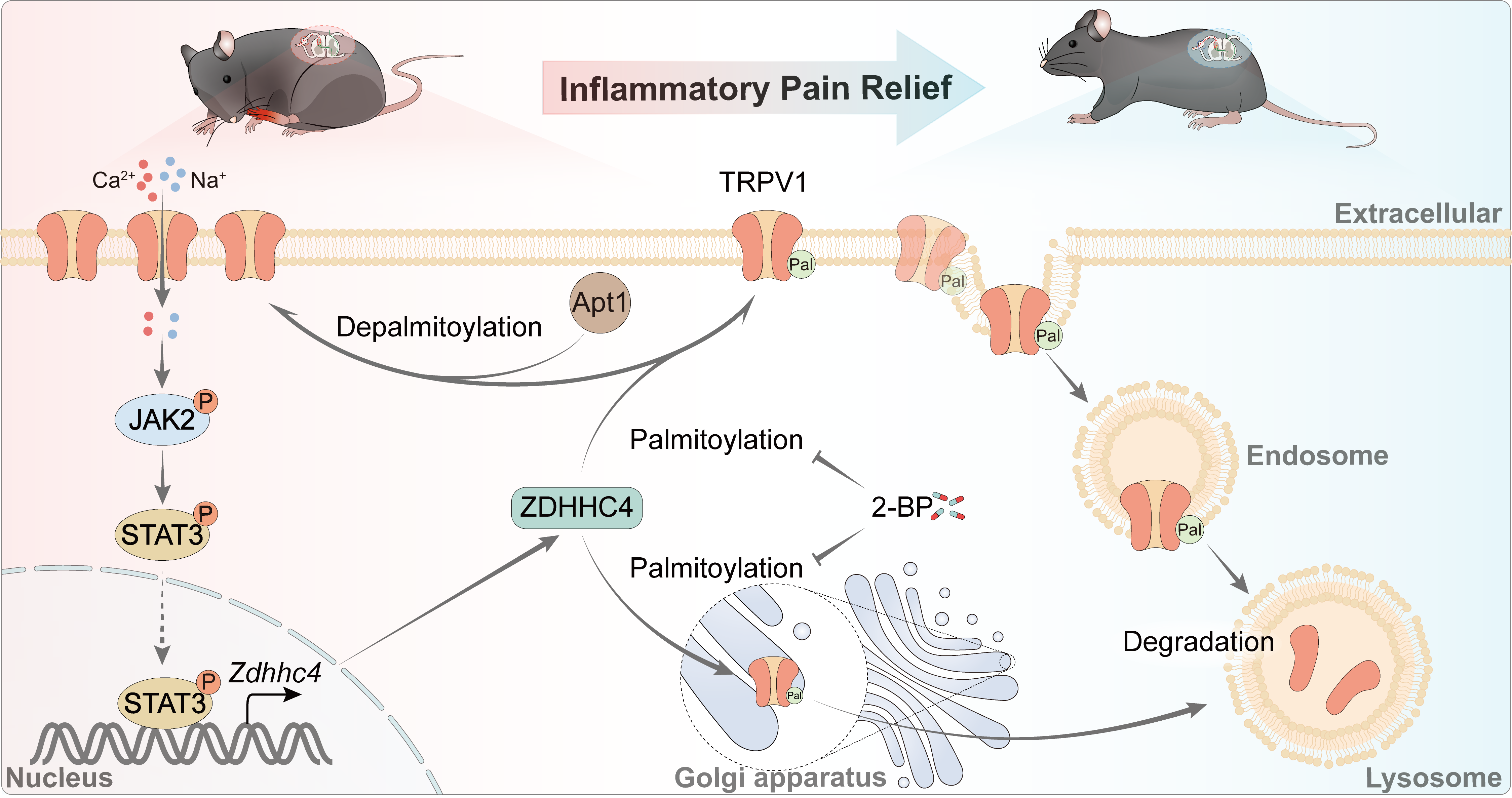

Supplement: Supplementary file 11 — Appendix Figures Source Data [file 44319_2024_317_MOESM11_ESM.zip › Appendix Source Data/Appendix Figure S5/Figure S5-Pattern diagram.tif]
